# Rehabilitation interventions for improving balance following stroke: An overview of systematic reviews

**DOI:** 10.1371/journal.pone.0219781

**Published:** 2019-07-19

**Authors:** Chiara Arienti, Stefano G. Lazzarini, Alex Pollock, Stefano Negrini

**Affiliations:** 1 IRCCS Fondazione Don Carlo Gnocchi, Milan, Italy; 2 Nursing, Midwifery and Allied Health Professions Research Unit, Glasgow Caledonian University, Glasgow, United Kingdom; 3 Department of Clinical and Experimental Sciences, University of Brescia, Brescia, Italy; University of South Australia, AUSTRALIA

## Abstract

**Background:**

The aim of this study was to synthesize evidence from systematic reviews, to summarise the effects of rehabilitation interventions for improving balance in stroke survivors.

**Methods:**

We conducted an overview of systematic reviews (SRs). We included Cochrane Systematic Reviews and non-Cochrane Systematic Reviews of randomized-controlled clinical trials and not-randomized clinical trials, in all types of stroke, comparing the effects of interventions, control interventions and no interventions on balance-related outcomes. We conducted a comprehensive search of electronic databases, from inception to December 2017. Data extracted included: number and type of participants, type of intervention, control intervention, method of assessing risk of bias of primary studies, balance outcome measures and results of statistical meta-analyses. Methodological quality of included reviews was assessed using AMSTAR 2. A narrative description of the characteristics of the SRs was provided and results of meta-analyses summarised with reference to their methodological quality.

**Results:**

51 SRs (248 primary studies and 10,638 participants) met the inclusion criteria and were included in the overview. All participants were adults with stroke. A wide variety of different balance and postural control outcomes were included. 61% of SRs focussed on the effectiveness of physical therapy, 20% virtual reality, 6% electromechanical devices, 4% Tai-Chi, whole body vibration and circuit training intervention, and 2% cognitive rehabilitation. The methodology of 54% of SRs were judged to be of a “low or critically low” quality, 23% “moderate” quality and 22% “high” quality.

**Conclusions:**

There are 51 SRs of evidence relating to the effectiveness of interventions to improve balance in people with stroke, but the majority of these are of poor methodological quality, limiting our ability to draw clear implications. Only 22% of these SRs were judged to be of high quality, highlighting the need to address important methodological issues within rehabilitation research.

## Introduction

Stroke is defined by the World Health Organization (WHO) as “a clinical syndrome consisting of rapidly developing clinical signs of focal (or global in case of coma) disturbance of cerebral function lasting more than 24 hours or leading to death with no apparent cause other than a vascular origin” and it is a leading cause of death and disability in many Western nations [1]. In Australia, the UK and the USA, stroke represents one of the 10 main causes of long-term physical disability [2–5].

The main deficit caused by stroke is motor impairment, which can be described as loss or limitation of muscle control function or movement, or limitation in mobility. It typically affects the control of movement of the face, arm and leg on one side of the body and is present in about 80% of patients [6]. Almost two-thirds of stroke survivors have initial mobility deficits, and six months after stroke, more than 30% of survivors still cannot walk independently [7]. Walking difficulties can have a major impact on stroke survivors, limiting ability to independently perform daily activities and having a negative impact on quality of life. Loss of balance when walking is common after stroke, with 70% of stroke survivors living at home reported to fall within a year of their stroke [8]. Muscle weakness and loss of voluntary movements are common problems immediately following a stroke and these contribute to reduced walking speed, which is a characteristic sign of post-stroke gait [9]. Marked temporal and spatial inter-limb asymmetries are also common, occurring in 48% to 82% and 44% to 62% of post-stroke subjects respectively; these asymmetries are correlated with impaired standing balance control during gait [10].

Generally, a key rehabilitation goal for stroke survivors is to improve walking, in order to enhance opportunities for participation in social activities and return to work [7]. Various rehabilitation approaches, founded on theories and knowledge of motor recovery and brain neuroplasticity[11], have been used to improve balance and, consequently, gait after stroke. However, there continues to be considerable controversy and debate about the relative effectiveness of different approaches to rehabilitation [12]. In order to provide optimal rehabilitation to an individual stroke survivor, a health professional needs to be able to select the most appropriate intervention, based on knowledge of the evidence of effectiveness of different interventions, and taking into account patient preference, resources and clinical setting [13].

Evidence relating to the effectiveness of interventions to improve balance is often synthesised within intervention-specific reviews, where measures of balance are reported as one of many (generally secondary) outcomes. The quantity, focus and structure of these systematic reviews (SRs) arguably create barriers to access and interpretation of evidence relating to the relative effect of different rehabilitation interventions on balance, and consequently these reviews often fail to support efficient healthcare decision making. An overview of reviews has the potential to enhance access to evidence which is dispersed across multiple SRs. This relatively new methodological approach provides a way to systematically synthesise evidence of the effect of a range of different interventions on one specific outcome, such as balance. Overviews have been developed to address the growing problem of information overload, providing a way to filter large bodies of complex evidence in order to inform healthcare decision-making [14].

Therefore, the aim of this study was to systematically synthesise evidence from systematic reviews in order to summarise the effects of rehabilitation interventions for improving balance in stroke survivors.

## Materials and methods

This overview was carried out in accordance with the latest guidance from the Cochrane Handbook for Systematic Reviews of Interventions [15] and reported following the Preferred Reporting Items for Systematic Reviews and Meta-analysis (PRISMA) statement [16]. All analyses were based on previous published studies, and thus no ethics approval or patient consent were required. The overview protocol was registered on PROSPERO (no. CRD42018095998).

### Search strategy

The search strategy involved searching the following electronic databases: MEDLINE (Pubmed), EMBASE, Cochrane Library, CINAHL, PsycINFO, Campbell Systematic Reviews, Database of Abstracts of Reviews of Effects, Epistemonikos, Joanna Briggs Institute Database of Systematic Reviews and implementation Reports and International prospective register of systematic reviews, from inception until December 2017. The following keywords were used, customized for each database using the Patient, Intervention, Comparison/control, Outcomes (PICO) approach: “stroke”, “balance”, “rehabilitation, “postural control”, with the filters: “systematic review”. There were no date or language restrictions. Further, we hand-searched key Governmental and organizational websites (such as: Evidence for Policy and Practice Information and Co-ordinating Centre, National Institute for health and Care Excellence, The Community Guide) and the reference lists of included studies. The complete search strategy is reported in the **S1 Table**.

Following completion of our overview, one reviewer updated the searches from January 2018 to May 2019, identified potentially new SRs, and judged whether review findings were likely to change the conclusions of this overview. As it was judged that there was unlikely to be any impact on our overview conclusions, potentially new SRs have not been integrated into the overview results but, for transparency, have been referenced and discussed in the ‘limitations’ section.

### Selection criteria

Two reviewers independently reviewed the citations identified in the search, and full text articles of potentially relevant studies were obtained and assessed for inclusion. In instances of disagreement between the 2 reviewers, eligibility was resolved through discussion with a third reviewer.

The inclusion criteria are described below.

#### Type of studies

We included all Cochrane Systematic Reviews (CSRs) and non-Cochrane systematic reviews (non-CSRs) of randomized-controlled clinical trials and not-randomized clinical trials, that collated empirical evidence, and met our pre-specified eligibility criteria. These criteria included that the systematic review aimed to answer a specific research question, and used explicit and systematic methods to minimize bias, thus providing reliable findings from which conclusions can be drawn and decisions made [17].

#### Type of participants

We included systematic reviews regardless of whether they combined data within meta-analyses or not, in which the participants were adults and had any type of stroke (acute, sub-acute, and chronic), in accordance with the WHO definition. We excluded systematic reviews which included participants who had diseases other than stroke which could impact on balance, such as Parkinson’s disease, cerebral traumas, multiple sclerosis, medications, ear infections and other infections, benign paroxysmal positional vertigo or positional vertigo, labyrinthitis, Ménière’s disease, vestibular neuritis, perilymph fistula, mal de Debarquement syndrome, arthritis, eye muscle imbalance.

#### Type of interventions

We included all rehabilitation interventions that were aimed at promoting balance during maintenance of a posture, restoration of a posture or movement between postures and during gait, including orthosis and excluding prosthetics. Further, we also included interventions which were focused on improving physical functioning and motor impairment in which balance was an outcome. We excluded non-rehabilitation interventions, such as surgery and/or pharmacological treatments. We did not place any restrictions on the setting in which the intervention was delivered, or on the timing of the intervention (i.e. stage of recovery or length of time post stroke).

#### Types of outcome measures

We pre-defined the following as relevant balance outcome measures:

1. General balance outcomes: Berg Balance Scale (BBS), Tinetti balance scale, Brunel balance assessment (BBA).
2. Risk of falls scale: Falls Efficacy Scale (FES).
3. Sitting balance control: Trunk Impairment Scale (TIS) and Motor Assessment Scale (MAS).
4. Standing and static balance: stabilometry platform and postural sway indicated by balance outcome measures.
5. Dynamic balance assessment tests: Timed Up & Go test (TUGT) and Step test (ST).
6. Dynamic balance assessment devices: these include devices which perturb balance, such as balance boards or moving platforms, and involve assessing response to different types of perturbation, such as sudden perturbation or continuous perturbation, using a range of different types of dynamic or static conditions [18].

For inclusion, systematic reviews had to report data relating to at least one of these outcomes of interest. We also evaluated the number of adverse events as an additional outcome, but reporting of adverse events was not an inclusion criteria.

### Data extraction and management

One reviewer utilized a standardized form to conduct the data extraction. Data extracted was independently checked by a second reviewer, and any disagreements were resolved through discussions with a third reviewer.

Specifically, information collected included:

- Systematic review publication details: title, authors and year of publication.
- Number, type and characteristics of included studies.
- Number and characteristics of participants
- Rehabilitation intervention details: type, dose, intensity and frequency
- Control intervention details: type, dose, intensity and frequency
- Method of assessment of quality of the primary studies included in each SR
- Balance outcome measures
- Results of systematic reviews and any meta-analyses: effect size, standard deviations and measures of heterogeneity, and statistical significance of results.

All extracted data were summarised within tables and/or graphical representations.

### Assessment of methodological quality of included reviews

We used the AMSTAR 2 [19] tool to assess the methodological quality of the included SRs. This was a change to our published protocol in which we stated that we intended to use the ROBIS assessment tool [20]. This change was informed by evidence suggesting that AMSTAR 2 may be easier to apply, whilst maintaining similar measurement properties as the ROBIS [21,22]. The AMSTAR 2 [19] is not designed to generate an overall ‘score’ and it is important to note that a high score may disguise critical weaknesses in specific domains. Critical weaknesses could relate to: failure to register a protocol before commencement of the review (item 2); adequacy of the literature search (item 4); justification for excluding individual studies (item 7); risk of bias (RoB) of individual studies included in the review (item 9); appropriateness of meta-analytical methods (item 11); consideration of RoB when interpreting the results of the review (item 13); assessment of presence and likely impact of publication bias (item 15). We used a process of considered judgement to interpret weaknesses detected by these critical items and to reach consensus on the methodological quality of the included reviews. Two independent assessors (CA, SGL) applied this instrument to all included systematic reviews, with any disagreements resolved through discussion with a third assessor (SN).

### Data synthesis

We produced a narrative description of the characteristics of the included SRs. We also considered differences between reviews in relation to the: participants, interventions, duration of follow-up, and type of data analysis. We synthesized the main findings relating to the effects of the interventions studied, with reference to the methodological quality of included SRs. We grouped our synthesised evidence within the following categories:

- Systematic Reviews with high methodological quality
- Systematic Reviews with moderate methodological quality
- Systematic Reviews with low and critically low methodological quality

## Results

**Fig 1:**
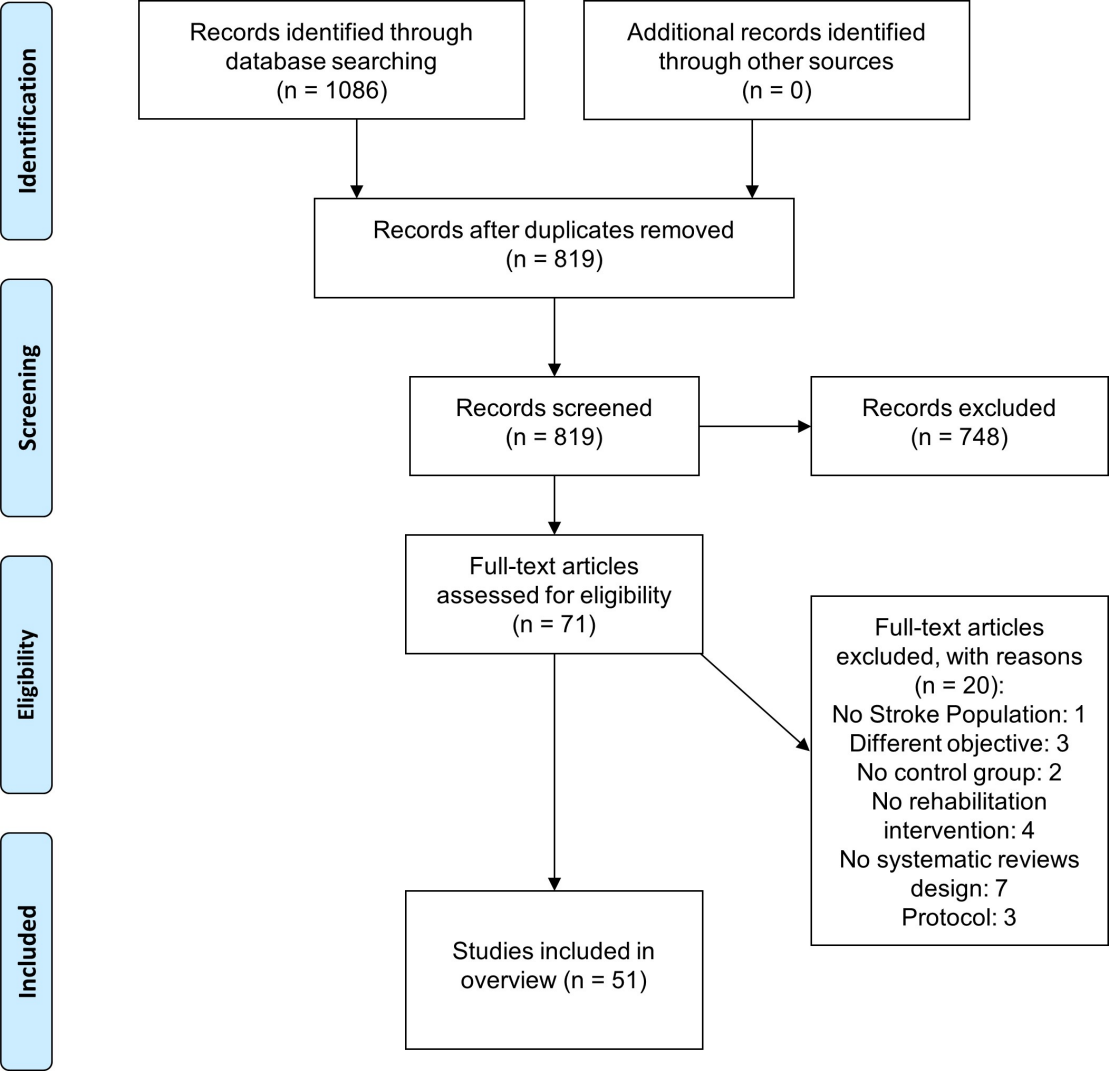
Study flow-chart.

Our search identified 1086 SRs and, after duplicates were removed and eligibility screened against the inclusion and exclusion criteria, 1016 were excluded at the title and abstract stage. Seventy-one full-text articles were obtained and screened, with 51 SRs meeting the inclusion criteria and therefore included in this overview (**Fig 1**). Of these, 39 were non-CSRs and 12 were CSRs.

### Description of included reviews

We included a total of 51 SRs (excluding duplicates, 248 primary studies and 10,638 participants) in which all participants were adults with ischemic and haemorrhagic stroke in all stages (acute, subacute and chronic). 46 of the included SRs only included randomized-controlled trials (RCTs), while 5 included non-randomised evidence (NRCTs) in addition to RCTs. The number of included trials within each SR ranged from 2 to 64 with a mean of 9.96±10.91, and the number of participants in these trials ranged from 3 to 250 with a mean of 40.09±34.20. 39 of the included SRs conducted a meta-analysis relevant to this overview (i.e. reporting an outcome relevant to balance). The outcomes for which data were combined within meta-analyses included Berg Balance Scale (BBS), TUG test (TUGT), Tinetti score, sitting and standing balance and centre of pressure sway (**Table 1**). 61% of SRs focused on the effectiveness of physical therapy as defined by World Confederation for Physical Therapy [23], 20% focused on virtual reality, 6% on electromechanical device, 4% on whole body vibration, Tai-Chi interventions and interventions for eye movement and visual field defects, 2% on cognitive rehabilitation (**S2 Table)**. See **S3 Table** for the complete list of all included SRs, and trials included in these SRs, which have contributed to this overview. See **S4 Table** for the characteristics of the included SRs, including full details of the participants, interventions, comparisons and outcomes. No study reported any adverse events of rehabilitation interventions.

**Table 1:** Overview of key characteristics of included reviews.

Review	Trials (n)	Interventions	Methodology quality assessment	Outcomes	AMSTAR judgement of review quality
***Cochrane Reviews (CSRs)***					
Barclay-Goddard 2004	7 (245)	Visual or auditory force platform feedback	Jadad (1–3)	BBS, TUGT, Centre of Pressure Position (Stance symmetry), Centre of Pressure Behaviour (sway)	Moderate
Bowen 2013	0	Cognitive rehabilitation	RoB Cochrane Tool (NR)	BBS, FRT, ST, Get up and Go test, Standing Balance test	High
English 2017	11 (935)	Circuit class therapy	RoB Cochrane Tool (NR)	TUG, BBS, ST and ABC Scale	High
French 2016	14 (766)	Repetitive task training	RoB Cochrane Tool (NR)	Sitting balance/reach: Reaching distance, Sitting Equilibrium Index, MAS—Balance Sitting subscale, Lateral reach—time to return to quiet sitting Standing balance/reach: BBS, Upright Equilibrium Index, FRT, ABC Scale, TBT	High
Laver 2017	13 (320)	Virtual reality	RoB Cochrane Tool (NR)	BBS, BBA, FRT, POMA, Forward reach test, FES, PASS, BPM	High
Lawrence 2017	2 (69)	Yoga	RoB Cochrane Tool: high risk of bias	BBS, ABC Scale	High
Mehrholz 2011	2 (38)	Water-based exercises	RoB Cochrane Tool (NR) PEDro Scale (5–6)	BBS	High
Pollock 2011 (a)	0	Interventions for eye movement disorders	RoB Cochrane Tool (NR)	BBS, FRT, ST, Get up and Go test, Standing Balance test	High
Pollock 2011 (b)	0	Intervention specifically targeted at improving the visual field defect or improving the ability of the participant to cope with the visual field loss	RoB Cochrane Tool (NR)	BBS, FRT, ST, Get up and Go test, Standing Balance test	High
Pollock 2014	11 (509)	Physical rehabilitation	RoB Cochrane Tool (NR)	BBS	High
Saunders 2016	19 (1128)	Physical fitness training	RoB Cochrane Tool (NR)	BBS, FRT, TIS, Four Square Step Test, TBT, Postural Sway	High
Vloothuis 2016	3 (139)	Caregiver-mediated exercise in addition to usual care or instead of usual care	RoB Cochrane Tool: 1 unclear risk of bias, 2 low risk of bias	BBS, PASS	High
***Non-Cochrane Reviews (non-CSRs)***					
An 2011	10 (650)	Exercise-Based Rehabilitation	PEDro Scale (4–8): 7 High quality, 3 Lower quality	BBS, Shifting centre of gravity movements, TUGT, SRT, COP variability and total excursion	Critically low
Bank 2016	11 (428)	Additional physiotherapy to standard physiotherapy	PEDro Scale (4–7)	TCT, TIS, PASS-TC, Symmetry Index, MAS—Balance Sitting subscale, Sitting Equilibrium Index	Low
Bonini-Rocha 2018	3 (174)	Circuit-based exercise	PEDro Scale (5–8): 2 High quality, 1 low quality RoB Cochrane Tool (NR)	BBS	Moderate
Cabanas-Valdés 2013	11 (308)	Trunk training exercises	PEDro Scale (3–8): 6 High quality, 5 low quality	mRT, TIS—Static Sitting Balance subscale, TIS—Dynamic Sitting Balance subscale, TIS—Coordination subscale, BWD, Romberg test, BBS, FTBS, Tinetti score, BBA	Critically low
Chen BL 2015	9 (833)	Traditional Chinese exercises	RoB Cochrane Tool: High risk of bias	BBS, TUGT, FMA—Balance subscale, limit of stability, SOT, SPPB—Balance subscale	Moderate
Chen J 2015	2 (54)	Telerehabilitation	RoB Cochrane Tool: Low risk of bias	BBS	Low
Chen L 2016	5 (204)	Sling exercise training	Modified Jadad Scale	BBS, SA, SL, Bio Rescue measures, PASS	Critically low
Chen Ling 2016	9 (265)	Virtual reality	PEDro Scale (4–9): 2 fair quality, 6 good quality, 1 excellent quality	BBS, TUGT, Static and Dynamic balance assessed by force platform	Critically low
Cheok 2015	2 (42)	Additional Wii	PEDro Scale (5–8): 1 good quality, 1 fair quality RoB Cochrane Tool (NR)	BBS, Postural sway measures (AP eyes open and closed, ML eyes open and closed)	Low
Corbetta 2015	9 (216)	Virtual reality based rehabilitation replacing some or all of standard rehabilitation or virtual reality based rehabilitation used as extra rehabilitation time added to a standard rehabilitation regimen	RoB Cochrane Tool (NR)	BBS	Low
de Rooji 2016	18 (433)	Balance training using Virtual reality	PEDro Scale (3–8): 11 High quality, 7 Lower quality	BBS, TUGT	Moderate
Dos Santos 2015	3 (54)	Rehabilitation with Nintendo Wii	PEDro Scale (4–7)	BBS, TUGT, Pressure platforms	Critically low
Ge 2017	21 (1408)	Traditional Chinese exercises	RoB Cochrane Tool (NR)	BBS, TUGT, FMA—Balance subscale	Low
Hammer 2008	14 (638)	Physiotherapy interventions aimed at restoring balance without extensive technical equipment	PEDro Scale (6–8): 6 High quality, 6 Medium quality, 2 Low quality	BBS, FRT, TUGT, MAS, STREAM, RMI, Posturography, ST, SRT	Critically low
Hancock 2012	2 (62)	Lower limb reciprocal pedalling exercise	RoB Cochrane Tool (NR)	PASS, PASS—Static subscale, PASS—Dynamic subscale, BBS, Get Up and Go	Low
Iruthayarajah 2017	20 (468)	Virtual reality (isolated or in combination with other therapies)	PEDro Scale (5–8): 17 good quality, 3 fair quality	Dynamic Balance: BBS, TUGT, FRT, 6MWT, 1MWT, 10MWT, 3MWT, ART, TST, 30SST, POMA, BBA Static Balance: COP path lengths and oscillations, Limit of stability, Postural sway path length and velocity, Stability Index, BWD, Symmetry Index	Critically low
Ko 2014	6 (168)	Lumbar stabilization exercises (on unstable bases of support)	PEDro Scale (5–8)	TIS, TIS—Static Sitting Balance subscale, TIS—Dynamic Sitting Balance subscale, BBS, BBA, FRT, Tinetti test, Romberg eyes open, Romberg eyes closed, FTBS, SA, SP	Critically low
Kollen 2009	4 (224)	Bobath Concept	PEDro Scale (4–8): 4 High quality	BWD over hemiplegic and non-hemiplegic sides, MAS, BBS	Critically low
Langhorne 2009	12 (465)	Interventions for motor recovery	N/R	BBS, BWD, Postural sway during sitting and standing	Moderate
Li 2016	14 (334)	Virtual reality	RoB Cochrane Tool (2–4): 3 valued 2 points, 5 valued 3 points, 5 valued 4 points	BBS, TUGT, FRT, ABC Scale, BBA, Tinetti Gait and Balance Test, Sway velocity, BWD	Moderate
Lin 2018	2 (67)	Transcutaneous electrical nerve stimulation (TENS)	Jadad Scale: High quality	TUGT, Postural sway velocity	Low
Lu 2015	3 (133)	Whole Body Vibration	RoB Cochrane Tool (NR)	BBS	Critically low
Lubetzky-Vilnay 2010	20 (725)	Balance training	American Academy of Cerebral Palsy and Developmental Medicine Scale: 5-point scale from Level I to level V; within each level, quality was assessed based on 7 internal and external validity characteristics (Level I-IV, quality rating 4,5–7)	BBS, Postural Control and Balance Test, force platform measures of balance index, dynamic limits of stability, Brunnstrom stage, Number of falls, FMA—Balance subscale, Balance Index on the Kinesthetic Ability Trainer, COP displacement, ABC Scale, FES, DGI	Critically low
Luque-Moreno 2015	4 (99)	Virtual reality	PEDro Scale (6–7)	BBS, ART, BBA, TUGT, ST, TST, 1MWT, 10MWT, 30SST, BPM, Postural sway, FMA	Critically low
Sorinola 2014	2 (53)	Additional trunk exercises	RoB Cochrane Tool: one moderate and one low risk of bias PEDro Scale (6–7)	Upright equilibrium index, Tinetti Scale	Low
Stoller 2012	3 (163)	Early cardiovascular exercise	PEDro Scale (6–8): good quality	BBS, FRT	Moderate
Swinnen 2014	9 (359)	Robot-assisted gait training	Evaluation of Quality of an Intervention Study checklist (56–81%) 7 true experimental, 3 pre-experimental studies	BBS, TUGT, Tinetti test, Postural sway tests, Romberg test	Critically low
Tally 2017	8 (275)	Treadmill training, isolated or with adjunctive interventions	PEDro Scale (5–9): 7 High quality, 1 Lower quality	BBS, combination of directional postural sway and limits of stability assessment	Critically low
Tang 2015	19 (729)	Interventions on improving balance self-efficacy	PEDro Scale (3–8): 1 poor, 4 fair, 14 good quality	Balance self-efficacy: ABC Scale, FES-International and FES-Swedish version	Low
Tyson 2013	5 (183)	Walking with Ankle-Foot Orthosis	RoB Cochrane Tool: low risk of bias	BBS, Postural sway, BWD while standing	Moderate
Van Criekinge 2018	7 (184)	Trunk rehabilitation using unstable surfaces	PEDro Scale (4–8): 6 high risk of bias and 1 low risk of bias	Sitting balance: TIS, TIS—Static Sitting Balance subscale, TIS—Dynamic Sitting Balance subscale, TIS—Coordination subscale, MAS—Balance Sitting subscale Standing balance: BBS, centre of gravity displacements, BBA—Standing subscale, MAS—Sitting to standing subscale, FICSIT-4	Moderate
van Duijnhoven 2016	43 (1522)	Exercise therapy	PEDro Scale (4–9): 34 High quality, 9 Moderate quality	BBS, FRT, SOT, Mean postural sway velocity	Moderate
Van Peppen 2004	20 (658)	Physical therapy	PEDro Scale (4–7)	Postural symmetry sit-to-stand (BWD, vertical force difference between left and right, peak vertical ground reaction force through affected foot), Postural symmetry stand-to-sit (BWD, vertical force difference between left and right), Time needed to stand-up, Time needed to sit-down, Postural sway/symmetry, BBS, TUGT	Critically low
Van Peppen 2006	7 (177)	Bilateral standing with visual feedback therapy	PEDro Scale (3–6)	BWD while bilateral standing, postural sway in bilateral standing, BBS, TUGT	Critically low
Veerbeek 2014	64 (2469)	Physical therapy	PEDro Scale (2–8)	BBS, BBA, PASS, ST, FRT, LRT, TIS, SRT, FMA, BWD, STS, SST, ABC Scale, Sitting and standing symmetry, Sitting equilibrium test, Reach distance, Posturography, Static balance, Dynamic balance, Tinetti, Postural sway	Moderate
Wang 2015	9 (276)	Cognitive motor interference	RoB Cochrane Tool: High Risk of bias	SA, SD, BBS, TUGT, ABC Scale	Low
Wevers 2009	5 (241)	Circuit class training	PEDro Scale (4–8): high quality	BBS, ST	Critically low
Wist 2016	7 (291)	Strengthening of the lower limbs	RoB Cochrane Tool (NR)	BBS, TUGT	Low
Yang 2015	4 (186)	Whole Body Vibration	RoB Cochrane Tool (NR)	BBS	Moderate

10MWT = 10-Meter Walking Test; 1MWT = 1-Minute Walking Test; 30SST = 30-Second Sit to Stand Test; 3MWT = 3-Meter Walking Test; 6MWT = 6-Minute Walking Test; ABC Scale = Activities Based Confidence Scale; AP = Anteroposterior; ART = Anterior Reach Test; BBA = Brunel Balance Assessment; BBS = Berg Performance Scale; BPM = Balance Performance Monitor; BWD = Body Weight Distribution; COP = Centre of Pressure; DGI = Dynamic Gait Index; FES = Falls Efficacy Scale; FICSIT-4 = Frailty and Injuries Cooperative Studies of Intervention Technique scale; FMA = Fugl-Meyer Assessment; FRT = Functional Reach Test; FTBS = Four Test Balance Scale; MAS = Motor Assessment Scale; ML = Mediolateral; mRT = Modified Reach Test; PASS = Postural Assessment Scale for Stroke patients; PASS-TC = Postural assessment scale for stroke patients—Trunk Control; POMA = Tinetti Performance Oriented Mobility Assessment; RMI = Rivermead Mobility Index; SA = Sway Area of the COP; SD = Sway Distance of the COP; SL = Sway Length of the COP; SOT = Sensory Organization Test; SP = Sway Path of the COP; SPPB = Short Physical Performance Battery; SRT = Step Reaction Time; SST = Single Support Time; ST = Step Test; STREAM = Stroke Rehabilitation Assessment of Movement; STS = Sit-to-stand; TBT = Timed Balance Test; TCT = Trunk Control Scale; TIS = Trunk Impairment Scale; TST = Timed Stair Test; TUGT = Timed Up and Go Test.

### Methodological quality of included reviews

The results of AMSTAR 2 assessment are reported in **Table 2**. Our findings show that the main weakness was lack of protocol registration and there was generally very poor reporting, with only 16% of SRs adequately adhering to PRISMA reporting guidelines. 61% did not justify reasons for excluding individual studies and did not consider the RoB assessment when interpreting the results of the review. 63% did not evaluate the risks of publication bias but, despite that, 75% conducted meta-analysis using an appropriate meta-analytical method.

**Table 2:** AMSTAR-2 assessment.

Reference	AMSTAR-2 Domains																
	1	2	3	4	5	6	7	8	9	10	11	12	13	14	15	16	Overall quality
**Cochrane Systematic Reviews**																	
*Barclay-Goddard 2004*	Y	N	Y	Y	Y	Y	Y	Y	Y	N	Y	N	N	Y	N	Y	moderate
*Bowen 2013*	Y	Y	Y	Y	Y	Y	Y	Y	Y	N	Y	Y	Y	Y	Y	Y	high
*English 2017*	Y	Y	Y	Y	Y	Y	Y	Y	Y	N	Y	Y	Y	Y	Y	Y	high
*French 2016*	Y	Y	Y	Y	Y	Y	Y	Y	Y	N	Y	Y	Y	Y	Y	Y	high
*Laver 2017*	Y	Y	Y	Y	Y	Y	Y	Y	Y	N	Y	Y	Y	Y	Y	Y	high
*Lawrence 2017*	Y	Y	Y	Y	Y	Y	Y	Y	Y	N	Y	Y	Y	Y	Y	Y	high
*Mehrholz 2011*	Y	Y	Y	Y	Y	Y	Y	Y	Y	N	Y	Y	Y	Y	Y	Y	high
*Pollock 2011a*	Y	Y	Y	Y	Y	Y	Y	Y	Y	N	Y	Y	Y	Y	Y	Y	high
*Pollock 2011b*	Y	Y	Y	Y	Y	Y	Y	Y	Y	N	Y	Y	Y	Y	Y	Y	high
*Pollock 2014*	Y	Y	Y	Y	Y	Y	Y	Y	Y	N	Y	Y	Y	Y	Y	Y	high
*Saunders 2016*	Y	Y	Y	Y	Y	Y	Y	Y	Y	N	Y	Y	Y	Y	Y	Y	high
*Vloothuis 2016*	Y	Y	Y	Y	Y	Y	Y	Y	Y	N	Y	Y	Y	Y	Y	Y	high
**non-Cochrane Systematic Reviews**																	
*An 2011*	Y	N	Y	PY	N	N	PY	PY	Y	N	NMA	NMA	N	N	NMA	Y	critically low
*Bank 2016*	Y	Y	Y	PY	Y	Y	PY	PY	Y	N	Y	N	N	N	N	Y	low
*Bonini-Rocha 2018*	Y	N	Y	Y	Y	N	Y	Y	Y	N	Y	N	N	N	Y	Y	moderate
*Cabanas-Valdés 2013*	Y	N	Y	Y	Y	Y	PY	PY	Y	N	NMA	NMA	N	Y	NMA	Y	critically low
*Chen BL 2015*	Y	PY	Y	PY	Y	Y	PY	PY	Y	N	Y	Y	Y	Y	Y	Y	moderate
*Chen J 2015*	N	N	Y	PY	Y	Y	PY	PY	Y	N	Y	Y	Y	Y	N	Y	low
*Chen L 2016*	Y	N	Y	PY	Y	Y	PY	PY	Y	N	Y	N	N	N	N	Y	critically low
*Chen Ling 2016*	Y	N	N	PY	Y	N	N	PY	Y	N	NMA	NMA	Y	N	NMA	Y	critically low
*Cheok 2015*	N	N	Y	PY	Y	Y	Y	PY	Y	N	Y	N	N	N	N	Y	low
*Corbetta 2015*	Y	N	N	Y	Y	Y	N	Y	Y	N	Y	N	N	N	N	Y	low
*de Rooji 2016*	Y	N	Y	Y	Y	Y	Y	Y	Y	N	Y	N	N	N	N	Y	moderate
*Dos Santos 2015*	Y	Y	Y	PY	Y	Y	PY	PY	Y	N	NMA	NMA	N	N	NMA	Y	critically low
*Ge 2017*	Y	N	N	PY	Y	Y	PY	PY	Y	N	Y	N	N	Y	Y	Y	low
*Hammer 2008*	N	PY	Y	PY	Y	Y	PY	PY	Y	N	NMA	NMA	N	N	NMA	Y	critically low
*Hancock 2012*	Y	PY	Y	Y	Y	Y	PY	Y	Y	N	NMA	NMA	Y	Y	NMA	Y	low
*Iruthayarajah 2017*	Y	PY	N	PY	N	Y	PY	Y	Y	N	Y	N	N	N	N	Y	critically low
*Ko 2014*	N	N	N	N	Y	Y	N	N	Y	N	NMA	NMA	N	N	NMA	Y	critically low
*Kollen 2009*	Y	N	N	PY	Y	N	PY	PY	Y	N	NMA	NMA	N	Y	NMA	Y	critically low
*Langhorne 2009*	Y	N	Y	Y	Y	Y	Y	Y	Y	N	Y	Y	Y	Y	N	Y	moderate
*Li 2016*	Y	Y	Y	Y	Y	Y	Y	Y	Y	N	Y	N	N	Y	N	Y	moderate
*Lin 2018*	Y	Y	N	PY	N	N	N	PY	Y	N	Y	N	N	N	N	Y	low
*Lu 2015*	N	N	N	PY	N	N	PY	PY	Y	N	Y	N	N	N	Y	Y	critically low
*Lubetzky-Vilnay 2010*	Y	N	N	PY	N	N	N	PY	PY	N	NMA	NMA	N	N	NMA	Y	critically low
*Luque-Moreno 2015*	N	N	Y	PY	Y	N	PY	PY	Y	N	NMA	NMA	N	N	NMA	Y	critically low
*Sorinola 2014*	Y	Y	Y	PY	Y	Y	PY	PY	Y	N	Y	N	N	Y	N	Y	low
*Stoller 2012*	Y	PY	Y	Y	Y	Y	Y	Y	Y	N	Y	N	N	Y	N	Y	moderate
*Swinnen 2014*	N	N	N	PY	Y	N	PY	N	PY	N	NMA	NMA	N	N	NMA	Y	critically low
*Tally 2017*	Y	PY	N	PY	Y	Y	PY	PY	Y	N	NMA	NMA	N	N	NMA	Y	critically low
*Tang 2015*	N	Y	Y	PY	Y	N	PY	PY	Y	N	Y	N	N	N	N	Y	low
*Tyson 2013*	N	N	Y	PY	Y	N	N	PY	Y	N	Y	N	Y	Y	Y	Y	moderate
*Van Criekinge 2018*	Y	PY	N	Y	Y	Y	Y	PY	Y	N	Y	N	N	Y	N	Y	moderate
*van Duijnhoven 2016*	Y	PY	Y	PY	N	N	PY	PY	Y	N	Y	Y	Y	Y	Y	Y	moderate
*Van Peppen 2004*	N	N	N	PY	Y	N	PY	PY	Y	N	NMA	NMA	N	N	NMA	Y	critically low
*Van Peppen 2006*	N	N	Y	PY	Y	N	PY	N	Y	N	Y	N	N	N	N	Y	critically low
*Veerbeek 2014*	Y	N	Y	Y	Y	Y	Y	Y	Y	N	Y	Y	Y	Y	N	Y	moderate
*Wang 2015*	Y	N	Y	PY	Y	Y	PY	PY	Y	N	Y	N	N	N	Y	Y	low
*Wevers 2009*	Y	N	Y	PY	Y	Y	N	PY	Y	N	Y	N	N	N	N	Y	critically low
*Wist 2016*	Y	N	Y	Y	Y	Y	PY	PY	Y	N	Y	N	N	N	N	Y	low
*Yang 2015*	Y	N	N	Y	Y	N	PY	PY	Y	N	Y	N	Y	Y	Y	Y	moderate
*Total Yes*	78%	33%	73%	47%	88%	71%	39%	41%	96%	0%	75%	31%	39%	53%	37%	100%	

**Domains**: 1 = Did the research questions and inclusion criteria for the review include the components of PICO?; 2 = Did the report of the review contain an explicit statement that the review methods were established prior to the conduct of the review and did the report justify any significant deviations from the protocol?; 3 = Did the review authors explain their selection of the study designs for inclusion in the review? 4 = Did the review authors use a comprehensive literature search strategy?; 5 = Did the review authors perform study selection in duplicate?; 6 = Did the review authors perform data extraction in duplicate?; 7 = Did the review authors provide a list of excluded studies and justify the exclusions?; 8 = Did the review authors describe the included studies in adequate detail?; 9 = Did the review authors use a satisfactory technique for assessing the risk of bias (RoB) in individual studies that were included in the review?; 10 = Did the review authors report on the sources of funding for the studies included in the review?; 11 = If meta-analysis was performed did the review authors use appropriate methods for statistical combination of results?; 12 = If meta-analysis was performed, did the review authors assess the potential impact of RoB in individual studies on the results of the meta-analysis or other evidence synthesis?; 13 = Did the review authors account for RoB in individual studies when interpreting/discussing the results of the review?; 14 = Did the review authors provide a satisfactory explanation for, and discussion of, any heterogeneity observed in the results of the review?; 15 = If they performed quantitative synthesis did the review authors carry out an adequate investigation of publication bias (small study bias) and discuss its likely impact on the results of the review?; 16 = Did the review authors report any potential sources of conflict of interest, including any funding they received for conducting the review?

**Answers**: Y = Yes; PY = Partial Yes; N = No; NMA = No meta-analysis conducted

In summary, 55% of SRs were judged to provide “low/critically low” quality evidence, 23% to provide “moderate” quality evidence and only 22% to provide “high” quality evidence.

### Interventions studied

We synthesized the main results of the included SRs by categorising their findings according to methodological quality of included SRs, organised by groups of interventions. For further details, see **S5 Table**. We based our grouping of SRs according to types of intervention, using the terminologies and descriptions provided within each SR.

#### Systematic reviews with high methodological quality: Physical therapy

Seven SRs focussed on physical therapy interventions and were judged to be of high methodological quality. All of these were CSRs and investigated the effectiveness of different physical approaches [24,25], repetitive task training [26], caregiver-mediated exercise [27], yoga [28], water-based exercises [29], circuit class therapy [30] on balance and postural control. All of these SRs used the GRADE approach to evaluate the quality of evidence [31].

*Pollock 2014* [24] CSR reported, from 11 trials (509 participants), a significant beneficial effect of physical approaches with very low quality of evidence for the comparison of intervention versus no treatment and moderate quality evidence for the comparison of intervention versus usual care. No significant differences were found between subgroups in which the intervention included different treatment components. *French 2016* [26] CSR, based on 14 trials (766 participants), reported a statistically significant improvement of repetitive task training, with low quality of evidence. *Saunders 2016* [25] CSR, based on 19 trials (1128 participants), reported significant beneficial effects of resistance training and mixed training and no effect of cardiorespiratory training, with high quality of evidence for all comparison with balance outcomes.

*Vloothuis 2016* [27] CSR, based on 3 trials (139 participants), reported significant improvement of care-mediated exercise in addition to usual care, with low quality of evidence for balance in caregiver-mediated exercises compared with control intervention. *Lawrence 2017* [28] CSR, with 2 trials (69 participants), and *Mehrholz 2011* [29] CSR, with 2 trials (38 participants), reported no significant effect of yoga and water-based exercises, respectively, with very low quality of evidence.

*English 2017* [30] CSR, based on 11 trials (935 participants), reported significant beneficial effects of circuit class therapy with low quality of evidence.

#### Systematic reviews with high methodological quality: Other interventions

Four CSRs evaluated the effectiveness of interventions for eye movement disorders and visual field defects [32,33], virtual reality [34] and cognitive rehabilitation [35] on outcome measures of balance. *Pollock 2011a*, *b* [32,33] CSR were not able to draw conclusions from their studies on balance outcomes because they found no studies. *Laver 2017* [34] CSR, based on 13 trials (320 participants), reported significant improvement of virtual reality combined with usual care, but no improvement when comparing virtual reality to conventional therapy. GRADE judgement quality was not reported for the balance outcomes. *Bowen 2013* [35] CSR found no relevant outcome data for evaluating cognitive rehabilitation effects on balance.

#### Systematic reviews with moderate methodological quality: Physical therapy

Eight moderate-quality reviews, 1 CSRs and 7 non-CSRs, evaluated the effect of exercise therapy [36], moving platform and biofeedback using a force plate [37], ankle-foot orthosis [38], cardiovascular exercise [39], balance training, electromechanical-assisted gait training, circuit class training, mixed strength, cardiorespiratory exercise and high-intensity practice [40], trunk rehabilitation using unstable support surfaces [41], visual force platform feedback [42] and circuit-based exercise [43] on balance outcomes. *van Duijnhoven 2016* [36] non-CSR, included 43 trials (1,522 participants), evaluating the effectiveness of exercise therapy compared with usual care on BBS. They reported a significative effect of exercise therapy. *Langhorne 2009* [37] non-CSR, based on 12 trials (465 participants), reported a positive improvement of moving platform and an improvement of biofeedback using a force plate. Other interventions did not report any effect on balance outcomes. *Tyson 2013* [38] non-CSR, based on 5 trials (183 participants), reported a significant effect of walking with an ankle-foot orthosis and weight distribution while standing. No significant effects were found on measures of postural sway. *Stoller 2012* [39] non-CSR, based on 3 trials (163 participants), reported an improvement in balance after early cardiovascular exercise. *Veerbeek 2014* [40] non-CSR, based on 64 trials (2,469 participants), reported significant improvements in sitting and standing balance as a result of sitting balance training, balance training during various activities, electromechanical-assisted gait training with ES, circuit class training, mixed strength and cardiorespiratory exercise and high-intensity practice. *Van Criekinge 2018* [41] non-CSR, including 7 trials (184 participants), found that trunk rehabilitation using unstable support surfaces, except for the sling, showed larger improvements compared to stable support surfaces on balance sitting, but no consensus has been reached regarding the superiority of unstable support surfaces on standing balance. *Barclay-Goddard 2004* [42] CSR, based on 7 trials (245 participants), reported no significant improvement as a result of visual force platform feedback and *Bonini-Rocha 2018* [43] non-CSR, based on 3 trials (174 participants), found no significant improvement in response to circuit-based exercise.

#### Systematic reviews with moderate methodological quality: Other interventions

Four moderate-quality reviews investigated the effectiveness of virtual reality [44,45], traditional Chinese exercise [46] and whole-body vibration training [47] on outcome measures of balance. *de Rooij 2016* [44] non-CSR, including 18 trials (433 participants) and *Li 2016* [45] non-CSR, with 14 trials (334 participants), both found a significant improvement when a virtual reality intervention was compared with a similar time-dose of conventional intervention. They did not find significant results when virtual reality treatment was combined with conventional therapy.

*Chen 2015* [46] non-CSR, based on 9 trials (833 participants), found that traditional Chinese exercise significantly improved all balance outcomes. *Yang 2015* [47] non-CSR, with 4 trials (186 participants), showed no significant benefit of whole-body vibration training.

#### Systematic reviews with low and critically low methodological quality: Physical therapy

There were sixteen low or critically low non-CSRs which explored the effect of physical therapy interventions on balance outcomes. These provide evidence that the following interventions may have some beneficial impact on a measure of balance: trunk exercises (*Sorinola 2014* [48], 2 trials, 53 participants) and more intense physical exercise-based interventions (*Tang 2015* [49], 19 trials, 729 participants). These reviews reported no evidence of beneficial effects on balance outcomes for the following interventions: reciprocal pedaling exercise (*Hancock 2012* [50], 2 trials, 62 participants); aerobic exercise (*An 2011* [51], 10 trials, 650 participants); muscle strengthening of lower limb, progressive resistance training, aerobic exercise, task-specific training and functional electrical stimulation (FES) (*Wist 2016* [52], 7 trials, 291 participants); training sit-to-stand transfers and vice versa, training standing balance and treadmill training (*Van Peppen 2004* [53], 20 trials, 658 participants); lumbar stabilization exercises on stable and unstable surfaces (*Ko 2014* [54], 6 trials, 168 participants); trunk training exercise (*Cabanas-Valdés 2013* [55], 11 trials, 308 participants); sling exercise training (*Chen L 2016* [56], 5 trials, 204 participants); balance training and motor relearning program (*Lubetzky-Vilnai 2010* [57], 20 trials, 725 participants); additional physiotherapy to standard therapy (*Bank 2016* [58], 11 trials, 428 participants); Bobath technique (*Hammer 2008* [59], 14 trials, 638 participants, and *Kollen 2009* [60], 4 trials, 224 participants); cognitive motor interference (*Wang 2015* [61], 9 trials, 276 participants); visual feedback therapy (*Van Peppen 2006* [62], 7 trials, 177 participants); circuit class training (*Wevers 2009* [63], 5 trials, 241 participants).

#### Systematic reviews with low and critically low methodological quality: Virtual reality

There were seven low or critically low quality non-CSRs which evaluated the effectiveness of virtual reality therapy on balance outcomes. These highlighted evidence that virtual reality may have some beneficial effects on balance (*Corbetta 2015* [7], 9 trials, 216 participants, *Chen Ling* [64] *2016*, 9 trials, 265 participants and *Luque-Moreno 2015* [65], 4 trials, 99 participants). However use of the Nintendo Wii (*Cheok 2015* [66], 2 trials, 42 participants, *Dos Santos 2015* [67], 3 trials, 54 participants and *Iruthayarajah 2017* [68], 20 trials, 468 participants) and telerehabilitation (*Chen 2015* [69], 2 trials, 54 participants) were not found to result in any improvement to balance.

#### Systematic reviews with low and critically low methodological quality: Other interventions

There were five low or critically low quality non-CSRs which evaluated the effectiveness of treadmill training (*Tally 2017* [70], 8 trials, 275 participants), robot-assisted gait training (*Swinnen 2014* [71], 9 trials, 359 participants), transcutaneous electrical nerve stimulation (TENS) (*Lin 2018* [72], 2 trials, 67 participants), whole-body vibration (*Lu 2015* [73], 3 trials, 133 participants), traditional Chinese exercise (*Ge 2017* [74], 21 trials, 1408 participants); none of these reported any evidence of beneficial effects on balance outcomes.

**Fig 2:**
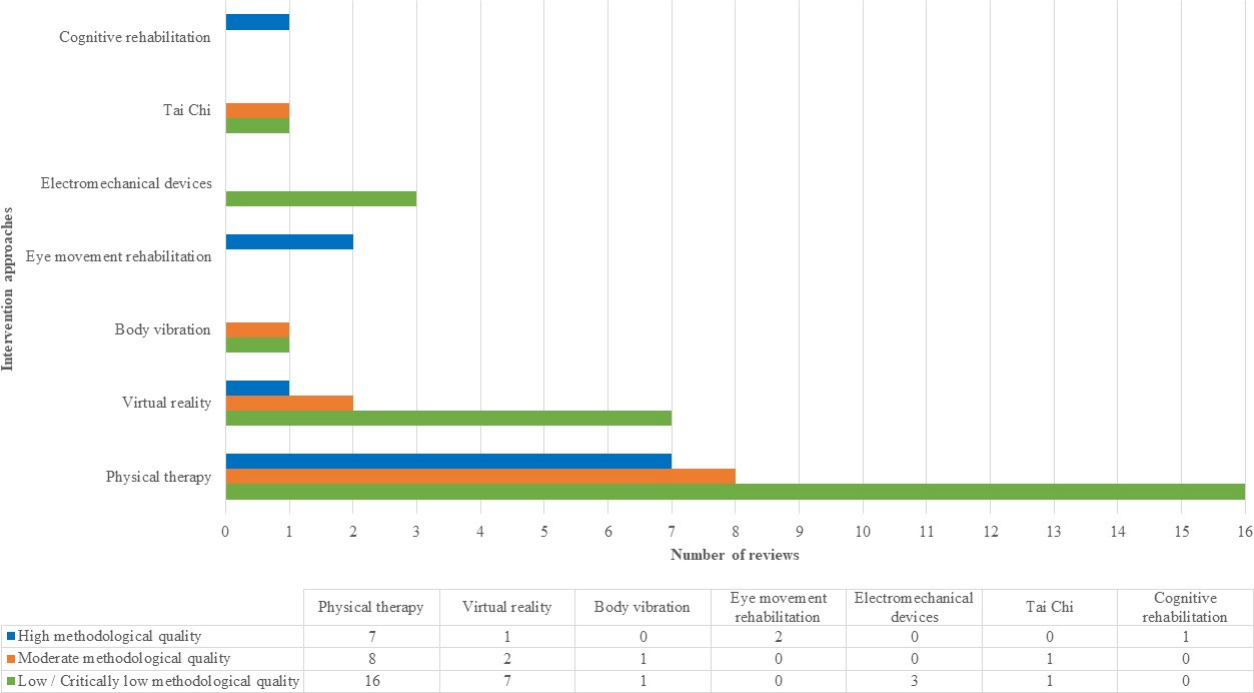
Methodological quality of each rehabilitation approach.

The rehabilitation interventions that may be considered effectiveness on balance are reported in **Table 3** and the methodological quality of each rehabilitation approach is showed in **Fig 2.**

**Table 3:** Effectiveness of rehabilitation interventions on balance.

Systematic Reviews	Interventions Classification	Outcome	Intervention and comparison	Relative effect	Quality of evidence (GRADE)
Saunders 2016	Physical therapy	BBS	Mixed training vs Control intervention	Statistically significant benefit of intervention	High
Saunders 2016	Physical therapy	Balance (BBS, FRT, Four Square Step Test, TBT)	Mixed training vs Control intervention	Statistically significant benefit of intervention	High
Pollock 2014	Physical therapy	BBS	Intervention vs attention control or usual care	Statistically significant benefit of intervention	Moderate
Vloothuis 2016	Physical therapy	BBS	Caregiver-mediated exercise as only intervention (CME)-core vs Usual care	Statistically significant benefit of intervention	Moderate
English 2017	Physical therapy	TUGT	Circuit class therapy vs Other interventions	Statistically significant benefit of intervention	Low
French 2016	Physical therapy	Sitting balance/reach	Repetitive task training vs Attention control, usual care	Statistically significant benefit of intervention	Low
Vloothuis 2016	Physical therapy	BBS	Caregiver-mediated exercise in addition to usual care or instead of usual care vs Usual care	Statistically significant benefit of intervention	Low
Vloothuis 2016	Physical therapy	PASS	Caregiver-mediated exercise in addition to usual care or instead of usual care vs Usual care	NO statistically significant differences between groups	Low
Vloothuis 2016	Physical therapy	Balance (BBS, PASS)	Caregiver-mediated exercise in addition to usual care or instead of usual care vs Usual care	Statistically significant benefit of intervention	Low
Pollock 2014	Physical therapy	BBS	Intervention vs no treatment	NO statistically significant differences between groups	Very low
Pollock 2014	Physical therapy	BBS	Intervention vs no treatment	NO statistically significant differences between groups	Very low
Vloothuis 2016	Physical therapy	BBS	Caregiver-mediated exercise in addition to usual care or instead of usual care vs Usual care	NO statistically significant differences between groups	Very low
Vloothuis 2016	Physical therapy	BBS	Caregiver-mediated exercise in addition to usual care or instead of usual care vs Usual care	NO statistically significant differences between groups	Very low
Lawrence 2017	Physical therapy	BBS	Yoga vs Waiting-list control	NO statistically significant differences between groups	Very low
Chen BL 2015	Tai Chi	BBS	Traditional Chinese exercises vs No intervention or other treatment	Statistically significant benefit of intervention	Very low

BBS = Berg Performance Scale; FRT = Functional Reach Test; PASS = Postural Assessment Scale for Stroke patients; TBT = Timed Balance Test; TUGT = Timed Up and Go Test

## Discussion

The aim of this overview of systematic reviews was to summarise evidence about the effectiveness of rehabilitation interventions for improving balance in stroke survivors.

This overview identified 248 primary studies, including more than 10,000 participants, combined within 51 SRs which contain evidence relevant to interventions to improve balance following stroke. However, there are very few SRs which are of high methodological quality (22%) and consequently this limits our ability to draw clear implications relating to the effectiveness of stroke rehabilitation interventions on balance. Those interventions for which there is evidence of a significant beneficial effect on balance include: exercise therapy, physical fitness training, care-mediated exercise in addition to usual care, repetitive task training, virtual reality and unstable support surface. Interventions for which there is no evidence of any significant benefit on balance include: yoga, water-based exercise, visual force platform feedback, lower limb reciprocal pedalling exercise, aerobic exercise, muscle strengthening, sitting and standing balance training, treadmill training, lumbar stabilization exercises, trunk training exercise, sling exercise training, exercise balance training, motor re-learning program therapy, cognitive motor therapy, Bobath technique, virtual therapy with Nintendo Wii and robot-assisted gait training have any effects. The evidence of traditional Chinese exercise, whole-body vibration training and circuit training effects is inconclusive.

This overview demonstrates that, while there is limited evidence showing benefit of some interventions, the quality of the evidence is insufficient to support firm conclusions relating to the effectiveness of most balance rehabilitation interventions. The inability to draw conclusions relating to the effectiveness of balance rehabilitation is largely dependent on the methodological quality of the available evidence. This overview has highlighted several important methodological considerations which have impacted on the ability to draw conclusions from the current evidence base. There is an urgent need for improved methodological quality in order to generate evidence which can support clinical decisions relating to balance rehabilitation for stroke survivors.

Evidence from this overview demonstrates that some of the main methodological issues which need addressed in order to improve the evidence base for rehabilitation include: 1. the heterogeneity of primary studies; 2. assessment and interpretation of risk of bias (RoB) of primary studies; 3. absence of systematic assessment and interpretation of the overall quality of the evidence combined within a SR.

First, there was generally clinical heterogeneity across studies included within SRs, in terms of participants (type and phase of stroke), interventions (dose, intensity and frequency) and balance outcomes. Results from heterogeneous studies were often pooled within meta-analyses, impacting on the results and conclusions of SRs. It is important that meta-analyses only combine the results of studies which have sufficient homogeneity [75]. Second, in rehabilitation research, the optimal approach to evaluation of RoB is still debated and which tool to use remains unclear. Often several criteria related to the design, conduct and analysis of trials are aggregated into a unique scale and an overall score, despite the fact that there is widespread consensus that use of an overall score is not recommended [76–78]. Our overview found that most SRs (53%) used the Physiotherapy Evidence Database (PEDro) scale for the risk of bias assessment. While the PEDro scale is widely used [79,80], it does not contain items that are specific to the rehabilitation field, it has been suggested that the optimal approach to assessing RoB in trials of physical therapy is the Cochrane RoB tool, rather than using the summary score from the PEDro scale [76]. The use of the PEDro scale therefore created difficulty in our evaluation of the methodological quality of included SRs and our interpretation of their results [19]. The importance of incorporating RoB assessments in evidence synthesis is widely recognized and the quality of evidence involves consideration of within-study RoB (methodological quality), directness of evidence, heterogeneity, precision of effect estimates and risk of publication bias [77]. It would be good practice to evaluate the influence of RoB on treatment effect and to interpret the results on this [81], but in our overview, only 25% interpreted the results taking into account the RoB assessment and mainly they described the results as ‘not statistically significant’ or ‘statistically significant’ without evaluating the quality of evidence.

Other methodological issues which may influence the quality of evidence of SRs include [82]: the number of participants, which are often are not enough to evaluate the impact of treatment; the description of interventions, which is often not sufficiently detailed to allow replication; and heterogeneity of outcome measures which do not enable statistical pooling of data using meta-analysis.

The management of these issues is a challenge and how best to address these within an overview of SRs method is still not well defined in literature [14]. The lack of alignment between PICO elements of overview questions and the aim of systematic reviews, and the overlap between primary studies, which may contribute data to more than one SR, can lead to challenges in the overview process. Furthermore, discordant RoB assessments of primary studies included in SRs can lead to difficulties in interpretation of results [13,83]. Therefore, systematic evaluation of certainty of evidence arising from the overview is difficult to achieve for several different methodological reasons, including the lack of standard methods for overviews, the use of overall scores to summarise RoB assessment and the assessment of certainty of evidence. While the GRADE approach provides a systematic method for assessing the certainty of evidence, there remains uncertainty about the best way to implement this within overviews [84].

In conclusion, the evaluation of quality of evidence is central to our ability to use rehabilitation research to inform clinical practice. We found 51 SRs (248 primary studies, including more than 10,000 participants) which contain evidence relating to balance rehabilitation for stroke, but most of them were of low methodological quality, which limits the ability for this evidence to inform clinical decision making. There is a need for carefully planned SRs addressing research questions which are priorities of key stakeholders, and which are conducted according to the highest possible methodological standards, in order to inform clinical practice and support optimal patient outcomes.

### Study limitations

The overview has some limitations. While it could be expected that there would be some overlap of primary articles within included SRs, we have not systematically explored these overlaps. Consequently, this may lead to inaccuracies in the reporting of data such as the numbers of participants and primary studies and may contribute to “double counting” of data within reported meta-analyses. Our findings–including assessments of certainty of evidence—are based on the information provided by the authors of the reviews, and we have not retrieved or evaluated data from any primary studies. Furthermore, we have grouped evidence according to different types of interventions, based on the terminology and descriptions provided within the SR; there may be considerable variations in the definitions used within individual SRs (for example, ‘physical therapy’), and this may have led to some inaccuracies in our categorisation of SRs. However, we used this approach to avoid introducing biases through our own interpretation of intervention descriptions. Our search is now out of date, as we conducted searches of databases up to December 2017. Running our searches from January 2018 to May 2019 identifies at least a further 7 other new SRs [85–91]. Exploration of these, by one reviewer, indicates that these would not change the conclusions within this overview. The issue of rapidly growing numbers of SRs highlights the challenges that healthcare decision makers and researchers face in relation to keeping up to date with evidence. Our overview finds that often there are large numbers of low quality reviews; to aid the evidence-based practice there is an urgent need for fewer high quality reviews, which do not overlap and which are maintained up to date.

## Conclusion

There are 51 SRs of evidence relating to the effectiveness of interventions to improve balance in people with stroke, but the majority of these are of poor methodological quality, limiting our ability to draw clear implications. Only 22% of these SRs were judged to be of high quality, highlighting the need to address important methodological issues within rehabilitation research. SRs summarised within this overview do provide some limited evidence that rehabilitation interventions, including exercise therapy, repetitive task training, physical fitness training, care-mediated exercise, virtual therapy and use of unstable support surfaces, may be beneficial for people with balance impairment after stroke, but further research is necessary to be confident in this finding.

## Supporting information

S1 Table Search strategy. (DOCX)

S2 Table Type of intervention. (DOCX)

S3 Table List of reviews, interventions and trials that contributed to the overview. (DOCX)

S4 Table Characteristics of included reviews. (DOCX)

S5 Table Overview of reviews. (DOCX)

S6 Table Prisma 2009 checklist. (DOC)

## References

[1] Winstein CJ, Stein J, Arena R, Bates B, Cherney LR, Cramer SC, et al Guidelines for Adult Stroke Rehabilitation and Recovery: A Guideline for Healthcare Professionals From the American Heart Association/American Stroke Association. Stroke. 2016;47: e98–e169. 10.1161/STR.0000000000000098 27145936

[2] Fisher A, Martin J, Srikusalanukul W, Davis M. Trends in stroke survival incidence rates in older Australians in the new millennium and forecasts into the future. J Stroke Cerebrovasc Dis Off J Natl Stroke Assoc. 2014;23: 759–770. 10.1016/j.jstrokecerebrovasdis.2013.06.035 23928347

[3] Stroke Association. State of the nation, Stroke statistics, February 2018. Available: https://www.stroke.org.uk/system/files/sotn_2018.pdf

[4] Feigin VL, Forouzanfar MH, Krishnamurthi R, Mensah GA, Connor M, Bennett DA, et al Global and regional burden of stroke during 1990–2010: findings from the Global Burden of Disease Study 2010. Lancet Lond Engl. 2014;383: 245–254. 10.1016/s0140-6736(13)61953-4 PMC4181600 24449944

[5] Ovbiagele B, Nguyen-Huynh MN. Stroke epidemiology: advancing our understanding of disease mechanism and therapy. Neurother J Am Soc Exp Neurother. 2011;8: 319–329. 10.1007/s13311-011-0053-1 21691873 PMC3250269

[6] Walker MF, Hoffmann TC, Brady MC, Dean CM, Eng JJ, Farrin AJ, et al Improving the Development, Monitoring and Reporting of Stroke Rehabilitation Research: Consensus-Based Core Recommendations from the Stroke Recovery and Rehabilitation Roundtable. Neurorehabil Neural Repair. 2017;31: 877–884. 10.1177/1545968317732686 29233072

[7] Corbetta D, Imeri F, Gatti R. Rehabilitation that incorporates virtual reality is more effective than standard rehabilitation for improving walking speed, balance and mobility after stroke: a systematic review. J Physiother. 2015;61: 117–124. 10.1016/j.jphys.2015.05.017 26093805

[8] Beyaert C, Vasa R, Frykberg GE. Gait post-stroke: Pathophysiology and rehabilitation strategies. Neurophysiol Clin Clin Neurophysiol. 2015;45: 335–355. 10.1016/j.neucli.2015.09.005 26547547

[9] Wonsetler EC, Bowden MG. A systematic review of mechanisms of gait speed change post-stroke. Part 2: exercise capacity, muscle activation, kinetics, and kinematics. Top Stroke Rehabil. 2017;24: 394–403. 10.1080/10749357.2017.1282413 28218021 PMC5702549

[10] Boehm WL, Gruben KG. Post-Stroke Walking Behaviors Consistent with Altered Ground Reaction Force Direction Control Advise New Approaches to Research and Therapy. Transl Stroke Res. 2016;7: 3–11. 10.1007/s12975-015-0435-5 26639659

[11] Pin-Barre C, Laurin J. Physical Exercise as a Diagnostic, Rehabilitation, and Preventive Tool: Influence on Neuroplasticity and Motor Recovery after Stroke. Neural Plast. 2015;2015: 608581 10.1155/2015/608581 26682073 PMC4670869

[12] Pollock A, Baer G, Pomeroy V, Langhorne P. Physiotherapy treatment approaches for the recovery of postural control and lower limb function following stroke. Cochrane Database Syst Rev. 2007; CD001920 10.1002/14651858.CD001920.pub2 17253468

[13] Lunny C, Brennan SE, McDonald S, McKenzie JE. Toward a comprehensive evidence map of overview of systematic review methods: paper 1-purpose, eligibility, search and data extraction. Syst Rev. 2017;6: 231 10.1186/s13643-017-0617-1 29162130 PMC5698938

[14] Hunt H, Pollock A, Campbell P, Estcourt L, Brunton G. An introduction to overviews of reviews: planning a relevant research question and objective for an overview. Syst Rev. 2018;7: 39 10.1186/s13643-018-0695-8 29490699 PMC5831229

[15] Pollock M, Fernandes R, Becker L, Pieper D, Hartling L. Chapter V: Overviews of Reviews. Draft version (8 October 2018) In: Higgins JPT, Thomas J, Chandler J, Cumpston MS, Li T, Page MJ, Welch V (editors). Cochrane Handbook for Systematic Reviews of Interventions. London: Cochrane.

[16] Moher D, Liberati A, Tetzlaff J, Altman DG, PRISMA Group. Preferred reporting items for systematic reviews and meta-analyses: the PRISMA statement. PLoS Med. 2009;6: e1000097 10.1371/journal.pmed.1000097 19621072 PMC2707599

[17] Green S, Higgins J, Alderson P, Clarke M, Mulrow C. Chapter 1: What is a systematic review? [updated February 2008]. In: Higgins JPT, Green S, editors. Cochrane handbook for systematic reviews of interventions version 5.0.0. The Cochrane Collaboration 2008 Available: http://www.cochrane-handbook.org/. Accessed 26 May 2009.

[18] Petró B, Papachatzopoulou A, Kiss RM. Devices and tasks involved in the objective assessment of standing dynamic balancing—A systematic literature review. PloS One. 2017;12: e0185188 10.1371/journal.pone.0185188 28934308 PMC5608356

[19] Shea BJ, Reeves BC, Wells G, Thuku M, Hamel C, Moran J, et al AMSTAR 2: a critical appraisal tool for systematic reviews that include randomised or non-randomised studies of healthcare interventions, or both. BMJ. 2017;358: j4008 10.1136/bmj.j4008 28935701 PMC5833365

[20] Whiting P, Savović J, Higgins JPT, Caldwell DM, Reeves BC, Shea B, et al ROBIS: A new tool to assess risk of bias in systematic reviews was developed. J Clin Epidemiol. 2016;69: 225–234. 10.1016/j.jclinepi.2015.06.005 26092286 PMC4687950

[21] Banzi R, Cinquini M, Gonzalez-Lorenzo M, Pecoraro V, Capobussi M, Minozzi S. Quality assessment versus risk of bias in systematic reviews: AMSTAR and ROBIS had similar reliability but differed in their construct and applicability. J Clin Epidemiol. 2018;99: 24–32. 10.1016/j.jclinepi.2018.02.024 29526556

[22] Pieper D, Puljak L, González-Lorenzo M, Minozzi S. Minor differences were found between AMSTAR 2 and ROBIS in the assessment of systematic reviews including both randomized and nonrandomized studies. J Clin Epidemiol. 2019;108: 26–33. 10.1016/j.jclinepi.2018.12.004 30543911

[23] World Confederation for Physical Therapy Policy statement: Description of physical therapy London, UK: WCPT; 2007 Available: www.wcpt.org/policy/ps-descriptionPT. Accessed 10 Mar 2017.

[24] Pollock A, Baer G, Campbell P, Choo PL, Forster A, Morris J, et al Physical rehabilitation approaches for the recovery of function and mobility following stroke. Cochrane Database Syst Rev. 2014; CD001920 10.1002/14651858.CD001920.pub3 24756870 PMC6465059

[25] Saunders DH, Sanderson M, Hayes S, Kilrane M, Greig CA, Brazzelli M, et al Physical fitness training for stroke patients. Cochrane Database Syst Rev. 2016;3: CD003316 10.1002/14651858.CD003316.pub6 27010219 PMC6464717

[26] French B, Thomas LH, Coupe J, McMahon NE, Connell L, Harrison J, et al Repetitive task training for improving functional ability after stroke. Cochrane Database Syst Rev. 2016;11: CD006073 10.1002/14651858.CD006073.pub3 27841442 PMC6464929

[27] Vloothuis JD, Mulder M, Veerbeek JM, Konijnenbelt M, Visser-Meily JM, Ket JC, et al Caregiver-mediated exercises for improving outcomes after stroke. Cochrane Database Syst Rev. 2016;12: CD011058 10.1002/14651858.CD011058.pub2 28002636 PMC6463929

[28] Lawrence M, Celestino Junior FT, Matozinho HH, Govan L, Booth J, Beecher J. Yoga for stroke rehabilitation. Cochrane Database Syst Rev. 2017;12: CD011483 10.1002/14651858.CD011483.pub2 29220541 PMC6486003

[29] Mehrholz J, Kugler J, Pohl M. Water-based exercises for improving activities of daily living after stroke. Cochrane Database Syst Rev. 2011; CD008186 10.1002/14651858.CD008186.pub2 21249701 PMC6464732

[30] English C, Hillier SL, Lynch EA. Circuit class therapy for improving mobility after stroke. Cochrane Database Syst Rev. 2017;6: CD007513 10.1002/14651858.CD007513.pub3 28573757 PMC6481475

[31] Guyatt G, Oxman AD, Akl EA, Kunz R, Vist G, Brozek J, et al GRADE guidelines: 1. Introduction-GRADE evidence profiles and summary of findings tables. J Clin Epidemiol. 2011;64: 383–394. 10.1016/j.jclinepi.2010.04.026 21195583

[32] Pollock A, Hazelton C, Henderson CA, Angilley J, Dhillon B, Langhorne P, et al Interventions for disorders of eye movement in patients with stroke. Cochrane Database Syst Rev. 2011; CD008389 10.1002/14651858.CD008389.pub2 21975780 PMC13381072

[33] Pollock A, Hazelton C, Henderson CA, Angilley J, Dhillon B, Langhorne P, et al Interventions for visual field defects in patients with stroke. Cochrane Database Syst Rev. 2011; CD008388 10.1002/14651858.CD008388.pub2 21975779

[34] Laver KE, Lange B, George S, Deutsch JE, Saposnik G, Crotty M. Virtual reality for stroke rehabilitation. Cochrane Database Syst Rev. 2017;11: CD008349 10.1002/14651858.CD008349.pub4 29156493 PMC6485957

[35] Bowen A, Hazelton C, Pollock A, Lincoln NB. Cognitive rehabilitation for spatial neglect following stroke. Cochrane Database Syst Rev. 2013; CD003586 10.1002/14651858.CD003586.pub3 23813503 PMC6464849

[36] van Duijnhoven HJR, Heeren A, Peters MAM, Veerbeek JM, Kwakkel G, Geurts ACH, et al Effects of Exercise Therapy on Balance Capacity in Chronic Stroke: Systematic Review and Meta-Analysis. Stroke. 2016;47: 2603–2610. 10.1161/STROKEAHA.116.013839 27633021

[37] Langhorne P, Coupar F, Pollock A. Motor recovery after stroke: a systematic review. Lancet Neurol. 2009;8: 741–754. 10.1016/S1474-4422(09)70150-4 19608100

[38] Tyson SF, Kent RM. Effects of an ankle-foot orthosis on balance and walking after stroke: a systematic review and pooled meta-analysis. Arch Phys Med Rehabil. 2013;94: 1377–1385. 10.1016/j.apmr.2012.12.025 23416220

[39] Stoller O, de Bruin ED, Knols RH, Hunt KJ. Effects of cardiovascular exercise early after stroke: systematic review and meta-analysis. BMC Neurol. 2012;12: 45 10.1186/1471-2377-12-45 22727172 PMC3495034

[40] Veerbeek JM, van Wegen E, van Peppen R, van der Wees PJ, Hendriks E, Rietberg M, et al What is the evidence for physical therapy poststroke? A systematic review and meta-analysis. PloS One. 2014;9: e87987 10.1371/journal.pone.0087987 24505342 PMC3913786

[41] Van Criekinge T, Saeys W, Vereeck L, De Hertogh W, Truijen S. Are unstable support surfaces superior to stable support surfaces during trunk rehabilitation after stroke? A systematic review. Disabil Rehabil. 2018;40: 1981–1988. 10.1080/09638288.2017.1323030 28482696

[42] Barclay-Goddard R, Stevenson T, Poluha W, Moffatt MEK, Taback SP. Force platform feedback for standing balance training after stroke. Cochrane Database Syst Rev. 2004; CD004129 10.1002/14651858.CD004129.pub2 15495079 PMC6464938

[43] Bonini-Rocha AC, de Andrade ALS, Moraes AM, Gomide Matheus LB, Diniz LR, Martins WR. Effectiveness of Circuit-Based Exercises on Gait Speed, Balance, and Functional Mobility in People Affected by Stroke: A Meta-Analysis. PM R. 2018;10: 398–409. 10.1016/j.pmrj.2017.09.014 29111465

[44] de Rooij IJM, van de Port IGL, Meijer J-WG. Effect of Virtual Reality Training on Balance and Gait Ability in Patients With Stroke: Systematic Review and Meta-Analysis. Phys Ther. 2016;96: 1905–1918. 10.2522/ptj.20160054 27174255

[45] Li Z, Han X-G, Sheng J, Ma S-J. Virtual reality for improving balance in patients after stroke: A systematic review and meta-analysis. Clin Rehabil. 2016;30: 432–440. 10.1177/0269215515593611 26141808

[46] Chen B-L, Guo J-B, Liu M-S, Li X, Zou J, Chen X, et al Effect of Traditional Chinese Exercise on Gait and Balance for Stroke: A Systematic Review and Meta-Analysis. PloS One. 2015;10: e0135932 10.1371/journal.pone.0135932 26291978 PMC4546302

[47] Yang X, Wang P, Liu C, He C, Reinhardt JD. The effect of whole body vibration on balance, gait performance and mobility in people with stroke: a systematic review and meta-analysis. Clin Rehabil. 2015;29: 627–638. 10.1177/0269215514552829 25311142

[48] Sorinola IO, Powis I, White CM. Does additional exercise improve trunk function recovery in stroke patients? A meta-analysis. NeuroRehabilitation. 2014;35: 205–213. 10.3233/NRE-141123 24990030

[49] Tang A, Tao A, Soh M, Tam C, Tan H, Thompson J, et al The effect of interventions on balance self-efficacy in the stroke population: a systematic review and meta-analysis. Clin Rehabil. 2015;29: 1168–1177. 10.1177/0269215515570380 25681409 PMC4596690

[50] Hancock NJ, Shepstone L, Winterbotham W, Pomeroy V. Effects of lower limb reciprocal pedalling exercise on motor function after stroke: a systematic review of randomized and nonrandomized studies. Int J Stroke Off J Int Stroke Soc. 2012;7: 47–60. 10.1111/j.1747-4949.2011.00728.x 22111955

[51] An M, Shaughnessy M. The effects of exercise-based rehabilitation on balance and gait for stroke patients: a systematic review. J Neurosci Nurs J Am Assoc Neurosci Nurses. 2011;43: 298–307. 10.1097/JNN.0b013e318234ea24 22089406

[52] Wist S, Clivaz J, Sattelmayer M. Muscle strengthening for hemiparesis after stroke: A meta-analysis. Ann Phys Rehabil Med. 2016;59: 114–124. 10.1016/j.rehab.2016.02.001 26969343

[53] Van Peppen RPS, Kwakkel G, Wood-Dauphinee S, Hendriks HJM, Van der Wees PJ, Dekker J. The impact of physical therapy on functional outcomes after stroke: what’s the evidence? Clin Rehabil. 2004;18: 833–862. 10.1191/0269215504cr843oa 15609840

[54] Ko D-S, Jung D-I, Bae S-Y. Effect of lumbar stabilization exercises on the balance ability of patients with stroke: a systematic review. J Phys Ther Sci. 2014;26: 1993–1996. 10.1589/jpts.26.1993 25540515 PMC4273075

[55] Cabanas-Valdés R, Cuchi GU, Bagur-Calafat C. Trunk training exercises approaches for improving trunk performance and functional sitting balance in patients with stroke: a systematic review. NeuroRehabilitation. 2013;33: 575–592. 10.3233/NRE-130996 24018373

[56] Chen L, Chen J, Peng Q, Chen J, Zou Y, Liu G. Effect of Sling Exercise Training on Balance in Patients with Stroke: A Meta-Analysis. PloS One. 2016;11: e0163351 10.1371/journal.pone.0163351 27727288 PMC5058486

[57] Lubetzky-Vilnai A, Kartin D. The effect of balance training on balance performance in individuals poststroke: a systematic review. J Neurol Phys Ther JNPT. 2010;34: 127–137. 10.1097/NPT.0b013e3181ef764d 20716987

[58] Bank J, Charles K, Morgan P. What is the effect of additional physiotherapy on sitting balance following stroke compared to standard physiotherapy treatment: a systematic review. Top Stroke Rehabil. 2016;23: 15–25. 10.1179/1945511915Y.0000000005 26086177

[59] Hammer A, Nilsagård Y, Wallquist M. Balance training in stroke patients–a systematic review of randomized, controlled trials. Adv Physiother. 2008;10: 163–172. 10.1080/14038190701757656

[60] Kollen BJ, Lennon S, Lyons B, Wheatley-Smith L, Scheper M, Buurke JH, et al The effectiveness of the Bobath concept in stroke rehabilitation: what is the evidence? Stroke. 2009;40: e89–97. 10.1161/STROKEAHA.108.533828 19182079

[61] Wang X-Q, Pi Y-L, Chen B-L, Chen P-J, Liu Y, Wang R, et al Cognitive motor interference for gait and balance in stroke: a systematic review and meta-analysis. Eur J Neurol. 2015;22: 555–e37. 10.1111/ene.12616 25560629 PMC4342759

[62] Van Peppen RPS, Kortsmit M, Lindeman E, Kwakkel G. Effects of visual feedback therapy on postural control in bilateral standing after stroke: a systematic review. J Rehabil Med. 2006;38: 3–9. 16548079 10.1080/16501970500344902

[63] Wevers L, van de Port I, Vermue M, Mead G, Kwakkel G. Effects of task-oriented circuit class training on walking competency after stroke: a systematic review. Stroke. 2009;40: 2450–2459. 10.1161/STROKEAHA.108.541946 19461035

[64] Chen L, Lo WLA, Mao YR, Ding MH, Lin Q, Li H, et al Effect of Virtual Reality on Postural and Balance Control in Patients with Stroke: A Systematic Literature Review. BioMed Res Int. 2016;2016: 7309272 10.1155/2016/7309272 28053988 PMC5174165

[65] Luque-Moreno C, Ferragut-Garcías A, Rodríguez-Blanco C, Heredia-Rizo AM, Oliva-Pascual-Vaca J, Kiper P, et al A Decade of Progress Using Virtual Reality for Poststroke Lower Extremity Rehabilitation: Systematic Review of the Intervention Methods. BioMed Res Int. 2015;2015: 342529 10.1155/2015/342529 26539480 PMC4619784

[66] Cheok G, Tan D, Low A, Hewitt J. Is Nintendo Wii an Effective Intervention for Individuals With Stroke? A Systematic Review and Meta-Analysis. J Am Med Dir Assoc. 2015;16: 923–932. 10.1016/j.jamda.2015.06.010 26253322

[67] Dos Santos LRA, Carregosa AA, Masruha MR, Dos Santos PA, Da Silveira Coêlho ML, Ferraz DD, et al The Use of Nintendo Wii in the Rehabilitation of Poststroke Patients: A Systematic Review. J Stroke Cerebrovasc Dis Off J Natl Stroke Assoc. 2015;24: 2298–2305. 10.1016/j.jstrokecerebrovasdis.2015.06.010 26303792

[68] Iruthayarajah J, McIntyre A, Cotoi A, Macaluso S, Teasell R. The use of virtual reality for balance among individuals with chronic stroke: a systematic review and meta-analysis. Top Stroke Rehabil. 2017;24: 68–79. 10.1080/10749357.2016.1192361 27309680

[69] Chen J, Jin W, Zhang X-X, Xu W, Liu X-N, Ren C-C. Telerehabilitation Approaches for Stroke Patients: Systematic Review and Meta-analysis of Randomized Controlled Trials. J Stroke Cerebrovasc Dis Off J Natl Stroke Assoc. 2015;24: 2660–2668. 10.1016/j.jstrokecerebrovasdis.2015.09.014 26483155

[70] Tally Z, Boetefuer L, Kauk C, Perez G, Schrand L, Hoder J. The efficacy of treadmill training on balance dysfunction in individuals with chronic stroke: a systematic review. Top Stroke Rehabil. 2017;24: 539–546. 10.1080/10749357.2017.1345445 28687056

[71] Swinnen E, Beckwée D, Meeusen R, Baeyens J-P, Kerckhofs E. Does robot-assisted gait rehabilitation improve balance in stroke patients? A systematic review. Top Stroke Rehabil. 2014;21: 87–100. 10.1310/tsr2102-87 24710969

[72] Lin S, Sun Q, Wang H, Xie G. Influence of transcutaneous electrical nerve stimulation on spasticity, balance, and walking speed in stroke patients: A systematic review and meta-analysis. J Rehabil Med. 2018;50: 3–7. 10.2340/16501977-2266 28862711

[73] Lu J, Xu G, Wang Y. Effects of whole body vibration training on people with chronic stroke: a systematic review and meta-analysis. Top Stroke Rehabil. 2015;22: 161–168. 10.1179/1074935714Z.0000000005 26084320

[74] Ge L, Zheng Q-X, Liao Y-T, Tan J-Y, Xie Q-L, Rask M. Effects of traditional Chinese exercises on the rehabilitation of limb function among stroke patients: A systematic review and meta-analysis. Complement Ther Clin Pract. 2017;29: 35–47. 10.1016/j.ctcp.2017.08.005 29122267

[75] Murad MH, Montori VM, Ioannidis JPA, Jaeschke R, Devereaux PJ, Prasad K, et al How to read a systematic review and meta-analysis and apply the results to patient care: users’ guides to the medical literature. JAMA. 2014;312: 171–179. 10.1001/jama.2014.5559 25005654

[76] Armijo-Olivo S, da Costa BR, Cummings GG, Ha C, Fuentes J, Saltaji H, et al PEDro or Cochrane to Assess the Quality of Clinical Trials? A Meta-Epidemiological Study. PloS One. 2015;10: e0132634 10.1371/journal.pone.0132634 26161653 PMC4498768

[77] Olivo SA, Macedo LG, Gadotti IC, Fuentes J, Stanton T, Magee DJ. Scales to assess the quality of randomized controlled trials: a systematic review. Phys Ther. 2008;88: 156–175. 10.2522/ptj.20070147 18073267

[78] Higgins J, Altman D. Chapter 8: Assessing risk of bias in included studies In: Higgins J, Green S, editors. Cochrane Handbook for Systematic Reviews of Interventions version 50. Chichester, UK: John Wiley & Sons, Ltd 2008.

[79] Herbert R, Moseley A, Sherrington C. PEDro: a database of randomised controlled trials in physiotherapy. Health Inf Manag J Health Inf Manag Assoc Aust. 1998;28: 186–188. 10.1177/183335839902800410 10387366

[80] da Costa BR, Hilfiker R, Egger M. PEDro’s bias: summary quality scores should not be used in meta-analysis. J Clin Epidemiol. 2013;66: 75–77. 10.1016/j.jclinepi.2012.08.003 23177896

[81] Armijo-Olivo S, Fuentes J, Ospina M, Saltaji H, Hartling L. Inconsistency in the items included in tools used in general health research and physical therapy to evaluate the methodological quality of randomized controlled trials: a descriptive analysis. BMC Med Res Methodol. 2013;13: 116 10.1186/1471-2288-13-116 24044807 PMC3848693

[82] Gillespie DC, Bowen A, Chung CS, Cockburn J, Knapp P, Pollock A. Rehabilitation for post-stroke cognitive impairment: an overview of recommendations arising from systematic reviews of current evidence. Clin Rehabil. 2015;29: 120–128. 10.1177/0269215514538982 24942480

[83] Lunny C, Brennan SE, McDonald S, McKenzie JE. Toward a comprehensive evidence map of overview of systematic review methods: paper 2-risk of bias assessment; synthesis, presentation and summary of the findings; and assessment of the certainty of the evidence. Syst Rev. 2018;7: 159 10.1186/s13643-018-0784-8 30314530 PMC6186052

[84] Lunny C, Brennan SE, McDonald S, McKenzie JE. Evidence map of studies evaluating methods for conducting, interpreting and reporting overviews of systematic reviews of interventions: rationale and design. Syst Rev. 2016;5: 4 10.1186/s13643-015-0178-0 26739283 PMC4702312

[85] Wu S, Chen J, Wang S, Jiang M, Wang X, Wen Y. Effect of Tai Chi Exercise on Balance Function of Stroke Patients: A Meta-Analysis. Med Sci Monit Basic Res. 2018;24: 210–215. 10.12659/MSMBR.911951 30504762 PMC6289026

[86] Louie DR, Lim SB, Eng JJ. The Efficacy of Lower Extremity Mirror Therapy for Improving Balance, Gait, and Motor Function Poststroke: A Systematic Review and Meta-Analysis. J Stroke Cerebrovasc Dis Off J Natl Stroke Assoc. 2019;28: 107–120. 10.1016/j.jstrokecerebrovasdis.2018.09.017 30314760

[87] Zou L, Yeung A, Li C, Chiou S-Y, Zeng N, Tzeng H-M, et al Effects of Mind−Body Movements on Balance Function in Stroke Survivors: A Meta-Analysis of Randomized Controlled Trials. Int J Environ Res Public Health. 2018;15 10.3390/ijerph15061292 29925770 PMC6025433

[88] Broderick P, Horgan F, Blake C, Ehrensberger M, Simpson D, Monaghan K. Mirror therapy for improving lower limb motor function and mobility after stroke: A systematic review and meta-analysis. Gait Posture. 2018;63: 208–220. 10.1016/j.gaitpost.2018.05.017 29775908

[89] Li GY, Wang W, Liu GL, Zhang Y. Effects of Tai Chi on balance and gait in stroke survivors: A systematic meta-analysis of randomized controlled trials. J Rehabil Med. 2018;50: 582–588. 10.2340/16501977-2346 29736553

[90] Schröder J, Truijen S, Van Criekinge T, Saeys W. Peripheral somatosensory stimulation and postural recovery after stroke—a systematic review. Top Stroke Rehabil. 2018;25: 312–320. 10.1080/10749357.2018.1440694 29473456

[91] Pollock A, Hazelton C, Rowe FJ, Jonuscheit S, Kernohan A, Angilley J, et al Interventions for visual field defects in people with stroke. Cochrane Database Syst Rev. 2019;5: CD008388 10.1002/14651858.CD008388.pub3 31120142 PMC6532331

