# Supplementary material for: Rehabilitation interventions for improving balance following stroke: An overview of systematic reviews
Source: PLoS One. 2019 Jul 19;14(7):e0219781. doi: 10.1371/journal.pone.0219781 (PMC6641159; doi:10.1371/journal.pone.0219781)
Supplement: S1 Table — (DOCX) [file pone.0219781.s001.docx]

| Supplementary Table 1. Search strategy | |
| --- | --- |
| Database | **Search strategy** |
| MEDLINE  (Pubmed) | "stroke rehabilitation"[MeSH Terms] AND "postural balance"[MeSH Terms] AND ((systematic[sb] OR Meta-Analysis[ptyp]) AND "humans"[MeSH Terms]) |
| Campbell Systematic Reviews http://www.campbellcollaboration.org/lib/?go=monograph) | Balance rehabilitation stroke [Title]  Balance [Keyword]  Rehabilitation [Keyword] |
| Database of Abstracts of reviews of Effects (https://www.ncbi.nlm.nih.gov/pubmedhealth/about/DARE/) | “Balance” [All fields] AND “rehabilitation” [All fields] AND “stroke” [All fields]  “postural control” “rehabilitation” AND “systematic review” |
| Epistemonikos  (www.epistemonikos.org) | 1.(title (balance) OR abstract (balance)) AND (title (rehabilitation) OR abstract(rehabilitation))AND (title (stroke) OR abstract(stroke)) + filter “systematic review”.  2. (title (balance) OR abstract (balance)) AND (title (rehabilitation) OR abstract(rehabilitation)) + filter “systematic review”. |
| JBI Database of Systematic Reviews and Implementation Reports (http://joannabriggslibrary.org/index.php/jbisrir) | Balance[title/abstract] rehabilitation [title/abstract] stoke [title/abstract] + filter Articles. |
| The Community Guide (http://www.thecommunityguide.org/index.html). | Balance rehabilitation stroke + filter “Systematic reviews” |
| Evidence for Policy and Practice Information and co-ordinating Centre (http://eppi.ioe.ac.uk/cms/) | Balance rehabilitation stroke + filter “Articles” |
| NICE (National Institute for health and Care Excellence) (www.nice.org.uk) | “balance” “rehabilitation” “stroke”. |
| Agency for Healthcare Research and Quality  (www.ahrq.gov) | “balance rehabilitation stroke” [All Fields]+ pages written in English + without word “drug” and “pharmacological” |
| Cochrane Library  (www.cochranelibrary.com) | 1. ”balance rehabilitation stroke” [Title/Abstract/Keyword] + filter “systematic review”  2. ”balance rehabilitation” ” [Title/Abstract/Keyword] + filter “review” |
| EMBASE  (https://www.elsevier.com/solutions/embase-biomedical-research) | (“balance”/exp OR “balance”) AND (“rehabilitation”/ exp OR “rehabilitation”) AND (“stroke”/ exp OR “stroke”)+ Disease: cerebrovascular accident + Study types: “human”, “systematic review”  Floating subheadings: rehabilitation. |

| Supplementary table 1. (Continued) | |
| --- | --- |
| Database | **Search strategy** |
| CINAHL | (Title)Balance (abstract) balance OR (title)rehabilitation (abstract) rehabilitation OR (title) stroke (abstract) stroke. Limiters: full text. Main topic: rehabilitation, stroke. Language: English. |
| PsycINFO | (Title)Balance (abstract) balance OR (title)rehabilitation (abstract) rehabilitation OR (title) stroke (abstract) stroke. Topic: rehabilitation, cerebrovascular accidents. Language: English. Methodology: systematic review. |
