# Supplementary material for: Rehabilitation interventions for improving balance following stroke: An overview of systematic reviews
Source: PLoS One. 2019 Jul 19;14(7):e0219781. doi: 10.1371/journal.pone.0219781 (PMC6641159; doi:10.1371/journal.pone.0219781)
Supplement: S2 Table — (DOCX) [file pone.0219781.s002.docx]

| Supplementary Table 2. Type of intervention | | | |
| --- | --- | --- | --- |
| **Type of intervention** | **Total** | **CSRs** | **NCSRs** |
| **Physical therapy** | 61% (31) | 26% (8) | 74% (23) |
| Exercise | 35% (11) | 9% (1) | 91% (10) |
| Balance | 6% (2) | 0% (0) | 100% (2) |
| Trunk | 6% (2) | 0% (0) | 100% (2) |
| Pedalling | 3% (1) | 0% (0) | 100% (1) |
| Lumbar stabilization | 3% (1) | 0% (0) | 100% (1) |
| Bobath | 3% (1) | 0% (0) | 100% (1) |
| Cognitive | 3% (1) | 0% (0) | 100% (1) |
| Additional | 3% (1) | 0% (0) | 100% (1) |
| Physical fitness | 3% (1) | 100% (1) | 0% (0) |
| Yoga | 3% (1) | 100% (1) | 0% (0) |
| Care-giver | 3% (1) | 100% (1) | 0% (0) |
| Circuit training | 10% (3) | 33% (1) | 67% (2) |
| Surface | 3% (1) | 0% (0) | 100% (1) |
| Water-based | 3% (1) | 100% (1) | 0% (0) |
| Visual feedback | 6% (2) | 50% (1) | 50% (1) |
| Repetitive task training | 3% (1) | 100% (1) | 0% (0) |
| **Virtual reality** | 20% (10) | 10% (1) | 90% (9) |
| Nintendo | 20% (2) | 0% (0) | 100% (2) |
| Telerehabilitation | 10% (1) | 0% (0) | 100% (1) |
| VR | 70% (7) | 14% (1) | 86% (6) |
| **Body vibration** | 4% (2) | 0% (0) | 100% (2) |
| **Eye movement rehabilitation** | 4% (2) | 100% (2) | 0% (0) |
| **Electromechanical devices** | 6% (3) | 0% (0) | 100% (3) |
| Robot | 33% (1) | 0% (0) | 100% (1) |
| Treadmill | 33% (1) | 0% (0) | 100% (1) |
| TENS | 33% (1) | 0% (0) | 100% (1) |
| **Tai Chi** | 4% (2) | 0% (0) | 100% (2) |
| **Cognitive rehabilitation** | 2% (1) | 100% (1) | 0% (0) |

CSRs= Cochrane Systematic Reviews; NCSRs= Non-Cochrane Systematic Reviews
