# Supplementary material for: Rehabilitation interventions for improving balance following stroke: An overview of systematic reviews
Source: PLoS One. 2019 Jul 19;14(7):e0219781. doi: 10.1371/journal.pone.0219781 (PMC6641159; doi:10.1371/journal.pone.0219781)
Supplement: S3 Table — (DOCX) [file pone.0219781.s003.docx]

| Supplementary Table 3. List of reviews, interventions and trials that contributed to the overview | | | |
| --- | --- | --- | --- |
| **Intervention** | **Review** | **Unique trials contributed and participants (n) on balance and postural control** | **Study design** |
| ***Cochrane Reviews (CSRs)*** | | | |
| Circuit class therapy | English 2017 PMID: 28573757 | Blennerhassett 2004 (30) | RCT |
|  |  | Dean 2000 (9) | RCT |
|  |  | Dean 2012 (151) | RCT |
|  |  | Harrington 2010 (243) | RCT |
|  |  | Kim 2016 (20) | RCT |
|  |  | Marigold 2005 (48) | RCT |
|  |  | Marsden 2010 (26) | RCT |
|  |  | Moore 2015 (40) | RCT |
|  |  | Mudge 2009 (55) | RCT |
|  |  | Pang 2005 (63) | RCT |
|  |  | Van de Port 2012 (250) | RCT |
| Visual or auditory force platform feedback | Barclay-Goddard 2004 PMID: 15495079 | Chen 2002 (41) | RCT |
|  |  | Geiger 2001 (13) | RCT |
|  |  | Lee 1996 (60) | RCT |
|  |  | Sackley 1997 (25) | RCT |
|  |  | Shumway-Cook 1988 (16) | RCT |
|  |  | Walker 2000 (30) | RCT |
|  |  | Wong 1997 (60) | RCT |
| Physical rehabilitation | Pollock 2014 PMID: 24756870 | Brock 2005 (26) | RCT |
|  |  | Chan 2006 (66) | RCT |
|  |  | Duncan 1998 (20) | RCT |
|  |  | Duncan 2003 (92) | RCT |
|  |  | Holmgren 2006 (34) | RCT |
|  |  | Kim 2012 (20) | RCT |
|  |  | Richards 1993 (27) | RCT |
|  |  | Salbach 2004 (91) | RCT |
|  |  | Shin 2011 (21) | RCT |
|  |  | Wang 2005 (44) | RCT |
|  |  | Yelnik 2008 (68) | RCT |
| Repetitive task training | French 2016 PMID: 27841442 | de Sèze 2001 (20) | RCT |
|  |  | Dean 1997 (20) | RCT |
|  |  | Dean 2007 (12) | RCT |
|  |  | Holmgren 2010 (34) | RCT |
|  |  | Howe 2005 (35) | RCT |
|  |  | Kim 2012 (20) | RCT |
|  |  | Kim 2016 (20) | RCT |
|  |  | Langhammer 2000 (61) | RCT |
|  |  | McClellan 2004 (26) | RCT |
|  |  | Park 2011 (25) | RCT |
|  |  | Salbach 2004 (91) | RCT |
|  |  | Tung 2010 (32) | RCT |
|  |  | Van de Port 2012 (250) | RCT |
|  |  | VanVliet 2005 (120) | RCT |
| Caregiver-mediated exercise | Vloothuis 2016 PMID: 28002636 | Galvin 2011 (40) | RCT |
|  |  | Wang 2015 (51) | RCT |
|  |  | Dai 2013 (48) | RCT |
| Water-based exercises | Mehrholz 2011 PMID: 21249701 | Chu 2004 (13) | RCT |
|  |  | Noh 2008 (25) | RCT |
| Interventions for eye movement disorders | Pollock 2011 (a) PMID: 21975780 | N/A |  |

| Supplementary Table 3. (Continued) | | | | | | |
| --- | --- | --- | --- | --- | --- | --- |
| Intervention | | Review | Unique trials contributed and participants (n) on balance and postural control | | Study design | |
| Intervention specifically targeted at improving the visual field defect or improving the ability of the participant to cope with the visual field loss | Pollock 2011 (b) PMID: 21975779 | | | N/A | |  |
| Physical fitness training | Saunders 2016 PMID: 27010219 | | | Bateman 2001 (77) | | RCT |
|  |  |  |  | Duncan 1998 (20) | | RCT |
|  |  |  |  | Duncan 2003 (100) | | RCT |
|  |  |  |  | Galvin 2011 (40) | | RCT |
|  |  |  |  | Globas 2012 (36) | | RCT |
|  |  |  |  | Jin 2013 (128) | | RCT |
|  |  |  |  | Lee 2013 (33) | | RCT |
|  |  |  |  | MacKay-Lyons 2013 (50) | | RCT |
|  |  |  |  | Mead 2007 (66) | | RCT |
|  |  |  |  | Moore 2010 (20) | | RCT |
|  |  |  |  | Richards 1993 (17) | | RCT |
|  |  |  |  | Richards 2004 (62) | | RCT |
|  |  |  |  | Salbach 2004 (91) | | RCT |
|  |  |  |  | Shin 2011 (21) | | RCT |
|  |  |  |  | Son 2014 (28) | | RCT |
|  |  |  |  | Takami 2010 (36) | | RCT |
|  |  |  |  | Toledano-Zarhi 2011 (28) | | RCT |
|  |  |  |  | Van de Port 2012 (242) | | RCT |
|  |  |  |  | Verheyden 2009 (33) | | RCT |
| Yoga | Lawrence 2017 PMID: 29220541 | | | Immink 2014 (22) | | RCT |
|  |  |  |  | Schmid 2012 (47) | | RCT |
| Virtual reality | Laver 2017 PMID: 29156493 | | | Barcala 2013 (20) | | RCT |
|  |  |  |  | Bower 2015 (14) | | RCT |
|  |  |  |  | Chow 2013 (14) | | RCT |
|  |  |  |  | Han 2013 (12) | | RCT |
|  |  |  |  | Hung 2014 (28) | | RCT |
|  |  |  |  | Kim 2009 (24) | | RCT |
|  |  |  |  | Ko 2015 (52) | | RCT |
|  |  |  |  | Lee 2013 (22) | | RCT |
|  |  |  |  | Lee 2014 (24) | | RCT |
|  |  |  |  | Llorens 2015 (20) | | RCT |
|  |  |  |  | Low 2012 (20) | | RCT |
|  |  |  |  | Morone 2014 (50) | | RCT |
|  |  |  |  | Xiang 2014 (20) | | RCT |
| Cognitive rehabilitation | Bowen 2013 PMID: 23813503 | | | N/A | |  |
| ***Non-Cochrane Reviews (non-CSRs)*** | | | | | | |
| Whole body vibration | Lu 2015  PMID: 26084320 | | | Lau 2012 (82) | | RCT |
|  |  |  |  | Marin 2013 (20) | | RCT |
|  |  |  |  | Brogardh 2012 (31) | | RCT |
| Whole body vibration | Yang 2015 PMID: 25311142 | | | Brogardh 2012 (31) | | RCT |
|  |  |  |  | Lau 2012 (82) | | RCT |
|  |  |  |  | van Nes 2006 (53) | | RCT |
|  |  |  |  | Marin 2013 (20) | | RCT |
| Circuit-based exercise | Bonini-Rocha 2018  PMID: 29111465 | | | Kim 2012 (20) | | RCT |
|  |  |  |  | Pang 2005 (63) | | RCT |
|  |  |  |  | Salbach 2004 (91) | | RCT |
| Circuit class training | Wevers 2009 PMID: 19461035 | | | Dean 2000 (9) | | RCT |
|  |  |  |  | Blennerhassett 2004 (30) | | RCT |
|  |  |  |  | Pang 2005 (63) | | RCT |
|  |  |  |  | Salbach 2004 (91) | | RCT |
|  |  |  |  | Yang 2006 (48) | | RCT |
| Robot-assisted gait training | Swinnen 2014  PMID: 24710969 | | | Dias 2007 (40) | | RCT |
|  |  |  |  | Hidler 2009 (63) | | RCT |
|  |  |  |  | Peurala 2005 (45) | | RCT |
|  |  |  |  | Ng 2008 (53) | | RCT |
|  |  |  |  | Tong 2006 (50) | | RCT |
|  |  |  |  | Westlake 2009 (16) | | RCT |
|  |  |  |  | Fisher 2011 (20) | | RCT |
|  |  |  |  | Conesa 2012 (69) | | NRCT |
|  |  |  |  | Wong 2012 (3) | | NRCT |
| Trunk rehabilitation using unstable surfaces | Van Criekinge 2018  PMID: 28482696 | | | Bae 2013 (16) | | RCT |
|  |  |  |  | Ibrahimi 2010 (30) | | RCT |
|  |  |  |  | Jung 2016 (24) | | RCT |
|  |  |  |  | Karthikbabu 2011 (30) | | RCT |
|  |  |  |  | Lee 2014 (20) | | RCT |
|  |  |  |  | Park 2014 (40) | | RCT |
|  |  |  |  | Yoo 2014 (24) | | RCT |
| Treadmill training | Tally 2017  PMID: 28687056 | | | Chen 2014 (30) | | RCT |
|  |  |  |  | Cho 2015 (31) | | RCT |
|  |  |  |  | Choi 2015 (37) | | RCT |
|  |  |  |  | Globas 2012 (36) | | RCT |
|  |  |  |  | Hwang 2015 (30) | | RCT |
|  |  |  |  | Kang 2016 (30) | | RCT |
|  |  |  |  | Kim 2011 (44) | | RCT |
|  |  |  |  | Kim 2015 (37) | | RCT |
| Bilateral standing with visual feedback therapy | Van Peppen 2006 PMID: 16548079 | | | Chen 2002 (41) | | RCT |
|  |  |  |  | Grant 1997 (16) | | RCT |
|  |  |  |  | Greiger 2001 (13) | | RCT |
|  |  |  |  | Sackley 1997 (25) | | RCT |
|  |  |  |  | Shumway-Cook 1988 (16) | | RCT |
|  |  |  |  | Walker 2000 (32) | | RCT |
|  |  |  |  | Winstein 1989 (34) | | NRCT |
| Transcutaneous electrical nerve stimulation | Lin 2018 PMID: 28862711 | | | Park 2014 (29) | | RCT |
|  |  |  |  | Yan 2009 (38) | | RCT |
| Sling exercise training | Chen L 2016  PMID: 27727288 | | | Lee 2014 (20) | | RCT |
|  |  |  |  | Fu Jianming 2012 (20) | | RCT |
|  |  |  |  | Gu Shaohua 2013 (24) | | RCT |
|  |  |  |  | Li Ziqiang 2013 (80) | | RCT |
|  |  |  |  | Hu Chuan 2015 (60) | | RCT |
| Strengthening of the lower limbs | Wist 2016  PMID: 26969343 | | | Byun 2011 (30) | | RCT |
|  |  |  |  | Flansbjer 2008 (24) | | RCT |
|  |  |  |  | Flansbjer 2012 (18) | | RCT |
|  |  |  |  | Janssen 2008 (12) | | RCT |
|  |  |  |  | Jin 2012 (133) | | RCT |
|  |  |  |  | Ouellette 2004 (42) | | RCT |
|  |  |  |  | Tung 2010 (32) | | RCT |
| Trunk training exercises | Cabanas-Valdés 2013  PMID: 24018373 | | | Dean 1997 (19) | | RCT |
|  |  |  |  | Mudie 2002 (40) | | RCT |
|  |  |  |  | Pollock 2002 (28) | | RCT |
|  |  |  |  | Howe 2005 (35) | | RCT |
|  |  |  |  | Dean 2007 (12) | | RCT |
|  |  |  |  | Ibrahimi 2010 (30) | | RCT |
|  |  |  |  | Verheyden 2009 (33) | | RCT |
|  |  |  |  | Karthikbabu 2011 (30) | | RCT |
|  |  |  |  | Saeys 2011 (33) | | RCT |
|  |  |  |  | Kumar 2011 (20) | | RCT |
|  |  |  |  | Lee 2012 (28) | | RCT |
| Exercise-Based Rehabilitation | An 2011  PMID: 22089406 | | | Au-Yeung 2009 (136) | | RCT |
|  |  |  |  | Barbeau 2003 (100) | | RCT |
|  |  |  |  | Yen 2008 (14) | | RCT |
|  |  |  |  | Noh 2008 (25) | | RCT |
|  |  |  |  | Duncan 2003 (92) | | RCT |
|  |  |  |  | Langhammer 2009 (75) | | RCT |
|  |  |  |  | Pang 2005 (63) | | RCT |
|  |  |  |  | Bayouk 2006 (16) | | RCT |
|  |  |  |  | Marigold 2005 (61) | | RCT |
|  |  |  |  | Yelnik 2008 (68) | | RCT |
| Bobath Concept | Kollen 2009  PMID: 19182079 | | | Mudie 2002 (40) | | RCT |
|  |  |  |  | Pollock 2002 (20) | | RCT |
|  |  |  |  | VanVliet 2005 (120) | | RCT |
|  |  |  |  | Wang 2005 (44) | | RCT |
| Cognitive motor interference | Wang 2015  PMID: 25560629 | | | Her 2011 (38) | | RCT |
|  |  |  |  | Zheng 2012 (85) | | RCT |
|  |  |  |  | Seo 2012 (40) | | RCT |
|  |  |  |  | Cho 2012 (22) | | RCT |
|  |  |  |  | Cho 2013 (14) | | RCT |
|  |  |  |  | Kim 2009 (22) | | RCT |
|  |  |  |  | Yang 2011 (14) | | RCT |
|  |  |  |  | Yang 2008 (20) | | RCT |
|  |  |  |  | Jung 2012 (21) | | RCT |
| Physiotherapy interventions aimed at restoring balance without extensive technical equipment | Hammer 2008  doi: 10.1080/14038190701757656 | | | Bagley 2005 (115) | | RCT |
|  |  |  |  | Blennerhassett 2004 (30) | | RCT |
|  |  |  |  | Duncan 2003 (92) | | RCT |
|  |  |  |  | Marigold 2005 (42) | | RCT |
|  |  |  |  | Pang 2005 (60) | | RCT |
|  |  |  |  | Salbach 2004 (80) | | RCT |
|  |  |  |  | Bonan 2004 (20) | | RCT |
|  |  |  |  | Duncan 1998 (20) | | RCT |
|  |  |  |  | Howe 2005 (31) | | RCT |
|  |  |  |  | McClellan 2004 (23) | | RCT |
|  |  |  |  | Tang 2005 (47) | | RCT |
|  |  |  |  | Wang 2005 (44) | | RCT |
|  |  |  |  | Dean 2000 (8) | | RCT |
|  |  |  |  | Morioka 2003 (26) | | RCT |
| Lumbar stabilization exercises | Ko 2014  PMID: 25540515 | | | Kim 2011 (40) | | RCT |
|  |  |  |  | Saeys 2011 (33) | | RCT |
|  |  |  |  | Verheyden 2009 (33) | | RCT |
|  |  |  |  | Karthikbabu 2011 (30) | | RCT |
|  |  |  |  | Bae 2013 (16) | | RCT |
|  |  |  |  | Chung 2013 (16) | | RCT |
| Lower limb reciprocal pedalling exercise | Hancock 2012  PMID: 22111955 | | | Katz-Leurer 2006 (24) | | RCT |
|  |  |  |  | Quaney 2009 (38) | | RCT |
| Exercise therapy | van Duijnhoven 2016  PMID: 27633021 | | | Au-Yeung 2009 (108) | | RCT |
|  |  |  |  | Cho KH 2012 (22) | | RCT |
|  |  |  |  | Farqalit 2013 (40) | | RCT |
|  |  |  |  | Gok 2008 (30) | | RCT |
|  |  |  |  | Hung 2014 (28) | | RCT |
|  |  |  |  | Kim 2013 (18) | | RCT |
|  |  |  |  | Kim 2009 (24) | | RCT |
|  |  |  |  | Lee 2014 (21) | | RCT |
|  |  |  |  | Lee 2012 (40) | | RCT |
|  |  |  |  | Llorens 2015 (20) | | RCT |
|  |  |  |  | Marigold 2005 (48) | | RCT |
|  |  |  |  | Noh 2008 (20) | | RCT |
|  |  |  |  | Chen 2014 (30) | | RCT |
|  |  |  |  | Cho 2014 (30) | | RCT |
|  |  |  |  | Cho 2013 (28) | | RCT |
|  |  |  |  | Dias 2007 (40) | | RCT |
|  |  |  |  | Globas 2012 (36) | | RCT |
|  |  |  |  | Kang 2012 (30) | | RCT |
|  |  |  |  | Middleton 2014 (38) | | RCT |
|  |  |  |  | Page 2008 (7) | | RCT |
|  |  |  |  | Peurala 2005 (45) | | RCT |
|  |  |  |  | Wang 2015 (51) | | RCT |
|  |  |  |  | Westlake 2009 (16) | | RCT |
|  |  |  |  | Wu 2014 (28) | | RCT |
|  |  |  |  | Yen 2008 (14) | | RCT |
|  |  |  |  | Yang 2011 (14) | | RCT |
|  |  |  |  | Bayouk 2006 (16) | | RCT |
|  |  |  |  | Bonan 2004 (20) | | RCT |
|  |  |  |  | Brogardh 2012 (31) | | RCT |
|  |  |  |  | Cha 2014 (20) | | RCT |
|  |  |  |  | Lau 2012 (82) | | RCT |
|  |  |  |  | Marin 2013 (20) | | RCT |
|  |  |  |  | Tankisheva 2014 (15) | | RCT |
|  |  |  |  | Chu 2004 (12) | | RCT |
|  |  |  |  | Jin 2012 (133) | | RCT |
|  |  |  |  | Pang 2005 (63) | | RCT |
|  |  |  |  | Quaney 2009 (38) | | RCT |
|  |  |  |  | Choi 2015 (37) | | RCT |
|  |  |  |  | Immink 2014 (22) | | RCT |
|  |  |  |  | Pandian 2014 (39) | | RCT |
|  |  |  |  | Rydwik 2006 (17) | | RCT |
|  |  |  |  | Schmid 2012 (39) | | RCT |
|  |  |  |  | Jiejiao 2012 (92) | | RCT |
| Balance training | Lubetzky-Vilnay 2010  PMID: 20716987 | | | Allison 2007 (14) | | RCT |
|  |  |  |  | English 2007 (62) | | RCT |
|  |  |  |  | Pyoria 2007 (68) | | RCT |
|  |  |  |  | Langhammer 2008/2009 (65) | | RCT |
|  |  |  |  | Hidler 2009 (63) | | RCT |
|  |  |  |  | Chan 2006 (52) | | RCT |
|  |  |  |  | Yavuzer 2006 / Eser 2008 (41) | | RCT |
|  |  |  |  | Yelnik 2008 (68) | | RCT |
|  |  |  |  | Srivastava 2009 (40) | | NRCT |
|  |  |  |  | Olawale 2006 (23) | | NRCT |
|  |  |  |  | Fritz 2007 (8) | | NRCT |
|  |  |  |  | Yen 2008 (14) | | RCT |
|  |  |  |  | Gok 2008 (30) | | RCT |
|  |  |  |  | Bayouk 2006 (16) | | RCT |
|  |  |  |  | Leroux 2006 (10) | | NRCT |
|  |  |  |  | Macko 2008 (20) | | NRCT |
|  |  |  |  | Huijbregts 2008 (30) | | RCT |
|  |  |  |  | Huijbregts 2009 (16) | | RCT |
|  |  |  |  | Michael 2009 (7) | | NRCT |
|  |  |  |  | Stuart 2009 (78) | | RCT |
| Physical therapy | Van Peppen 2004  PMID: 15609840 | | | Engardt 1993 (40) | | RCT |
|  |  |  |  | Visintin 1998 (100) | | RCT |
|  |  |  |  | Mudie 2002 (40) | | RCT |
|  |  |  |  | Dean 2000 (12) | | RCT |
|  |  |  |  | Dean 1997 (20) | | RCT |
|  |  |  |  | de Sèze 2001 (20) | | RCT |
|  |  |  |  | Pollock 2002 (28) | | RCT |
|  |  |  |  | Cheng 2001 (54) | | RCT |
|  |  |  |  | Shumway-Cook 1988 (16) | | RCT |
|  |  |  |  | Wong 1997 (60) | | RCT |
|  |  |  |  | Sackley 1997 (26) | | RCT |
|  |  |  |  | Grant 1997 (16) | | RCT |
|  |  |  |  | Lin 1998 (8) | | RCT |
|  |  |  |  | Walker 2000 (30) | | RCT |
|  |  |  |  | Geiger 2001 (13) | | RCT |
|  |  |  |  | Winstein 1989 (42) | | NRCT |
|  |  |  |  | Morioka 2003 (26) | | RCT |
|  |  |  |  | Nilsson 2001 (73) | | RCT |
|  |  |  |  | Trueblood 2001 (10) | | NRCT |
|  |  |  |  | Pomeroy 2001 (24) | | RCT |
| Additional physiotherapy | Bank 2016  PMID: 26086177 | | | Allison 2007 (15) | | RCT |
|  |  |  |  | Bagley 2005 (109) | | RCT |
|  |  |  |  | Chen 2011 (33) | | RCT |
|  |  |  |  | de Sèze 2001 (20) | | RCT |
|  |  |  |  | Ferrante 2007 (22) | | RCT |
|  |  |  |  | Franceschini 2009 (97) | | RCT |
|  |  |  |  | Kim 2012 (20) | | RCT |
|  |  |  |  | Kumar 2013 (20) | | RCT |
|  |  |  |  | Pollock 2002 (26) | | RCT |
|  |  |  |  | Saeys 2012 (33) | | RCT |
|  |  |  |  | Verheyden 2009 (33) | | RCT |
| Additional trunk exercises | Sorinola 2014 PMID: 24990030 | | | De Seze 2001 (20) | | RCT |
|  |  |  |  | Saeys 2011 (33) | | RCT |
| Interventions on improving balance self-efficacy | Tang 2015 PMID: 25681409 | | | Dickstein 2013 (23) | | RCT |
|  |  |  |  | Dickstein 2014 (16) | | RCT |
|  |  |  |  | Holmgren 2010 (34) | | RCT |
|  |  |  |  | Huijbregts 2008 (24) | | NRCT |
|  |  |  |  | Hung 2014 (28) | | RCT |
|  |  |  |  | Hwang 2010 (24) | | RCT |
|  |  |  |  | Jung 2012 (21) | | RCT |
|  |  |  |  | Lau 2012 (82) | | RCT |
|  |  |  |  | Lindvall 2014 (46) | | RCT |
|  |  |  |  | Lord 2008 (30) | | RCT |
|  |  |  |  | Marigold 2005 (48) | | RCT |
|  |  |  |  | Mudge 2009 (58) | | RCT |
|  |  |  |  | Pang 2008 (60) | | RCT |
|  |  |  |  | Pang 2010 (21) | | NRCT |
|  |  |  |  | Park 2011 (25) | | RCT |
|  |  |  |  | Salbach 2005 (83) | | RCT |
|  |  |  |  | Schmid 2012 (47) | | RCT |
|  |  |  |  | Schuster 2012 (39) | | RCT |
|  |  |  |  | Yang 2008 (20) | | RCT |
| Interventions for motor recovery | Langhorne 2009 PMID: 19608100 | | | Duncan 1998 (20) | | RCT |
|  |  |  |  | Duncan 2003 (92) | | RCT |
|  |  |  |  | Richards 1993 (15) | | RCT |
|  |  |  |  | Salbach 2004 (91) | | RCT |
|  |  |  |  | Lee 1996 (59) | | RCT |
|  |  |  |  | Sackley 1997 (26) | | RCT |
|  |  |  |  | Shumway-Cook 1988 (16) | | RCT |
|  |  |  |  | Wong 1997 (60) | | RCT |
|  |  |  |  | Dickstein 1991 (16) | | RCT |
|  |  |  |  | Hocherman 1984 (24) | | RCT |
|  |  |  |  | De Seze 2001 (20) | | RCT |
|  |  |  |  | McClellan 2004 (26) | | RCT |
| Walking with Ankle-Foot Orthosis | Tyson 2013 PMID: 23416220 | | | Alvin 1988 (8) | | RCT |
|  |  |  |  | Chen 1999 (24) | | RCT |
|  |  |  |  | Pohl 2006 (28) | | RCT |
|  |  |  |  | Simons 2009 (20) | | RCT |
|  |  |  |  | Wang 2005 (103) | | RCT |
| Early cardiovascular exercise | Stoller 2012 PMID: 22727172 | | | Duncan 1998 (20) | | RCT |
|  |  |  |  | Duncan 2003 (100) | | RCT |
|  |  |  |  | Outermans 2010 (43) | | RCT |
| Physical therapy | Veerbeek 2014 PMID: 24505342 | | | Dean 1997 (19) | | RCT |
|  |  |  |  | de Sèze 2001 (20) | | RCT |
|  |  |  |  | Mudie 2002 (40) | | RCT |
|  |  |  |  | Dean 2007 (12) | | RCT |
|  |  |  |  | Ibrahimi 2010 (30) | | RCT |
|  |  |  |  | Barreca 2004 (48) | | RCT |
|  |  |  |  | Britton 2008 (18) | | RCT |
|  |  |  |  | Varoqui 2011 (23) | | RCT |
|  |  |  |  | Morioka 2003 (26) | | RCT |
|  |  |  |  | Bagley 2005 (140) | | RCT |
|  |  |  |  | Bayouk 2006 (16) | | RCT |
|  |  |  |  | Allison 2007 (17) | | RCT |
|  |  |  |  | Shumway-Cook 1988 (16) | | RCT |
|  |  |  |  | Grant 1997 (16) | | RCT |
|  |  |  |  | Sackley 1997 (26) | | RCT |
|  |  |  |  | Walker 2000 (32) | | RCT |
|  |  |  |  | Kerdoncuff 2004 (27) | | RCT |
|  |  |  |  | Heller 2005 (26) | | RCT |
|  |  |  |  | Yavuzer 2006 (41) | | RCT |
|  |  |  |  | Gok 2008 (30) | | RCT |
|  |  |  |  | Goljar 2010 (39) | | RCT |
|  |  |  |  | Bonan 2004 (20) | | RCT |
|  |  |  |  | McClellan 2004 (26) | | RCT |
|  |  |  |  | Howe 2005 (35) | | RCT |
|  |  |  |  | Marigold 2005 (48) | | RCT |
|  |  |  |  | Yelnik 2008 (68) | | RCT |
|  |  |  |  | Verheyden 2009 (33) | | RCT |
|  |  |  |  | Askim 2010 (62) | | RCT |
|  |  |  |  | Karthikbabu 2011 (30) | | RCT |
|  |  |  |  | Merkert 2011 (30) | | RCT |
|  |  |  |  | Visintin 1998 (100) | | RCT |
|  |  |  |  | Barbeau 2003 (100) | | RCT |
|  |  |  |  | Nilsson 2001 (73) | | RCT |
|  |  |  |  | Suputtitada 2004 (48) | | RCT |
|  |  |  |  | Sullivan 2007 (71) | | RCT |
|  |  |  |  | Yen 2008 (14) | | RCT |
|  |  |  |  | Westlake 2009 (16) | | RCT |
|  |  |  |  | Moore 2010 (20) | | RCT |
|  |  |  |  | Takami 2010 (36) | | RCT |
|  |  |  |  | Peurala 2005 (45) | | RCT |
|  |  |  |  | Tong 2006 (50) | | RCT |
|  |  |  |  | Dias 2007 (40) | | RCT |
|  |  |  |  | Husemann 2007 (30) | | RCT |
|  |  |  |  | Hornby 2008 (48) | | RCT |
|  |  |  |  | Ng 2008 (53) | | RCT |
|  |  |  |  | Hidler 2009 (63) | | RCT |
|  |  |  |  | Fisher 2011 (20) | | RCT |
|  |  |  |  | Liston 2000 (18) | | RCT |
|  |  |  |  | Lau 2011 (26) | | RCT |
|  |  |  |  | Salbach 2004 (91) | | RCT |
|  |  |  |  | Salbach 2005 (83) | | RCT |
|  |  |  |  | Pang 2005 (63) | | RCT |
|  |  |  |  | Sungkarat 2011 (35) | | RCT |
|  |  |  |  | Lord 2008 (30) | | RCT |
|  |  |  |  | Park 2011 (25) | | RCT |
|  |  |  |  | Blennerhassett 2004 (30) | | RCT |
|  |  |  |  | Yang 2006 (48) | | RCT |
|  |  |  |  | Mead 2007 (66) | | RCT |
|  |  |  |  | Chu 2004 (12) | | RCT |
|  |  |  |  | Noh 2008 (20) | | RCT |
|  |  |  |  | Lynch 2007 (21) | | RCT |
|  |  |  |  | Chen 2011 (33) | | RCT |
|  |  |  |  | Janssen 2008 (12) | | RCT |
|  |  |  |  | Cheng 2010 (15) | | RCT |
| Traditional Chinese exercises | Ge 2017  PMID: 29122267 | | | Kim 2015 (22) | | RCT |
|  |  |  |  | Au-Yeung 2009 (114) | | RCT |
|  |  |  |  | Zhou L 2015 (40) | | RCT |
|  |  |  |  | Zhou ZG 2013 (68) | | RCT |
|  |  |  |  | Wang XB 2016 (30) | | RCT |
|  |  |  |  | Xie 2008 (48) | | RCT |
|  |  |  |  | Huang 2016 (16) | | RCT |
|  |  |  |  | Wang XY 2016 (50) | | RCT |
|  |  |  |  | Yang ZB 2013 (100) | | RCT |
|  |  |  |  | Li 2012 (40) | | RCT |
|  |  |  |  | Zhou QA 2010 (68) | | RCT |
|  |  |  |  | Fu 2016 (60) | | RCT |
|  |  |  |  | Liu 2009 (48) | | RCT |
|  |  |  |  | Yang HX 2016 (49) | | RCT |
|  |  |  |  | Xu 2014 (80) | | RCT |
|  |  |  |  | Gao 2012 (158) | | RCT |
|  |  |  |  | Jia 2008 (34) | | RCT |
|  |  |  |  | Zhang Y 2016 (62) | | RCT |
|  |  |  |  | Bai 2011 (60) | | RCT |
|  |  |  |  | Zhang B 2015 (40) | | RCT |
|  |  |  |  | Zhang M 2013 (221) | | RCT |
| Traditional Chinese exercises | Chen BL 2015  PMID: 26291978 | | | Au-Yeung 2009 (136) | | RCT |
|  |  |  |  | Hart 2004 (18) | | RCT |
|  |  |  |  | Taylor-Piliae 2014 (145) | | RCT |
|  |  |  |  | Zhou 2013 (68) | | RCT |
|  |  |  |  | Yang 2013 (100) | | RCT |
|  |  |  |  | Xie 2008 (48) | | RCT |
|  |  |  |  | Zhang 2013 (224) | | RCT |
|  |  |  |  | Bai 2011 (60) | | RCT |
|  |  |  |  | Jia 2008 (34) | | RCT |
| Virtual reality | Chen Ling 2016  PMID: 28053988 | | | Kim 2009 (24) | | RCT |
|  |  |  |  | Yang 2011 (14) | | RCT |
|  |  |  |  | Cho 2012 (24) | | RCT |
|  |  |  |  | Cho 2013 (14) | | RCT |
|  |  |  |  | Fritz 2013 (30) | | RCT |
|  |  |  |  | Cho 2014 (30) | | RCT |
|  |  |  |  | McEwen 2014 (59) | | RCT |
|  |  |  |  | Morone 2014 (50) | | RCT |
|  |  |  |  | Llorens 2015 (20) | | RCT |
| Virtual reality | Luque-Moreno 2015  PMID: 26539480 | | | Kim 2009 (24) | | RCT |
|  |  |  |  | Gil-Gomez 2011 (17) | | RCT |
|  |  |  |  | Fritz 2013 (28) | | RCT |
|  |  |  |  | Cho and Lee 2013/2014 (30) | | RCT |
| Telerehabilitation | Chen J 2015  PMID: 26483155 | | | Lin 2014 (24) | | RCT |
|  |  |  |  | Llorens 2015 (30) | | RCT |
| Rehabilitation with Nintendo Wii | Dos Santos 2015  PMID: 26303792 | | | Barcala 2011 (12) | | RCT |
|  |  |  |  | Cho 2012 (22) | | RCT |
|  |  |  |  | Barcala 2013 (20) | | RCT |
| Rehabilitation with Nintendo Wii | Cheok 2015  PMID: 26253322 | | | Cho 2012 (22) | | RCT |
|  |  |  |  | Barcala 2013 (20) | | RCT |
| Virtual reality | Li 2016  PMID: 26141808 | | | Barcala 2013 (20) | | RCT |
|  |  |  |  | Cho 2012 (22) | | RCT |
|  |  |  |  | Cho 2014 (30) | | RCT |
|  |  |  |  | Hung 2014 (28) | | RCT |
|  |  |  |  | Jung 2012 (21) | | RCT |
|  |  |  |  | Kim 2009 (24) | | RCT |
|  |  |  |  | Kim 2012 (19) | | RCT |
|  |  |  |  | Kim 2015 (17) | | RCT |
|  |  |  |  | Krpic 2013 (15) | | RCT |
|  |  |  |  | Llorens 2014 (20) | | RCT |
|  |  |  |  | McEwen 2014 (59) | | RCT |
|  |  |  |  | Rajaratnam 2013 (19) | | RCT |
|  |  |  |  | Song 2014 (20) | | RCT |
|  |  |  |  | Yang 2008 (20) | | RCT |
| Virtual reality | de Rooji 2016  PMID: 27174255 | | | Llorens 2015 (20) | | RCT |
|  |  |  |  | Cho 2014 (30) | | RCT |
|  |  |  |  | Morone 2014 (50) | | RCT |
|  |  |  |  | Cho 2013 (14) | | RCT |
|  |  |  |  | Rajaratnam 2013 (19) | | RCT |
|  |  |  |  | Barcala 2013 (20) | | RCT |
|  |  |  |  | Kim 2009 (24) | | RCT |
|  |  |  |  | Hung 2014 (28) | | RCT |
|  |  |  |  | Jung 2012 (21) | | RCT |
|  |  |  |  | Kang 2012 (30) | | RCT |
|  |  |  |  | Kim 2015 (17) | | RCT |
|  |  |  |  | Lee 2015 (24) | | RCT |
|  |  |  |  | Lee 2015 (20) | | RCT |
|  |  |  |  | Song 2015 (40) | | RCT |
|  |  |  |  | Song 2014 (20) | | RCT |
|  |  |  |  | Cho 2012 (22) | | RCT |
|  |  |  |  | Yang 2011 (14) | | RCT |
|  |  |  |  | Yang 2008 (20) | | RCT |

| Supplementary Table 3. (Continued) | | | | | | |
| --- | --- | --- | --- | --- | --- | --- |
| Intervention | | Review | Unique trials contributed and participants (n) on balance and postural control | | Study design | |
| Virtual reality | Iruthayarajah 2017 PMID: 27309680 | | | Lee 2015 (24) | | RCT |
|  |  |  |  | Song 2015 (40) | | RCT |
|  |  |  |  | Hung 2014 (28) | | RCT |
|  |  |  |  | Barcala 2013 (20) | | RCT |
|  |  |  |  | Fritz 2013 (28) | | RCT |
|  |  |  |  | Gil-Gomez 2011 (17) | | RCT |
|  |  |  |  | Cho 2014 (30) | | RCT |
|  |  |  |  | Jung 2012 (21) | | RCT |
|  |  |  |  | Yang 2011 (14) | | RCT |
|  |  |  |  | Kim 2009 (24) | | RCT |
|  |  |  |  | Lee 2014 (21) | | RCT |
|  |  |  |  | Llorens 2015 (20) | | RCT |
|  |  |  |  | Llorens 2015 (29) | | RCT |
|  |  |  |  | Yom 2015 (20) | | RCT |
|  |  |  |  | Lee 2015 (21) | | RCT |
|  |  |  |  | Cho 2013 (14) | | RCT |
|  |  |  |  | Kang 2012 (30) | | RCT |
|  |  |  |  | Cho 2012 (22) | | RCT |
|  |  |  |  | Kim 2015 (17) | | RCT |
|  |  |  |  | In-Chul 2012 (28) | | RCT |
| Virtual reality based rehabilitation | Corbetta 2015 PMID: 26093805 | | | Barcala 2013 (20) | | RCT |
|  |  |  |  | Cho 2012 (22) | | RCT |
|  |  |  |  | Cho 2013 (14) | | RCT |
|  |  |  |  | Cho 2014 (30) | | RCT |
|  |  |  |  | Kim 2009 (24) | | RCT |
|  |  |  |  | Llorens 2014 (20) | | RCT |
|  |  |  |  | Morone 2014 (47) | | RCT |
|  |  |  |  | Rajaratnam 2015 (19) | | RCT |
|  |  |  |  | Song 2014 (20) | | RCT |

N/A: Not available; RCT= Randomized Controlled Trial; NRCT= Not Randomized Controlled Trial
