# Supplementary material for: Rehabilitation interventions for improving balance following stroke: An overview of systematic reviews
Source: PLoS One. 2019 Jul 19;14(7):e0219781. doi: 10.1371/journal.pone.0219781 (PMC6641159; doi:10.1371/journal.pone.0219781)
Supplement: S4 Table — (DOCX) [file pone.0219781.s004.docx]

| Supplementary Table 4. Characteristics of included reviews | | | | | | | | | |
| --- | --- | --- | --- | --- | --- | --- | --- | --- | --- |
| **Review** | **Trials (n)** | **Population** | **Interventions** | **Comparison** | **Methodology quality assessment** | **Outcomes** | **Meta-Analysis** | **AMSTAR judgement of review quality** | **Comments** |
| ***Cochrane Reviews (CSRs)*** | | | | | | | | | |
| Barclay-Goddard 2004 | 7 (245) | Stroke survivors found to have either abnormal weight bearing in the standing position, or standing balance impairment during or after initial rehabilitation | Visual or auditory force platform feedback | Conventional treatment, another balance treatment or placebo balance treatment | Jadad (1-3) | BBS, TUGT, Centre of Pressure Position (Stance symmetry), Centre of Pressure Behaviour (sway) | 7 | Moderate |  |
| Bowen 2013 | 0 (0) | Stroke patients with neglect | Cognitive rehabilitation | Any control intervention (standard care, placebo, attention, no treatment or another active cognitive intervention) | RoB Cochrane Tool (NR) | BBS, FRT, ST, Get up and Go test, Standing Balance test |  | High | No other relevant outcome data were reported i.e. balance, quality of life and social isolation, and adverse events (excluding falls) |
| English 2017 | 11 (935) | Stroke | Circuit class therapy | No therapy, sham therapy, or another therapy modality | RoB Cochrane Tool (NR) | TUG, BBS, ST and ABC Scale | 5 | High |  |
| French 2016 | 14 (766) | Stroke | Repetitive task training | Attention control, usual care | RoB Cochrane Tool (NR) | Sitting balance/reach: Reaching distance, Sitting Equilibrium Index, MAS - Balance Sitting subscale, Lateral reach - time to return to quiet sitting  Standing balance/reach: BBS, Upright Equilibrium Index, FRT, ABC Scale, TBT | 2 | High | Several outcomes included in two macro-outcomes |

| Supplementary Table 4. (Continued) | | | | | | | | | |
| --- | --- | --- | --- | --- | --- | --- | --- | --- | --- |
| Review | Trials (n) | Population | Interventions | Comparison | Methodology quality assessment | Outcomes | Meta-Analysis | AMSTAR judgement of review quality | Comments |
| Laver 2017 | 13 (320) | Stroke | Virtual reality | No intervention or therapy based on a standard-care approach | RoB Cochrane Tool (NR) | BBS, BBA, FRT, POMA, Forward reach test, FES, PASS, BPM | 2 | High | Four studies excluded from meta-analysis because of inability to obtain data required |
| Lawrence 2017 | 2 (69) | Stroke | Yoga | A waiting-list control or no intervention control | RoB Cochrane Tool: high risk of bias | BBS, ABC Scale | 2 MA 1 Sensitivity analysis | High |  |
| Mehrholz 2011 | 2 (38) | Stroke | Water-based exercises | All other non-water-based interventions | RoB Cochrane Tool (NR) PEDro Scale (5-6) | BBS | 1 | High |  |
| Pollock 2011 (a) | 0 (0) | Stroke patients with a clinical diagnosis of an eye movement disorder | Interventions for eye movement disorders | No treatment, placebo, a control intervention or standard care | RoB Cochrane Tool (NR) | BBS, FRT, ST, Get up and Go test, Standing Balance test |  | High | Not able to draw conclusions from their studies on balance outcomes |
| Pollock 2011 (b) | 0 (0) | Stroke patients with visual field defects | Intervention specifically targeted at improving the visual field defect or improving the ability of the participant to cope with the visual field loss | No treatment, placebo and control | RoB Cochrane Tool (NR) | BBS, FRT, ST, Get up and Go test, Standing Balance test |  | High | Not able to draw conclusions from their studies on balance outcomes |
| Pollock 2014 | 11 (509) | Stroke (both ischemic and hemorrhagic) | Physical rehabilitation | No treatment, usual care or attention control, another active intervention | RoB Cochrane Tool (NR) | BBS | 4 | High | Yelnik 2008 (68) not included in meta-analysis |
| Saunders 2016 | 19 (1128) | Stroke | Physical fitness training | Usual care, no intervention or a non-exercise intervention | RoB Cochrane Tool (NR) | BBS, FRT, TIS, Four Square Step Test, TBT, Postural Sway | 14 | High |  |
| Vloothuis 2016 | 3 (139) | Stroke | Caregiver-mediated exercise in addition to usual care or instead of usual care | Usual care | RoB Cochrane Tool: 1 unclear risk of bias, 2 low risk of bias | BBS, PASS | 5 MA 1 Sensitivity analysis | High |  |
| ***Non-Cochrane Reviews (non-CSRs)*** | | | | | | | | | |
| An 2011 | 10 (650) | Stroke (both acute and chronic) | Exercise-Based Rehabilitation | Control intervention | PEDro Scale (4-8): 7 High quality, 3 Lower quality | BBS, Shifting center of gravity movements, TUGT, SRT, COP variability and total excursion |  | Critically low | Most of the trials (8 out of 10) assessed balance using the BBS |
| Bank 2016 | 11 (428) | Stroke | Additional physiotherapy to standard physiotherapy | Standard physiotherapy | PEDro Scale (4-7) | TCT, TIS, PASS-TC, Symmetry Index, MAS - Balance Sitting subscale, Sitting Equilibrium Index | 2 | Low | Meta-analysis performed only for TCT and TIS outcomes |
| Bonini-Rocha 2018 | 3 (174) | Stroke | Circuit-based exercise | Conventional therapy or no intervention | PEDro Scale (5-8): 2 High quality, 1 low quality RoB Cochrane Tool (NR) | BBS | 1 | Moderate | Meta-analysis analysis included only chronic patients |
| Cabanas-Valdés 2013 | 11 (308) | Stroke (both sub-acute and chronic, both ischemic and hemorrhagic) | Trunk training exercises | Conventional physiotherapy | PEDro Scale (3-8): 6 High quality, 5 low quality | mRT, TIS - Static Sitting Balance subscale, TIS - Dynamic Sitting Balance subscale, TIS - Coordination subscale, BWD, Romberg test, BBS, FTBS, Tinetti score, BBA |  | Critically low |  |
| Chen BL 2015 | 9 (833) | Stroke | Traditional Chinese exercises | No intervention or other treatment | RoB Cochrane Tool: High risk of bias | BBS, TUGT, FMA - Balance subscale, limit of stability, SOT, SPPB - Balance subscale | 1 | Moderate |  |
| Chen J 2015 | 2 (54) | Stroke survivors living at home | Telerehabilitation | Conventional rehabilitation or no rehabilitation | RoB Cochrane Tool: Low risk of bias | BBS | 1 | Low |  |
| Chen L 2016 | 5 (204) | Stroke manifesting balance dysfunction | Sling exercise training | Conventional rehabilitation treatments | Modified Jadad Scale | BBS, SA, SL, Bio Rescue measures, PASS | 1 MA 1 Sensitivity analysis | Critically low |  |
| Chen Ling 2016 | 9 (265) | Stroke (acute, sub-acute and chronic stage) | Virtual reality | Control intervention | PEDro Scale (4-9): 2 fair quality, 6 good quality, 1 excellent quality | BBS, TUGT, Static and Dynamic balance assessed by force platform |  | Critically low | Stroke stages: acute (1 study), sub-acute (1 study), chronic (7 studies) |
| Cheok 2015 | 2 (42) | Stroke | Additional Wii | Standard care | PEDro Scale (5-8): 1 good quality, 1 fair quality RoB Cochrane Tool (NR) | BBS, Postural sway measures (AP eyes open and closed, ML eyes open and closed) | 2 | Low |  |
| Corbetta 2015 | 9 (216) | Stroke | Virtual reality based rehabilitation replacing some or all of standard rehabilitation or virtual reality based rehabilitation used as extra rehabilitation time added to a standard rehabilitation regimen | No treatment or standard rehabilitation | RoB Cochrane Tool (NR) | BBS | 1 | Low |  |
| de Rooji 2016 | 18 (433) | Stroke | Balance training using Virtual reality | Conventional balance training | PEDro Scale (3-8): 11 High quality, 7 Lower quality | BBS, TUGT | 3 MA 1 Sensitivity analysis | Moderate | Meta-analysis performed only of studies with a PEDro Score > 6 |
| Dos Santos 2015 | 3 (54) | Stroke survivors aged 18-70 | Rehabilitation with Nintendo Wii | Conventional physiotherapy | PEDro Scale (4-7) | BBS, TUGT, Pressure platforms |  | Critically low |  |
| Ge 2017 | 21 (1408) | Stroke | Traditional Chinese exercises | Routine rehabilitation therapy or exercise | RoB Cochrane Tool (NR) | BBS, TUGT, FMA - Balance subscale | 8 MA 6 Sensitivity analysis | Low | Heterogeneity before and after sensitivity analysis |
| Hammer 2008 | 14 (638) | Stroke (acute, sub-acute and late phase) | Physiotherapy interventions aimed at restoring balance without extensive technical equipment | Control intervention | PEDro Scale (6-8): 6 High quality, 6 Medium quality, 2 Low quality | BBS, FRT, TUGT, MAS, STREAM, RMI, Posturography, ST, SRT |  | Critically low | Studies with PEDro Scores of < 5 points were excluded |
| Hancock 2012 | 2 (62) | Stroke (both acute and chronic) | Lower limb reciprocal pedalling exercise | Routine therapy, sham exercise, passive exercise, home-based program of stretching exercises | RoB Cochrane Tool (NR) | PASS, PASS - Static subscale, PASS - Dynamic subscale, BBS, Get Up and Go |  | Low | Systematic review of randomized (5) and nonrandomized (7) studies: only 2 studies, both RCTs, focusing on balance |
| Iruthayarajah 2017 | 20 (468) | Chronic stroke | Virtual reality (isolated or in combination with other therapies) | Alternative rehabilitation therapy | PEDro Scale (5-8): 17 good quality, 3 fair quality | Dynamic Balance: BBS, TUGT, FRT, 6MWT, 1MWT, 10MWT, 3MWT, ART, TST, 30SST, POMA, BBA  Static Balance: COP path lengths and oscillations, Limit of stability, Postural sway path length and velocity, Stability Index, BWD, Symmetry Index | 8 | Critically low | Meta-analysis performed only of BBS and TUGT outcomes |
| Ko 2014 | 6 (168) | Stroke (both acute and chronic) | Lumbar stabilization exercises (on unstable bases of support) | Conventional physiotherapy, general exercise intervention or lumbar stabilization exercises on stable bases of support | PEDro Scale (5-8) | TIS, TIS - Static Sitting Balance subscale, TIS - Dynamic Sitting Balance subscale, BBS, BBA, FRT, Tinetti test, Romberg eyes open, Romberg eyes closed, FTBS, SA, SP |  | Critically low |  |
| Kollen 2009 | 4 (224) | Stroke | Bobath Concept | Control intervention | PEDro Scale (4-8): 4 High quality | BWD over hemiplegic and non-hemiplegic sides, MAS, BBS |  | Critically low | Studies with PEDro Scores of > 4 points were classified as "high quality", whereas scores of < 3 points were classified as "low quality" |
| Langhorne 2009 | 12 (465) | Stroke | Interventions for motor recovery | No treatment, placebo, or standard care | N/R | BBS, BWD, Postural sway during sitting and standing | 7 | Moderate |  |
| Li 2016 | 14 (334) | Stroke (both acute, subacute and chronic stage) | Virtual reality | Usual care, placebo control intervention, or any other exercise intervention without virtual reality | RoB Cochrane Tool (2-4): 3 valued 2 points, 5 valued 3 points, 5 valued 4 points | BBS, TUGT, FRT, ABC Scale, BBA, Tinetti Gait and Balance Test, Sway velocity, BWD | 6 | Moderate |  |
| Lin 2018 | 2 (67) | Stroke patients able to walk independently | Transcutaneous electrical nerve stimulation (TENS) | Placebo transcutaneous electrical nerve stimulation (TENS) | Jadad Scale: High quality | TUGT, Postural sway velocity | 3 | Low | An article with Jadad Scale score < 2 was considered to be of low quality, > 3 high quality |
| Lu 2015 | 3 (133) | Chronic stroke (both ischemic and hemorrhagic) | Whole Body Vibration | No intervention, the same exercise without vibration or placebo vibrating platform | RoB Cochrane Tool (NR) | BBS | 1 | Critically low | Marin 2008 (20) not included in meta-analysis  No significant heterogeneity was found among the included studies, so fixed-effect model was used |
| Lubetzky-Vilnay 2010 | 20 (725) | Stroke (acute, sub-acute and chronic stage) | Balance training | Control intervention | American Academy of Cerebral Palsy and Developmental Medicine Scale: 5-point scale from Level I to level V; within each level, quality was assessed based on 7 internal and external validity characteristics (Level I-IV, quality rating 4,5-7) | BBS, Postural Control and Balance Test, force platform measures of balance index, dynamic limits of stability, Brunnstrom stage, Number of falls, FMA - Balance subscale, Balance Index on the Kinesthetic Ability Trainer, COP displacement, ABC Scale, FES, DGI |  | Critically low | Only studies that scored 4 and above in quality rating were considered Langhammer 2008 (65) and Langhammer 2009 (65) / Yavuzer 2006 (41) and Eser 2008 (41) reported their outcomes in two separate publications: considered only once |
| Luque-Moreno 2015 | 4 (99) | Stroke | Virtual reality | Alternative intervention or no intervention | PEDro Scale (6-7) | BBS, ART, BBA, TUGT, ST, TST, 1MWT, 10MWT, 30SST, BPM, Postural sway, FMA |  | Critically low | All patients are in chronic stage Cho and Lee 2013/Cho and Lee 2014 published study data from the same trial in different articles |
| Sorinola 2014 | 2 (53) | Acute stroke (both ischemic or haemorrhagic) | Additional trunk exercises | Conventional rehabilitation | RoB Cochrane Tool: one moderate and one low risk of bias PEDro Scale (6-7) | Upright equilibrium index, Tinetti Scale | 1 | Low |  |
| Stoller 2012 | 3 (163) | Acute or subacute stroke (< 6 months) | Early cardiovascular exercise | Usual care | PEDro Scale (6-8): good quality | BBS, FRT | 0 | Moderate | Study quality was defined using PEDro scores as follows: "excellent" 9-10 points, "good" 6-8 points, "fair" 4-5 points, and "poor" < 3 points |
| Swinnen 2014 | 9 (359) | Stroke | Robot-assisted gait training | Other gait rehabilitation methods | Evaluation of Quality of an Intervention Study checklist (56-81%) 7 true experimental, 3 pre-experimental studies | BBS, TUGT, Tinetti test, Postural sway tests, Romberg test |  | Critically low | Studies with a Evaluation of Quality of an Intervention Study checklist below 50% were excluded |
| Tally 2017 | 8 (275) | Chronic stroke (> 6 months) | Treadmill training, isolated or with adjunctive interventions | Conventional physical therapy treatments | PEDro Scale (5-9): 7 High quality, 1 Lower quality | BBS, combination of directional postural sway and limits of stability assessment |  | Critically low | Studies prior to 2007 were removed |
| Tang 2015 | 19 (729) | Stroke | Interventions on improving balance self-efficacy | Control intervention (several control interventions) | PEDro Scale (3-8): 1 poor, 4 fair, 14 good quality | Balance self-efficacy: ABC Scale, FES-International and FES-Swedish version | 3 MA 3 Sensitivity analysis | Low | Study quality was defined using PEDro scores as follows: "good" 6-8 points, "fair" 4-5 points, and "poor" < 3 points |
| Tyson 2013 | 5 (183) | Stroke | Walking with Ankle-Foot Orthosis | Walking without Ankle-Foot Orthosis | RoB Cochrane Tool: low risk of bias | BBS, Postural sway, BWD while standing | 2 | Moderate | Participants acted as their own controls (when walking without the orthosis): crossover design  Studies rated as having a low risk of bias were selected for the analysis |
| Van Criekinge 2018 | 7 (184) | Stroke (both ischemic and hemorrhagic) | Trunk rehabilitation using unstable surfaces | Trunk rehabilitation using stable surfaces, whether or not combined with conventional therapy | PEDro Scale (4-8): 6 high risk of bias and 1 low risk of bias | Sitting balance: TIS, TIS - Static Sitting Balance subscale, TIS - Dynamic Sitting Balance subscale, TIS - Coordination subscale, MAS - Balance Sitting subscale  Standing balance: BBS, centre of gravity displacements, BBA - Standing subscale, MAS - Sitting to standing subscale, FICSIT-4 | 2 | Moderate | Partial meta-analysis |
| van Duijnhoven 2016 | 43 (1522) | Chronic stroke (> 6 months) | Exercise therapy | Usual care or no intervention | PEDro Scale (4-9): 34 High quality, 9 Moderate quality | BBS, FRT, SOT, Mean postural sway velocity | 10 | Moderate | Studies with PEDro Scores of > 6 points were classified as "high quality", 4-5 points as "moderate quality", whereas scores of < 3 points were classified as "low quality"  3 study excluded from further analysis because of low quality (PEDro Scale < 4) |
| Van Peppen 2004 | 20 (658) | Stroke | Physical therapy | Control intervention | PEDro Scale (4-7) | Postural symmetry sit-to-stand (BWD, vertical force difference between left and right, peak vertical ground reaction force through affected foot)  Postural symmetry stand-to-sit (BWD, vertical force difference between left and right), Time needed to stand-up, Time needed to sit-down, Postural sway/symmetry, BBS, TUGT | 9 | Critically low | Studies with PEDro Scores of > 4 points were classified as "high quality", whereas scores of < 3 points were classified as "low quality"  Lin 1998 (8), Winstein 1989 (42) and Trueblood 2001 (10) NOT assessed with PEDro Scale  Meta-analysis performed only with RCTs |
| Van Peppen 2006 | 7 (177) | Stroke | Bilateral standing with visual feedback therapy | Conventional therapy | PEDro Scale (3-6) | BWD while bilateral standing, postural sway in bilateral standing, BBS, TUGT | 5 MA 2 Sensitivity analysis | Critically low | All studies were performed within the first 6 months post-stroke |
| Veerbeek 2014 | 64 (2469) | Stroke | Physical therapy | Usual care, another intervention, the same intervention with a different dose, or no intervention | PEDro Scale (2-8) | BBS, BBA, PASS, ST, FRT, LRT, TIS, SRT, FMA, BWD, STS, SST, ABC Scale, Sitting and standing symmetry, Sitting equilibrium test, Reach distance, Posturography, Static balance, Dynamic balance, Tinetti, Postural sway | 22 | Moderate |  |
| Wang 2015 | 9 (276) | Stroke | Cognitive motor interference | Single-task exercise or no treatment | RoB Cochrane Tool: High Risk of bias | SA, SD, BBS, TUGT, ABC Scale | 5 | Low | Yang 2011 (14) not included in meta-analysis |
| Wevers 2009 | 5 (241) | Stroke | Circuit class training | Upper extremity training or no rehabilitation training | PEDro Scale (4-8): high quality | BBS, ST | 2 | Critically low | Studies with PEDro Scores of > 4 points were classified as "high quality", whereas scores of < 3 points were classified as "low quality" |
| Wist 2016 | 7 (291) | Chronic stroke (> 3 months) | Strengthening of the lower limbs | Control intervention | RoB Cochrane Tool (NR) | BBS, TUGT | 2 | Low |  |
| Yang 2015 | 4 (186) | Stroke (both acute and chronic, both ischemic and hemorrhagic) | Whole Body Vibration | No intervention, sham vibration, routine physiotherapy or exercise therapy on music | RoB Cochrane Tool (NR) | BBS | 1 | Moderate | No significant heterogeneity was found among the included studies, so fixed-effect model was used |

10MWT= 10-Meter Walking Test; 1MWT= 1-Minute Walking Test; 30SST= 30-Second Sit to Stand Test; 3MWT= 3-Meter Walking Test; 6MWT= 6-Minute Walking Test; ABC Scale= Activities Based Confidence Scale; AP= Anteroposterior; ART= Anterior Reach Test; BBA= Brunel Balance Assessment; BBS= Berg Performance Scale; BPM= Balance Performance Monitor; BWD= Body Weight Distribution; COP= Centre of Pressure; DGI= Dynamic Gait Index; FES= Falls Efficacy Scale; FICSIT-4= Frailty and Injuries Cooperative Studies of Intervention Technique scale; FMA= Fugl-Meyer Assessment; FRT= Functional Reach Test; FTBS= Four Test Balance Scale; MAS= Motor Assessment Scale; ML= Mediolateral; mRT= Modified Reach Test; PASS= Postural Assessment Scale for Stroke patients; PASS-TC= Postural assessment scale for stroke patients - Trunk Control; POMA= Tinetti Performance Oriented Mobility Assessment; RMI= Rivermead Mobility Index; SA= Sway Area of the COP; SD= Sway Distance of the COP; SL= Sway Length of the COP; SOT= Sensory Organization Test; SP= Sway Path of the COP; SPPB= Short Physical Performance Battery; SRT= Step Reaction Time; SST= Single Support Time; ST= Step Test; STREAM= Stroke Rehabilitation Assessment of Movement; STS= Sit-to-stand; TBT= Timed Balance Test; TCT= Trunk Control Scale; TIS= Trunk Impairment Scale; TST= Timed Stair Test; TUGT= Timed Up and Go Test
