# Supplementary material for: Rehabilitation interventions for improving balance following stroke: An overview of systematic reviews
Source: PLoS One. 2019 Jul 19;14(7):e0219781. doi: 10.1371/journal.pone.0219781 (PMC6641159; doi:10.1371/journal.pone.0219781)
Supplement: S5 Table — (DOCX) [file pone.0219781.s005.docx]

| Supplementary Table 5. Overview of reviews | | | | | | | |
| --- | --- | --- | --- | --- | --- | --- | --- |
| **Outcome** | **Contributing reviews** | **Intervention and comparison** | **Relative effect** | **Heterogeneity (I^2^)** | **Number of participants (studies)** | **Quality of evidence (GRADE)** | **Comments** |
| ***Cochrane Reviews (CSRs)*** | | | | | | | |
| BBS | Barclay-Goddard 2004 | Visual force platform feedback vs Conventional treatment, another balance treatment or placebo balance treatment | NO statistically significant differences between groups | 0% | 43 (2) | N/R |  |
| TUGT |  | Visual force platform feedback vs Conventional treatment, another balance treatment or placebo balance treatment | NO statistically significant differences between groups | 21% | 42 (2) | N/R |  |
| Centre of Pressure Position (Stance symmetry) |  | Visual force platform feedback vs Conventional treatment, another balance treatment or placebo balance treatment | Statistically significant | 0% | 41 (2) | N/R |  |
| Centre of Pressure Behaviour (sway) |  | Visual force platform feedback vs Conventional treatment, another balance treatment or placebo balance treatment | NO statistically significant differences between groups | 0% | 71 (3) | N/R |  |
| Centre of Pressure Position (Stance symmetry) |  | Concurrent auditory and visual force platform feedback vs Conventional treatment, another balance treatment or placebo balance treatment | Statistically significant | 0% | 120 (2) | N/R |  |
| Centre of Pressure Position (Stance symmetry) |  | Visual feedback alone plus concurrent auditory and visual feedback vs Conventional treatment, another balance treatment or placebo balance treatment | Statistically significant | 0% | 161 (4) | N/R |  |
| Centre of Pressure Behaviour (sway) |  | Visual force platform feedback vs Conventional treatment, another balance treatment or placebo balance treatment | NO statistically significant differences between groups | 0% | 71 (2) | N/R | Follow up |

| Supplementary Table 5. (Continued) | | | | | | | |
| --- | --- | --- | --- | --- | --- | --- | --- |
| Outcome | Contributing reviews | Intervention and comparison | Relative effect | Heterogeneity (I^2^) | Number of participants (studies) | Quality of evidence (GRADE) | Comments |
| Balance (BBS, FRT, ST, Get up and Go test, Standing Balance test) | Bowen 2013 | Cognitive rehabilitation vs Any control intervention (standard care, placebo, attention, no treatment or another active cognitive intervention) | N/R | N/R | N/R | N/R | No other relevant outcome data were reported i.e. balance, quality of life and social isolation, and adverse events (excluding falls) |
| TUG | English 2017 | Circuit class therapy vs Other interventions | Statistically significant | 0% | 488 (5) | Low |  |
| BBS |  | Circuit class therapy vs Other interventions | NO statistically significant differences between groups | 30% | 171 (4) | N/R |  |
| ST |  | Circuit class therapy vs Other interventions | NO statistically significant differences between groups | 21% | 190 (3) | N/R |  |
| ABC Scale |  | Circuit class therapy vs Other interventions | Statistically significant | 0% | 103 (2) | N/R |  |
| TUGT |  | Circuit class therapy + education vs No intervention | NO statistically significant differences between groups | 0% | 269 (2) | N/R |  |
| Sitting balance/reach | French 2016 | Repetitive task training vs Attention control, usual care | Statistically significant | 48% | 268 (6) | Low | Data available for 222 of 268 participants |
| Standing balance/reach |  | Repetitive task training vs Attention control, usual care | Statistically significant | 0% | 520 (9) | N/R | Data available for 504 of 520 participants |
| Balance (BBS, BBA, Forward Reach Test, FES, POMA) | Laver 2017 | Virtual reality vs Conventional therapy | NO statistically significant differences between groups | 10% | 72 (3) | N/R |  |
| Balance (BBS, BBA, FRT, PASS, BPM) |  | Virtual reality + usual care vs Usual care alone | Statistically significant | 32% | 173 (7) | N/R |  |
| BBS | Lawrence 2017 | Yoga vs Waiting-list control | NO statistically significant differences between groups | 0% | 69 (2) | Very low |  |
| BBS |  | Yoga vs Waiting-list control | NO statistically significant differences between groups | NA | 22 (1) | N/R | Sensitivity analysis: Schmid 2012 (47) excluded because being high risk of bias due to the unrepresentative nature of its sample |
| ABC Scale |  | Yoga vs Waiting-list control | NO statistically significant differences between groups | NA | 47 (1) | N/R |  |

| Supplementary Table 5. (Continued) | | | | | | | |
| --- | --- | --- | --- | --- | --- | --- | --- |
| Outcome | Contributing reviews | Intervention and comparison | Relative effect | Heterogeneity (I^2^) | Number of participants (studies) | Quality of evidence (GRADE) | Comments |
| BBS | Mehrholz 2011 | Water-based exercises vs No water-based exercises | NO statistically significant differences between groups | 81% | 38 (2) | N/R |  |
| Balance (BBS, FRT, ST, Get up and Go test, Standing Balance test) | Pollock 2011 (a) | Interventions for eye movement disorders vs No treatment, placebo, a control intervention or standard care | N/R | N/R | N/R | N/R | Not able to draw conclusions from their studies on balance outcomes |
| Balance (BBS, FRT, ST, Get up and Go test, Standing Balance test) | Pollock 2011 (b) | Intervention specifically targeted at improving the visual field defect or improving the ability of the participant to cope with the visual field loss vs No treatment, placebo and control | N/R | N/R | N/R | N/R | Not able to draw conclusions from their studies on balance outcomes |
| BBS | Pollock 2014 | Intervention vs no treatment | NO statistically significant differences between groups | NA | 34 (1) | Very low | Immediate outcome |
| BBS |  | Intervention vs no treatment | NO statistically significant differences between groups | NA | 34 (1) | Very low | Persisting outcome |
| BBS |  | Intervention vs attention control or usual care | Statistically significant | 0% | 246 (5) | Moderate | Immediate outcome |
| BBS |  | One active intervention vs another active intervention | NO statistically significant differences between groups | 0% | 83 (4) | N/R | Immediate outcome |
| BBS | Saunders 2016 | Cardiorespiratory training vs Control intervention | NO statistically significant differences between groups | 16% | 435 (7) | N/R |  |
| BBS |  | Cardiorespiratory training vs Control intervention | NO statistically significant differences between groups | 9% | 160 (3) | N/R | During usual care |
| BBS |  | Cardiorespiratory training vs Control intervention | NO statistically significant differences between groups | 40% | 275 (4) | N/R | After usual care |
| BBS |  | Cardiorespiratory training vs Control intervention | NO statistically significant differences between groups | 0% | 134 (2) | N/R | Follow up |
| BBS |  | Resistance training vs Control intervention | Statistically significant | NA | 28 (1) | N/R |  |
| AP Sway |  | Resistance training vs Control intervention | Marginal, heterogenous effects | 87% | 61 (2) | N/R |  |
| ML Sway |  | Resistance training vs Control intervention | Marginal, heterogenous effects | 92% | 61 (2) | N/R |  |
| TIS | Saunders 2016 | Resistance training vs Control intervention | NO statistically significant differences between groups | NA | 33 (1) | N/R |  |
| BBS |  | Mixed training vs Control intervention | Statistically significant | 7% | 260 (6) | High |  |
| BBS |  | Mixed training vs Control intervention | NO statistically significant differences between groups | 25% | 140 (4) | N/R | During usual care |
| BBS |  | Mixed training vs Control intervention | Statistically significant | 0% | 120 (2) | N/R | After usual care |
| BBS |  | Mixed training vs Control intervention | NO statistically significant differences between groups | 76% | 102 (2) | N/R | Follow up |
| FRT |  | Mixed training vs Control intervention | NO statistically significant differences between groups | 28% | 166 (2) | N/R |  |
| FRT |  | Mixed training vs Control intervention | NO statistically significant differences between groups | NA | 66 (1) | N/R | Follow up |
| Four Square Step Test |  | Mixed training vs Control intervention | NO statistically significant differences between groups | NA | 28 (1) | N/R | Baseline data benefited the control group |
| TBT |  | Mixed training vs Control intervention | Statistically significant | NA | 242 (1) | N/R |  |
| TBT |  | Mixed training vs Control intervention | Statistically significant | NA | 242 (1) | N/R | Three months follow up |
| Postural sway |  | Mixed training vs Control intervention | No effect | NA | 21 (1) | N/R |  |
| Balance (BBS, FRT, Four Square Step Test, TBT) |  | Mixed training vs Control intervention | Statistically significant | 22% | 596 (9) | High | Combined Balance scores |
| Balance (BBS, FRT, Four Square Step Test, TBT) |  | Mixed training vs Control intervention | Statistically significant | 0% | 140 (4) | N/R | Combined Balance scores, during usual care |
| Balance (BBS, FRT, Four Square Step Test, TBT) |  | Mixed training vs Control intervention | NO statistically significant differences between groups | 40% | 456 (5) | N/R | Combined Balance scores, after usual care |
| BBS |  | Cardiorespiratory vs resistance vs mixed training | Statistically significant | 31% | 723 (14) | N/R | Three different interventions compared |
| BBS | Vloothuis 2016 | Caregiver-mediated exercise in addition to usual care or instead of usual care vs Usual care | Statistically significant | 0% | 91 (2) | Low |  |
| PASS |  | Caregiver-mediated exercise in addition to usual care or instead of usual care vs Usual care | NO statistically significant differences between groups | NA | 48 (1) | Low |  |
| Balance (BBS, PASS) |  | Caregiver-mediated exercise in addition to usual care or instead of usual care vs Usual care | Statistically significant | 0% | 139 (3) | Low | Balance outcomes combined |
| BBS |  | Caregiver-mediated exercise in addition to usual care or instead of usual care vs Usual care | NO statistically significant differences between groups | NA | 40 (1) | Very low | Three months’ follow-up |
| BBS |  | Caregiver-mediated exercise in addition to usual care or instead of usual care vs Usual care | NO statistically significant differences between groups | NA | 40 (1) | Very low | Mean change from post intervention to three months’ follow-up |
| BBS |  | Caregiver-mediated exercise as only intervention (CME)-core vs Usual care | Statistically significant | 0% | 91 (2) | Moderate | Sensitivity analysis - caregiver-mediated exercise as only intervention (CME)-core |
| ***Non-Cochrane Reviews (non-CSRs)*** | | | | | | | |
| Balance (Shifting center of gravity movements, TUGT, BBS) | An 2011 | Aerobic exercises vs Control intervention | Positive effect on balance | N/R | 275 (4) | N/R |  |
| Balance (BBS) |  | Comprehensive exercises vs Control intervention | Effective in improving balance | N/R | 230 (3) | N/R |  |
| Balance (BBS, SRT, COP variability and total excursion) |  | Multisensory Training vs Control intervention | Both experimental and control groups improved balance, but there was no significant difference between the two groups | N/R | 145 (3) | N/R |  |
| TCT | Bank 2016 | Additional physiotherapy to standard physiotherapy vs Standard physiotherapy | NO statistically significant differences between groups | 22% | 263 (5) | N/R |  |
| TCT | Bank 2016 | Additional physiotherapy to standard physiotherapy vs Standard physiotherapy | NO statistically significant differences between groups | 0% | 228 (3) | N/R | Intervention duration equal to or less than 6 weeks |
| TCT |  | Additional physiotherapy to standard physiotherapy vs Standard physiotherapy | NO statistically significant differences between groups | 0% | 35 (2) | N/R | Intervention duration more than 6 weeks |
| TIS |  | Additional physiotherapy to standard physiotherapy vs Standard physiotherapy | Statistically significant | 75% | 106 (4) | N/R |  |
| TIS |  | Additional physiotherapy to standard physiotherapy vs Standard physiotherapy | NO statistically significant differences between groups | 0% | 73 (3) | N/R | Intervention duration equal to or less than 6 weeks |
| TIS |  | Additional physiotherapy to standard physiotherapy vs Standard physiotherapy | Statistically significant | NA | 33 (1) | N/R | Intervention duration more than 6 weeks |
| PASS-TC |  | Additional physiotherapy to standard physiotherapy vs Standard physiotherapy | Statistically significant improvement within-group, but no difference between groups | N/R | 33 (1) | N/R | Not included in meta-analysis |
| Symmetry Index |  | Additional physiotherapy to standard physiotherapy vs Standard physiotherapy | Statistically significant improvement within-group, but no difference between groups | N/R | 26 (1) | N/R | Not included in meta-analysis |
| BBS | Bonini-Rocha 2018 | Circuit-based exercise vs Conventional therapy or no intervention | NO statistically significant differences between groups | 0% | 174 (3) | N/R |  |
| mRT | Cabanas-Valdés 2013 | Trunk training exercises vs Conventional physiotherapy | Significantly improved | N/R | 59 (3) | N/R |  |
| TIS - Dynamic Sitting Balance subscale |  | Trunk training exercises vs Conventional physiotherapy | Significant improvement | N/R | 144 (5) | N/R |  |
| TIS - Static Sitting Balance subscale |  | Trunk training exercises vs Conventional physiotherapy | No favourable effect of the experimental training | N/R | 144 (5) | N/R |  |
| TIS - Coordination Subscale | Cabanas-Valdés 2013 | Trunk training exercises vs Conventional physiotherapy | 1 out of 5 studies (33 participants) showed no evidence of a significant difference in favour of the experimental group | N/R | 144 (5) | N/R |  |
| TIS [Total score] |  | Trunk training exercises vs Conventional physiotherapy | Significant difference in favour of the experimental intervention | N/R | 144 (5) | N/R |  |
| Static sitting balance (BWD) |  | Trunk training exercises vs Conventional physiotherapy | Any difference found between groups | N/R | 68 (2) | N/R |  |
| BWD when transferring from sitting to standing position |  | Trunk training exercises vs Conventional physiotherapy | Significantly improved in favour of experimental intervention | N/R | 31 (2) | N/R |  |
| BWD in a static standing position |  | Trunk training exercises vs Conventional physiotherapy | No remarkable difference between groups, but a significant improvement was observed in the experimental group | N/R | 35 (1) | N/R |  |
| Romberg Test |  | Trunk training exercises vs Conventional physiotherapy | No significant difference between groups | N/R | 33 (1) | N/R |  |
| Balance (BBS, Tinetti sub-scale, FTBS) |  | Trunk training exercises vs Conventional physiotherapy | Significant difference between groups | N/R | 33 (1) | N/R |  |
| Symmetry achieved in standing position |  | Trunk training exercises vs Conventional physiotherapy | No significant difference between groups | N/R | 40 (1) | NA |  |
| BBA |  | Trunk training exercises vs Conventional physiotherapy | Significant improvement only in the experimental group | N/R | 33 (1) | N/R |  |
| STS and Stand-to-sit (seconds) |  | Trunk training exercises vs Conventional physiotherapy | 1 out of 3 studies (30 participants) found a significant difference between groups | N/R | 93 (3) | N/R |  |
| BBS | Chen BL 2015 | Traditional Chinese exercises vs No intervention or other treatment | Statistically significant | 99% | 534 (6) | Very low |  |
| FMA - Balance subscale |  | Traditional Chinese exercises vs No intervention or other treatment | Statistically significant | N/R | 34 (1) | N/R | Not included in meta-analysis |
| Limit of Stability test |  | Traditional Chinese exercises vs No intervention or other treatment | Statistically significant | N/R | 136 (1) | N/R | Not included in meta-analysis |
| SOT |  | Traditional Chinese exercises vs No intervention or other treatment | Statistically significant | N/R | 136 (1) | N/R | Not included in meta-analysis |
| TUGT |  | Traditional Chinese exercises vs No intervention or other treatment | Statistically significant | N/R | 136 (1) | N/R | Not included in meta-analysis |
| SPPB |  | Traditional Chinese exercises vs No intervention or other treatment | Statistically significant | N/R | 145 (1) | N/R | Not included in meta-analysis |
| Balance (BBS, Romberg test, Standing on one leg) |  | Tai Chi vs Exercises focusing on improvement of balance | Significant improvements in the control group | N/R | 18 (1) | N/R | Not included in meta-analysis Significant improvements in the control group |
| BBS | Chen J 2015 | Telerehabilitation vs Conventional rehabilitation or no rehabilitation | NO statistically significant differences between groups | 0% | 54 (2) | N/R |  |
| BBS | Chen L 2016 | Sling exercise training vs Conventional rehabilitation treatments | Statistically significant | 75% | 204 (5) | N/R |  |
| BBS |  | Sling exercise training vs Conventional rehabilitation treatments | NO statistically significant differences between groups | NA | 80 (1) | N/R | Subgroup analysis (BBS scale compared after 2 weeks) |
| BBS |  | Sling exercise training vs Conventional rehabilitation treatments | Statistically significant | 82% | 104 (3) | N/R | Subgroup analysis (BBS scale compared after 4 weeks) |
| BBS |  | Sling exercise training vs Conventional rehabilitation treatments | Statistically significant | NA | 20 (1) | N/R | Subgroup analysis (BBS scale compared after 8 weeks) |
| BBS |  | Sling exercise training vs Conventional rehabilitation treatments | Statistically significant | 73% | 184 (4) | N/R | Sensitivity analysis |
| BBS |  | Sling exercise training vs Conventional rehabilitation treatments | NO statistically significant differences between groups | NA | 80 (1) | N/R | Sensitivity analysis: Subgroup analysis (BBS scale compared after 2weeks) |
| BBS | Chen L 2016 | Sling exercise training vs Conventional rehabilitation treatments | Statistically significant | 0% | 84 (2) | N/R | Sensitivity analysis: Subgroup analysis (BBS scale compared after 4 weeks) |
| BBS |  | Sling exercise training vs Conventional rehabilitation treatments | Statistically significant | NA | 20 (1) | N/R | Sensitivity analysis: Subgroup analysis (BBS scale compared after 8 weeks) |
| Balance (BBS, TUGT, Static and Dynamic balance assessed by force platform) | Chen Ling 2016 | Virtual reality vs Control intervention | 8 out of 9 studies (235 participants) showed significant improvements in balance outcomes | N/R | 265 (9) | N/R | BBS: 7 studies TUGT: 6 studies Static and Dynamic balance assessed by force platform: 5 studies |
| BBS | Cheok 2015 | Additional Wii vs Standard care | NO statistically significant differences between groups | 85% | 42 (2) | N/R |  |
| Postural sway AP eyes opened |  | Additional Wii vs Standard care | NO statistically significant differences between groups | 0% | 42 (2) | N/R |  |
| Postural sway AP eyes closed |  | Additional Wii vs Standard care | NO statistically significant differences between groups | 0% | 42 (2) | N/R |  |
| Postural sway ML eyes opened |  | Additional Wii vs Standard care | NO statistically significant differences between groups | 0% | 42 (2) | N/R |  |
| Postural sway ML eyes closed |  | Additional Wii vs Standard care | NO statistically significant differences between groups | 0% | 42 (2) | N/R |  |
| BBS | Corbetta 2015 | Virtual reality based rehabilitation vs Standard rehabilitation | Statistically significant | 0% | 130 (5) | N/R |  |
| BBS |  | Virtual reality based rehabilitation + standard rehabilitation vs Standard rehabilitation | NA | 97% | 86 (4) | N/R | No pooling due to heterogeneity |
| BBS | de Rooji 2016 | Virtual reality vs Conventional balance training | Statistically significant | 9% | 130 (5) | N/R | Time dose matched |
| BBS |  | Virtual reality vs Conventional balance training | NO statistically significant differences between groups | 98% | 44 (2) | N/R | Additional |
| TUGT |  | Virtual reality vs Conventional balance training | Statistically significant | 85% | 132 (6) | N/R | Time dose matched |
| TUGT | de Rooji 2016 | Virtual reality vs Conventional balance training | Statistically significant | NA | 20 (1) | N/R | Additional |
| TUGT |  | Virtual reality vs Conventional balance training | Statistically significant | 0% | 93 (4) | N/R | Time dose matched, after sensitivity analysis |
| Balance (BBS, pressure platform: stabilometry in ML and AP oscillation) | Dos Santos 2015 | Rehabilitation with Nintendo Wii + Conventional physiotherapy vs Conventional physiotherapy | Both experimental and control group showed significant improvements in BBS and ML oscillation Significant improvement in the experimental group in comparison to control group in AP oscillation | N/R | 12 (1) | N/R |  |
| Balance (BBS, TUGT, Pressure platform: posturography) |  | Rehabilitation with Nintendo Wii vs Rehabilitation without Nintendo Wii | Significant improvement in the experimental group in comparison to control group in BBS and TUGT outcomes No significant improvement in pressure platform (posturography) | N/R | 22 (1) | N/R |  |
| Balance (BBS, TUGT, pressure platform: stabilometry and baropodometry) |  | Rehabilitation with Nintendo Wii + Conventional physiotherapy vs Conventional physiotherapy | No significant intergroup improvement; Both experimental and control group showed significant improvements (intragroup) | N/R | 20 (1) | N/R |  |
| BBS | Ge 2017 | Traditional Chinese exercises vs Routine rehabilitation therapy or exercise | Statistically significant | 93% | 1272 (19) | N/R |  |
| BBS |  | Tai Chi exercise group vs Routine rehabilitation therapy or exercise | Statistically significant | 93% | 855 (14) | N/R | Subgroup meta-analysis regarding to different types of Traditional Chinese Exercises |
| BBS |  | Baduanjin exercise group vs Routine rehabilitation therapy or exercise | Statistically significant | 90% | 383 (4) | N/R | Subgroup meta-analysis regarding to different types of Traditional Chinese Exercises |
| BBS | Ge 2017 | Traditional Chinese exercises vs Routine rehabilitation therapy or exercise at Short-term (< 8 weeks) | Statistically significant | 92% | 749 (8) | N/R | Subgroup meta-analysis regarding to different lengths of intervention time |
| BBS |  | Traditional Chinese exercises vs Routine rehabilitation therapy or exercise at Mid-term (8-16 weeks) | Statistically significant | 95% | 507 (10) | N/R | Subgroup meta-analysis regarding to different lengths of intervention time |
| BBS |  | Traditional Chinese exercises vs Routine rehabilitation therapy or exercise at Long-term (> 16 weeks) | BBS scores were increased at the long-term intervention | NA | 16 (1) | N/R | Subgroup meta-analysis regarding to different lengths of intervention time |
| FMA - Balance subscale |  | Traditional Chinese exercises vs Routine rehabilitation therapy or exercise | NO statistically significant differences between groups | 78% | 114 (3) | N/R |  |
| TUGT |  | Traditional Chinese exercises vs Routine rehabilitation therapy or exercise | Statistically significant | 48% | 202 (4) | N/R |  |
| BBS |  | Traditional Chinese exercises vs Routine rehabilitation therapy or exercise | Statistically significant | 57% | 624 (10) | N/R | After sensitivity analysis |
| FMA - Balance subscale |  | Traditional Chinese exercises vs Routine rehabilitation therapy or exercise | NO statistically significant differences between groups | N/R | 74 (2) | N/R | After sensitivity analysis |
| BBS |  | Tai Chi exercise group vs Routine rehabilitation therapy or exercise | Statistically significant | 0% | 423 (8) | N/R | After sensitivity analysis |
| BBS |  | Baduanjin exercise group vs Routine rehabilitation therapy or exercise | Statistically significant | 60% | 112 (2) | N/R | After sensitivity analysis |
| BBS |  | Traditional Chinese exercises vs Routine rehabilitation therapy or exercise at Short-term (< 8 weeks) | Statistically significant | 66% | 411 (4) | N/R | After sensitivity analysis |
| BBS |  | Traditional Chinese exercises vs Routine rehabilitation therapy or exercise at Mid-term (8-16 weeks) | Statistically significant | 61% | 311 (7) | N/R | After sensitivity analysis |
| RMI | Hammer 2008 | Standing with the Oswestry frame vs standard care | Experimental intervention not more efficient than control intervention  Both groups made clinically relevant improvements | N/R | 115 (1) | N/R | Acute phase |
| Postural sway | Hammer 2008 | Lateral weight shifting in standing + standard care vs Standard care | No advantage in experimental group in comparison to control group  Improvements within both groups | N/R | 31 (1) | N/R | Acute phase |
| Balance (BBS, MAS) |  | Bobath vs Orthopaedic treatment | Both statistically significant and clinically relevant improvements within and between groups  Experimental group showed greater improvements between groups | N/R | 44 (1) | N/R | Acute phase |
| BBS |  | Therapist-supervised, moderately intense home training programmes vs standard care | Relevant improvements within and between groups | N/R | 112 (2) | N/R | Sub-acute phase |
| Balance (TUGT, ST) |  | Balance exercises vs upper extremity exercises | One of three studies showed considerable improvements within and between groups | N/R | 133 (3) | N/R | Sub-acute phase |
| STREAM (basic mobility part) |  | "Problem-oriented willed-movement therapy" vs neurodevelopmental treatment | Both statistically significant and clinically relevant improvements within and between groups | N/R | 47 (1) | N/R | Sub-acute phase |
| Postural sway |  | Perceptional learning exercise + Rehabilitation vs Rehabilitation only | Statistically significant changes between groups | N/R | 26 (1) | N/R | Sub-acute phase |
| Balance (BBS, TUGT, ST) |  | Balance exercises vs upper extremity exercises | Within group improvements but conflicting results regarding between group differences | N/R | 68 (2) | N/R | Late phase |
| Balance (BBS, TUGT, SRT) |  | One balance exercise vs One different balance exercise | Several improvements within and between groups | N/R | 42 (1) | N/R | Late phase |
| Balance (Posturography) |  | Balance programme with visual cues vs Balance programme without visual cues | Statistically significant changes between groups | N/R | 20 (1) | N/R | Late phase |
| Balance (PASS [total], Pass - Static subscale, Pass - Dynamic subscale) | Hancock 2012 | Lower limb reciprocal pedalling exercise vs Routine therapy | Beneficial effects on balance | N/R | 24 (1) | N/R | Immediate outcome |
| Balance (PASS [total], Pass - Static subscale, Pass - Dynamic subscale) | Hancock 2012 | Lower limb reciprocal pedalling exercise vs Routine therapy | Beneficial effects on balance | N/R | 24 (1) | N/R | Follow up (six-weeks) |
| Balance (BBS, Get Up and Go) |  | Lower limb reciprocal pedalling exercise vs Home-based program of stretching exercises | Beneficial effects on balance | N/R | 38 (1) | N/R | Immediate outcome |
| Balance (BBS, Get Up and Go) |  | Lower limb reciprocal pedalling exercise vs Home-based program of stretching exercises | Beneficial effects on balance | N/R | 38 (1) | N/R | Follow up (eight-weeks) |
| BBS | Iruthayarajah 2017 | Virtual reality vs Alternative rehabilitation therapy | Statistically significant | 3% | 274 (12) | N/R |  |
| BBS |  | Nintendo Wii Fit balance board vs Alternative rehabilitation therapy | NO statistically significant differences between groups | 0% | 87 (4) | N/R | Sub-group analysis |
| BBS |  | Treadmill training with VR vs Alternative rehabilitation therapy | NO statistically significant differences between groups | 0% | 72 (3) | N/R | Sub-group analysis |
| BBS |  | Postural VR interventions vs Alternative rehabilitation therapy | Statistically significant | 53% | 115 (5) | N/R | Sub-group analysis |
| TUGT |  | Virtual reality vs Alternative rehabilitation therapy | Statistically significant | 0% | 312 (13) | N/R |  |
| TUGT |  | Nintendo Wii Fit balance board vs Alternative rehabilitation therapy | NO statistically significant differences between groups | 0% | 127 (5) | N/R | Sub-group analysis |
| TUGT |  | Treadmill training with VR vs Alternative rehabilitation therapy | Statistically significant | 0% | 123 (5) | N/R | Sub-group analysis |
| TUGT |  | Postural VR interventions vs Alternative rehabilitation therapy | N/A | 67% | 62 (3) | N/R | Sub-group analysis |
| FRT |  | Nintendo Wii Fit balance board vs Alternative rehabilitation therapy | Significant between-group difference reported by one study | N/R | 52 (2) | N/R | Not included in meta-analysis |
| 10MWT |  | Virtual reality vs Alternative rehabilitation therapy | Significant between-groups differences found in two studies | N/R | 77 (3) | N/R | Not included in meta-analysis |
| ART | Iruthayarajah 2017 | Nintendo Wii Fit balance board vs Alternative rehabilitation therapy | Significant improvements | N/R | 17 (1) | N/R | Not included in meta-analysis |
| Balance (6MWT, 3MWT) |  | Virtual reality vs Alternative rehabilitation therapy | No significant between-group differences | N/R | 28 (1) | N/R | Not included in meta-analysis |
| Balance (1MWT, TST, 30SST) |  | Virtual reality vs Alternative rehabilitation therapy | No significant between-group differences | N/R | 17 (1) | N/R | Not included in meta-analysis |
| POMA |  | Virtual reality vs Alternative rehabilitation therapy | No significant between-group differences | N/R | 49 (2) | N/R | Not included in meta-analysis |
| BBA |  | Virtual reality vs Alternative rehabilitation therapy | No significant between-group differences | N/R | 20 (1) | N/R | Not included in meta-analysis |
| COP path lengths and oscillations |  | Virtual reality vs Alternative rehabilitation therapy | Significant between-group difference reported by one study | N/R | 58 (3) | N/R | Not included in meta-analysis |
| Limits of stability |  | Nintendo Wii Fit balance board vs Alternative rehabilitation therapy | Significant between-group difference | N/R | 40 (1) | N/R | Not included in meta-analysis |
| Postural sway path lengths and velocities |  | Virtual reality vs Alternative rehabilitation therapy | Significant between-group difference reported by one study | N/R | 69 (3) | N/R | Not included in meta-analysis |
| Stability Index |  | Nintendo Wii Fit balance board vs Alternative rehabilitation therapy | Significant between-group difference | N/R | 28 (1) | N/R | Not included in meta-analysis |
| BWD |  | Nintendo Wii Fit balance board vs Alternative rehabilitation therapy | Significant between-group differences | N/R | 68 (2) | N/R | Not included in meta-analysis |
| Symmetry Index |  | VR treadmill training vs Alternative rehabilitation therapy | No significant between-group differences | N/R | 14 (1) | N/R | Not included in meta-analysis |
| Balance (Tinetti test, TIS - Dynamic Sitting Balance subscale, FTBS, BBS) | Ko 2014 | Lumbar stabilization exercises + Conventional physical therapy vs Arm therapy + Conventional physical therapy | Significant improvements in the experimental group | N/R | 33 (1) | N/R | Acute stroke |
| TIS - Dynamic Sitting Balance subscale |  | Lumbar stabilization exercises + Conventional physical therapy vs Conventional physical therapy | Significant improvements in the experimental group | N/R | 33 (1) | N/R | Acute stroke |
| Balance (TIS - Dynamic Sitting Balance subscale, BBA) | Ko 2014 | Lumbar stabilization exercises on unstable surfaces + Conventional physical therapy vs Lumbar stabilization exercises on stable surfaces + Conventional physical therapy | Significant improvements in the experimental group | N/R | 30 (1) | N/R | Acute stroke |
| FRT |  | Lumbar stabilization exercises vs General exercise intervention | Significant improvements in the experimental group | N/R | 40 (1) | N/R | Chronic stroke |
| TUGT |  | Lumbar stabilization exercises vs General exercise intervention | Significant improvements in the experimental group | N/R | 16 (1) | N/R | Chronic stroke |
| Balance (SA, SP and TIS) |  | Lumbar stabilization exercises on unstable surfaces vs Lumbar stabilization exercises on stable surfaces | Significant improvements in the experimental group | N/R | 16 (1) | N/R | Chronic stroke |
| BWD | Kollen 2009 | Bobath Concept vs Control intervention | Based on van Tulder's Best Evidence Synthesis, we suggest that there is **limited evidence** for the superiority of Bobath Concept. | N/R | 60 (2) | N/R |  |
| MAS |  | Bobath Concept vs Control intervention | Based on van Tulder's Best Evidence Synthesis, we suggest that there is **limited evidence** for the superiority of Bobath Concept. | N/R | 120 (1) | N/R |  |
| BBS |  | Bobath Concept vs Control intervention | Based on van Tulder's Best Evidence Synthesis, we suggest that there is **limited evidence** for the superiority of Bobath Concept. | N/R | 44 (1) | N/R |  |
| Standing balance (BBS, BWD, postural sway during sitting and standing) | Langhorne 2009 | Mixed approaches vs Control intervention | N/R | N/R | 127 (3) | N/R | Semi-quantitative Clinical Evidence classification of effectiveness: unknown effectiveness |
| Standing balance (BBS, BWD, postural sway during sitting and standing) |  | Motor approaches vs Control intervention | N/R | N/R | 91 (1) | N/R | Semi-quantitative Clinical Evidence classification of effectiveness: unknown effectiveness |
| Standing balance (BBS, BWD, postural sway during sitting and standing) | Langhorne 2009 | Neurophysiological approaches vs Control intervention | N/R | N/R | 15 (1) | N/R | Semi-quantitative Clinical Evidence classification of effectiveness: unknown effectiveness |
| BWD |  | Biofeedback using a force plate vs Control intervention | Pattern of improvement | N/R | 161 (4) | N/R | Semi-quantitative Clinical Evidence classification of effectiveness: likely to be beneficial |
| Postural sway during sitting and standing |  | Biofeedback using a force plate vs Control intervention | NO statistically significant differences between groups | N/R | 71 (3) | N/R | Semi-quantitative Clinical Evidence classification of effectiveness: unknown effectiveness |
| Standing balance (BBS, BWD, postural sway during sitting and standing) |  | Moving platform vs Control intervention | Positive intervention | N/R | 40 (2) | N/R | Semi-quantitative Clinical Evidence classification of effectiveness: likely to be beneficial |
| Standing balance (BBS, BWD, postural sway during sitting and standing) |  | Repetitive task training vs Control intervention | NO statistically significant differences between groups | N/R | 132 (3) | N/R | Semi-quantitative Clinical Evidence classification of effectiveness: unknown effectiveness |
| BBS | Li 2016 | Virtual reality vs Usual care, placebo control intervention, or any other exercise intervention without virtual reality | Statistically significant | 0% | 170 (8) | N/R |  |
| FRT |  | Virtual reality vs Usual care, placebo control intervention, or any other exercise intervention without virtual reality | NO statistically significant differences between groups | 60% | 47 (2) | N/R |  |
| ABC Scale | Li 2016 | Virtual reality vs Usual care, placebo control intervention, or any other exercise intervention without virtual reality | NO statistically significant differences between groups | 77% | 41 (2) | N/R |  |
| TUGT |  | Virtual reality vs Usual care, placebo control intervention, or any other exercise intervention without virtual reality | Statistically significant | 24% | 214 (8) | N/R |  |
| Sway velocity |  | Virtual reality vs Usual care, placebo control intervention, or any other exercise intervention without virtual reality | NO statistically significant differences between groups | 0% | 69 (3) | N/R |  |
| BWD |  | Virtual reality vs Usual care, placebo control intervention, or any other exercise intervention without virtual reality | NO statistically significant differences between groups | 0% | 74 (3) | N/R |  |
| BBS |  | Virtual reality (< 6 months) vs Virtual reality (> 6 months) | NO statistically significant differences between groups | N/R | 170 (8) | N/R | Sub-group analysis (time post-stroke) |
| TUGT |  | Virtual reality (< 6 months) vs Virtual reality (> 6 months) | NO statistically significant differences between groups | N/R | 214 (8) | N/R | Sub-group analysis (time post-stroke) |
| BBS |  | N/A | NO statistically significant differences between groups | N/R | N/A | N/R | Sub-group analysis (intervention type) |
| TUGT |  | N/A | NO statistically significant differences between groups | N/R | N/A | N/R | Sub-group analysis (intervention type) |
| TUGT | Lin 2018 | TENS vs TENS Placebo | NO statistically significant differences between groups | 38% | 77 (2) | N/R |  |
| Postural sway velocity, EO |  | TENS vs TENS Placebo | Statistically significant | 0% | 29 (1) | N/R |  |
| Postural sway velocity, EO in AP |  | TENS vs TENS Placebo | Statistically significant | NA | 29 (1) | N/R | Anteroposterior direction |
| Postural sway velocity, EO in ML |  | TENS vs TENS Placebo | Statistically significant | NA | 29 (1) | N/R | Mediolateral direction |
| Postural sway velocity, EC |  | TENS vs TENS Placebo | Statistically significant | 0% | 29 (1) | N/R |  |
| Postural sway velocity, EC in AP | Lin 2018 | TENS vs TENS Placebo | Statistically significant | NA | 29 (1) | N/R | Anteroposterior direction |
| Postural sway velocity, EC in ML |  | TENS vs TENS Placebo | Statistically significant | NA | 29 (1) | N/R | Mediolateral direction |
| BBS | Lu 2015 | Whole Body Vibration vs No intervention, the same exercise without vibration or placebo vibrating platform | NO statistically significant differences between groups | 0% | 113 (2) | N/R |  |
| Balance (BBS, Postural Control and Balance Test) | Lubetzky-Vilnay 2010 | Balance training vs Control intervention | Both experimental and control groups demonstrated significant improvements in the balance test scores with no significant between-groups differences | N/R | 272 (5) | N/R | Acute stage |
| BBS |  | Motor relearning program vs Control Intervention | Significantly larger improvements in the experimental group in comparison to control group | N/R | 52 (1) | N/R | Acute stage |
| Balance (BBS, force platform measures of balance index, dynamic limits of stability) |  | Balance training session with a force platform with visual feedback + Conventional stroke rehabilitation vs Conventional stroke rehabilitation | Significant improvements on both functional and instrumented measures | N/R | 40 (1) | N/R | Sub-acute stage |
| Brunnstrom stage |  | Balance training session with a portable balance trainer + Conventional stroke rehabilitation vs Conventional stroke rehabilitation | Statistically significant improvements | N/R | 41(1) | N/R | Sub-acute stage |
| BBS |  | Outpatient multisensorial program vs Control intervention | Statistically significant improvements | N/R | 68 (1) | N/R | Sub-acute stage |
| Balance (BBS, FMA - Balance subscale, Balance Index on the Kinesthetic Ability Trainer, COP displacement, ABC Scale, FES, DGI) | Lubetzky-Vilnay 2010 | Balance rehabilitation in group or one-on-one session vs Control intervention | Important changes in balance performance | N/R | 252 (11) | N/R | Chronic stage: "One-on-One" interventions (4 studies, 75 participants) or Group therapy (7 studies, 177 participants) |
| BBS | Luque-Moreno 2015 | Virtual reality vs Alternative intervention or no intervention | 3 out of 4 trial (71 participants) concluded significant improvement results in the VR group for balance | N/R | 99 (4) | N/R |  |
| Balance (Upright equilibrium index, Tinetti Scale) | Sorinola 2014 | Additional trunk exercises vs Conventional rehabilitation | Statistically significant | 39% | 53 (2) | N/R |  |
| BBS | Stoller 2012 | Early cardiovascular exercise vs Usual care | Balance improved | NA | 100 (1) | N/R |  |
| Balance (BBS, FRT) |  | Early cardiovascular exercise vs Usual care | No improvements | NA | 163 (3) | N/R |  |
| BBS | Swinnen 2014 | Robot-assisted gait training vs Other gait rehabilitation methods | No significant differences in BBS scores between the intervention and control groups | N/R | 222 (5) | N/R |  |
| Tinetti Score |  | Robot-assisted gait training vs Other gait rehabilitation methods | Training not significantly improve the Tinetti score in the intervention group as compared with the control group | N/R | 20 (1) | N/R |  |
| Postural Sway tests |  | Robot-assisted gait training vs Other gait rehabilitation methods | No significant differences were found between the groups | N/R | 45 (1) | N/R |  |
| Tinetti Score | Swinnen 2014 | Robot-assisted gait training vs Other gait rehabilitation methods | Significant improvements | N/R | 69 (1) | N/R | Within group differences (no control group) |
| Balance (BBS, TUGT, Romberg test) |  | Robot-assisted gait training vs Other gait rehabilitation methods | All patients improved their scores after training | N/R | 3 (1) | N/R | Within group differences (no control group) |
| BBS | Tally 2017 | Isolated treadmill training vs Conventional physical therapy treatments | Statistically significant improvements pre- to post- intervention | N/R | 80 (2) | N/R | Pre- to post- intervention |
| BBS |  | Treadmill training (TT) + FES on gluteus medius and tibialis anterior (TA) vs Isolated TT or Isolated FES on the TA | Marked improvements | N/R | 31 (1) | N/R |  |
| BBS |  | Treadmill training with tilt sensor FES and WalkAide system vs Treadmill training with tilt sensor FES and WalkAide system in the off position | Clinically relevant improvements in both placebo and experimental groups | N/R | 30 (1) | N/R | A larger effect noted in the experimental vs placebo intervention |
| Limits of stability |  | Treadmill training with eyes closed vs Treadmill training with eyes opened | Beneficial in balance measure | N/R | 37 (1) | N/R |  |
| BBS |  | Nordic Treadmill training vs Treadmill training | Statistically significant differences between pre- and post-intervention in both groups | N/R | 30 (1) | N/R | Pre- to post- intervention |
| BBS |  | Rotational treadmill training vs Conventional treadmill training | Significant advancements in both groups | N/R | 30 (1) | N/R | Significant difference in favor of experimental group in intergroup comparison |
| Postural sway velocity |  | Cognitive-motor dual task while treadmill training vs Single task while treadmill training | Statistically significant improvements in medial-lateral sway in both groups | N/R | 37 (1) | N/R | Significant difference in favor of experimental group in intergroup comparison |
| Balance self-efficacy (ABC Scale, FES-International and FES-Swedish versio) | Tang 2015 | More intense physical exercise-based interventions vs Less intensive programs | Statistically significant | 73% | 627 (15) | N/R |  |
| Balance self-efficacy (ABC Scale, FES-International and FES-Swedish version) | Tang 2015 | More intense physical exercise-based interventions vs Less intensive programs | Statistically significant | N/R | 582 (13) | N/R | Sensitivity analysis: Huijbregts 2008 (24) and Pang 2010 (21) removed because NRCT |
| ABC Scale |  | More intense physical exercise-based interventions vs Less intensive programs | Statistically significant | N/R | 545 (12) | N/R | Studies that used the ABC scale |
| Balance self-efficacy (ABC Scale, FES-International and FES-Swedish version) |  | More intense physical exercise-based interventions vs Less intensive programs | NO statistically significant differences between groups | 78% | 347 (8) | N/R | Follow up |
| Balance self-efficacy (ABC Scale, FES-International and FES-Swedish version) |  | More intense physical exercise-based interventions vs Less intensive programs | Statistically significant | 0% | 593 (14) | N/R | Sensitivity analysis: Holmgren 2010 (34) removed as an outlier study |
| Balance self-efficacy (ABC Scale, FES-International and FES-Swedish version) |  | More intense physical exercise-based interventions vs Less intensive programs | NO statistically significant differences between groups | 0% | 313 (7) | N/R | Sensitivity analysis of follow up: Holmgren 2010 (34) removed as an outlier study |
| Balance self-efficacy (ABC Scale, FES-International and FES-Swedish version) |  | Motor imagery interventions vs Control intervention | No difference between groups | N/R | 102 (4) | N/R | Not included in meta-analysis |
| BBS | Tyson 2013 | Walking with Ankle-Foot Orthosis vs Walking without Ankle-Foot Orthosis | No significant effect in either the group of acute or chronic stroke | NA | 103 (1) | N/R | Crossover design Wang 2005 (103) not entered into the meta-analysis for BBS outcome because it did not report any SD data |
| BBS |  | Walking with Ankle-Foot Orthosis vs Walking without Ankle-Foot Orthosis | Significant effect | NA | 20 (1) | N/R | Crossover design |
| BWD while standing |  | Walking with Ankle-Foot Orthosis vs Walking without Ankle-Foot Orthosis | Statistically significant | 0% | 183 (5) | N/R | Crossover design |
| Postural sway |  | Walking with Ankle-Foot Orthosis vs Walking without Ankle-Foot Orthosis | NO statistically significant differences between groups | 0% | 163 (4) | N/R | Crossover design |
| Sitting balance | Van Criekinge 2018 | Trunk rehabilitation using unstable surfaces vs Trunk rehabilitation using stable surfaces, whether or not combined with conventional therapy | Statistically significant | 63% | 100 (4) | N/R | Partial meta-analysis on sitting balance |
| Standing balance |  | Trunk rehabilitation using unstable surfaces vs Trunk rehabilitation using stable surfaces, whether or not combined with conventional therapy | NO statistically significant differences between groups | 91% | 160 (6) | N/R | Partial meta-analysis on standing balance |
| BBS | van Duijnhoven 2016 | Exercise therapy vs Usual care | Significant SES | 52% | 985 (28) | N/R |  |
| FRT |  | Exercise therapy vs Usual care | Significant SES | 74% | 153 (5) | N/R |  |
| SOT |  | Exercise therapy vs Usual care | Significant SES | 0% | 173 (4) | N/R |  |
| Postural sway velocities (AP direction) |  | Exercise therapy vs Usual care | Nonsignificant SES | 74% | 89 (3) | N/R |  |
| Postural sway velocities (ML direction) | van Duijnhoven 2016 | Exercise therapy vs Usual care | Nonsignificant SES | 91% | 89 (3) | N/R |  |
| BBS |  | Exercise therapy vs Usual care | Significant SES | 0% | 338 (8) | N/R | Follow up |
| SOT |  | Exercise therapy vs Usual care | Significant SES | 0% | 151 (3) | N/R | Follow up |
| BBS |  | Balance and/or functional weight-shifting training vs Usual care | Significant SES | 52% | 235 (8) | N/R | Subgroup analysis for intervention type |
| BBS |  | Gait training vs Usual care | Significant SES | 21% | 290 (10) | N/R | Subgroup analysis for intervention type |
| BBS |  | Multisensory training vs Usual care | Nonsignificant SES | 22% | 153 (4) | N/R | Subgroup analysis for intervention type |
| BBS |  | High-intensity aerobic training vs Usual care | Nonsignificant SES | 0% | 246 (4) | N/R | Subgroup analysis for intervention type |
| Postural symmetry sit-to-stand | Van Peppen 2004 | Training sit-to-stand transfers and vice versa vs Control intervention | Statistically significant SES | Homogeneous | 128 (4) | N/R |  |
| Postural symmetry stand-to-sit |  | Training sit-to-stand transfers and vice versa vs Control intervention | Statistically significant SES | Homogeneous | 96 (2) | N/R |  |
| Time needed to stand-up |  | Training sit-to-stand transfers and vice versa vs Control intervention | Statistically significant SES | Homogeneous | 84 (2) | N/R |  |
| Time needed to sit-down |  | Training sit-to-stand transfers and vice versa vs Control intervention | Significant SES | Heterogeneous | 84 (2) | N/R |  |
| Postural sway/symmetry |  | Training standing balance vs Control intervention | Statistically significant | N/R | 126 (5) | N/R |  |
| BBS |  | Training standing balance vs Control intervention | NO statistically significant differences between groups | N/R | 59 (3) | N/R |  |
| TUGT |  | Training standing balance vs Control intervention | Statistically significant negative SES | Heterogeneous | 59 (3) | N/R |  |
| BBS |  | Body weight supported treadmill training vs Control intervention | NO statistically significant differences between groups | N/R | 145 (2) | N/R |  |
| BBS |  | Exercise training with weighted garments vs No weighted garments | No statistically significant effects | N/R | 24 (1) | N/R |  |
| BWD while bilateral standing | Van Peppen 2006 | Bilateral standing with visual feedback therapy vs Conventional therapy | Non-significant SES | Homogeneous | 75 (3) | N/R |  |
| BWD while bilateral standing | Van Peppen 2006 | Bilateral standing with visual feedback therapy vs Conventional therapy | Non-significant SES | N/R | 41 (2) | N/R | Sensitivity analysis: Winstein 1989 (34) excluded because being NRCT |
| Postural sway in bilateral standing with eyes open |  | Bilateral standing with visual feedback therapy vs Conventional therapy | Non-significant SES | Heterogeneous | 148 (5) | N/R |  |
| Postural sway in bilateral standing with eyes open |  | Bilateral standing with visual feedback therapy vs Conventional therapy | Non-significant SES | N/R | 114 (4) | N/R | Sensitivity analysis: Winstein 1989 (34) excluded because being NRCT |
| Postural sway in bilateral standing with eyes closed |  | Bilateral standing with visual feedback therapy vs Conventional therapy | Non-significant SES | Homogeneous | 73 (2) | N/R |  |
| BBS |  | Bilateral standing with visual feedback therapy vs Conventional therapy | Non-significant SES | Homogeneous | 45 (2) | N/R |  |
| TUGT |  | Bilateral standing with visual feedback therapy vs Conventional therapy | Non-significant SES | Homogeneous | 44 (2) | N/R |  |
| Symmetry while sitting | Veerbeek 2014 | Sitting balance training vs Control intervention | Nonsignificant SES | 26% | 59 (2) | N/R |  |
| Symmetry while standing |  | Sitting balance training vs Control intervention | Nonsignificant SES | 38% | 59 (2) | N/R |  |
| Sitting balance (reach distance, sitting equilibrium test) |  | Sitting balance training vs Control intervention | Nonsignificant SES | 90% | 70 (4) | N/R |  |
| Sitting balance (reach distance, sitting equilibrium test) |  | Sitting balance training (reaching beyond arm’s length while sitting) vs Control intervention | Significant heterogeneous positive SES | 77% | 50 (3) | N/R |  |
| Body weight distribution (BWD sitting, BWD rising, BWD sitting down) |  | Sit-to-stand training vs Control intervention | Nonsignificant SES | 77% | 13 (1) | N/R |  |
| STS | Veerbeek 2014 | Sit-to-stand training vs Control intervention | Nonsignificant SES | 0% | 50 (2) | N/R |  |
| Postural sway |  | Standing balance training without biofeedback vs Control intervention | Nonsignificant SES | 33% | 42 (2) | N/R |  |
| Balance (BBS, MAS - Sitting subscale) |  | Standing balance training without biofeedback vs Control intervention | Nonsignificant SES | 0% | 149 (2) | N/R |  |
| Sit-to-Stand (MAS - Sit to Stand Subscale) |  | Standing balance training without biofeedback vs Control intervention | Nonsignificant SES | 0% | 149 (2) | N/R |  |
| Postural sway |  | Standing balance training with biofeedback - force and position feedback vs Control intervention | Significant homogeneous positive SES | 0% | 151 (7) | N/R |  |
| Postural sway |  | Standing balance training with biofeedback - force and position feedback vs Control intervention | Significant homogeneous positive SES | 0% | 30 (1) | N/R | Chronic phase (> 6 months) |
| Postural sway |  | Standing balance training with biofeedback - force and position feedback vs Control intervention | Nonsignificant SES | 0% | 121 (6) | N/R | Early rehabilitation phase (24 h - 3 months) |
| Balance (BBS, FMA balance, PASS, SST affected leg, SST nonaffected leg) |  | Standing balance training with biofeedback - force and position feedback vs Control intervention | Nonsignificant SES | 0% | 186 (6) | N/R |  |
| Balance (BBA, BBS, FRT, LRT, static standing, SRT, ST, TIS) |  | Balance training during various activities vs Control intervention | Significant heterogeneous positive SES | 51% | 397 (9) | N/R |  |
| Balance (BBS, FMA balance) | Veerbeek 2014 | Body-weight supported treadmill training vs Control intervention | Nonsignificant SES | 92% | 540 (9) | N/R |  |
| Balance (BBS, Dynamic balance, SST affected leg, Static balance, Tinetti balance, ST) |  | Electromechanical-assisted gait training without functional electrostimulation vs Control intervention | Nonsignificant SES | 84% | 320 (9) | N/R |  |
| Balance (BBS, Dynamic balance, SST affected leg, Static balance, Tinetti balance, ST) |  | Electromechanical-assisted gait training without functional electrostimulation vs Control intervention | Significant homogeneous positive SES | 0% | 123 (4) | N/R | Early rehabilitation phase (24 h - 3 months) |
| Balance (BBS, Dynamic balance, SST affected leg, Static balance, Tinetti balance, ST) |  | Electromechanical-assisted gait training without functional electrostimulation vs Control intervention | Significant negative SES | 0% | 63 (1) | N/R | Late rehabilitation phase (3-6 months) |
| Balance (BBS, Dynamic balance, SST affected leg, Static balance, Tinetti balance, ST) |  | Electromechanical-assisted gait training without functional electrostimulation vs Control intervention | Nonsignificant SES | 0% | 134 (4) | N/R | Chronic phase (> 6 months) |
| Balance (BBS, Dynamic balance, Static balance) |  | Electromechanical-assisted gait training with functional electrostimulation vs Control intervention | Significant homogeneous positive SES | 44% | 102 (2) | N/R | Early rehabilitation phase (24 h - 3 months) |
| Balance (BBS, Balance) | Veerbeek 2014 | Speed dependent treadmill training (without body-weight support) vs Control intervention | Nonsignificant SES | 0% | 44 (2) | N/R |  |
| Balance (BBS, Dynamic balance, Static balance) |  | Overground walking vs Control intervention | Nonsignificant SES | 86% | 262 (5) | N/R |  |
| Balance (BBS, Dynamic balance, Static balance) |  | Overground walking vs Control intervention | Significant positive SES | 0% | 63 (1) | N/R | Late rehabilitation phase (3-6 months) |
| Balance (BBS, Dynamic balance, Static balance) |  | Overground walking vs Control intervention | Nonsignificant SES | 0% | 199 (4) | N/R | Chronic phase (> 6 months) |
| Balance confidence (ABC Scale) |  | Community walking vs Control intervention | Nonsignificant SES | 0% | 55 (2) | N/R |  |
| Balance (BBS, FRT, ST) |  | Circuit class training vs Control intervention | Significant homogeneous positive SES | 0% | 298 (5) | N/R |  |
| Balance (BBS) |  | Water-based exercises vs Control intervention | Nonsignificant SES | 67% | 32 (2) | N/R |  |
| Balance (BBS) |  | Interventions for somatosensory functions of the paretic leg vs Control intervention | Nonsignificant SES | 85% | 54 (2) | N/R |  |
| Balance (BBS, balance) |  | Neuromuscular stimulation vs Control intervention | Nonsignificant SES | 40% | 125 (4) | N/R |  |
| SA | Wang 2015 | Cognitive motor interference vs Single-task exercise or no treatment | Statistically significant | 88% | 270 (4) | N/R |  |
| SA |  | Cognitive motor interference vs Single-task exercise or no treatment | NO statistically significant differences between groups | 90% | 185 (4) | N/R | COP SA under eye open |
| SA |  | Cognitive motor interference vs Single-task exercise or no treatment | Statistically significant | NA | 85 (1) | N/R | COP SA under eye close |
| SD | Wang 2015 | Cognitive motor interference vs Single-task exercise or no treatment | NO statistically significant differences between groups | 81% | 276 (4) | N/R |  |
| BBS |  | Cognitive motor interference vs Single-task exercise or no treatment | Statistically significant | 50% | 96 (4) | N/R |  |
| TUGT |  | Cognitive motor interference vs Single-task exercise or no treatment | NO statistically significant differences between groups | 32% | 57 (3) | N/R |  |
| ABC Scale |  | Cognitive motor interference vs Single-task exercise or no treatment | NO statistically significant differences between groups | 77% | 41 (2) | N/R |  |
| ST | Wevers 2009 | Circuit class training vs Upper extremity training or no rehabilitation training | Nonsignificant homogeneous SES | 0% | 87 (3) | N/R |  |
| BBS |  | Circuit class training vs Upper extremity training | Nonsignificant homogeneous SES | 0% | 154 (2) | N/R |  |
| BBS | Wist 2016 | Strengthening of the lower limbs vs Control intervention | NO statistically significant differences between groups | 83% | 207 (4) | N/R |  |
| BBS |  | Progressive resistance training vs Control intervention | Significant improvements in favour of the experimental group | N/R | 30 (1) | N/R | Subgroup analysis based on intervention type |
| BBS |  | Aerobic exercise vs Control intervention | Did not demonstrate improvement in favour of one or other group | N/R | 133 (1) | N/R | Subgroup analysis based on intervention type |
| BBS |  | Task-specific training vs Control intervention | Did not demonstrate improvement in favour of one or other group | N/R | 32 (1) | N/R | Subgroup analysis based on intervention type |
| BBS |  | FES vs Control intervention | Marked improvements in favour of the control group | N/R | 12 (1) | N/R | Subgroup analysis based on intervention type |
| TUGT |  | Strengthening of the lower limbs vs Control intervention | Statistically significant | 72% | 92 (3) | N/R |  |
| TUGT |  | Strengthening of the lower limbs vs Control intervention | NO statistically significant differences between groups | N/R | 18 (1) | N/R | Long-term analysis (at 5 months) |
| TUGT |  | Strengthening of the lower limbs vs Control intervention | NO statistically significant differences between groups | N/R | 18 (1) | N/R | Long-term analysis (at 4 years) |
| BBS | Yang 2015 | Whole Body Vibration vs No intervention, sham vibration, usual care or exercise therapy on music | NO statistically significant differences between groups | 0% | 186 (4) | N/R |  |

10MWT= 10-Meter Walking Test; 1MWT= 1-Minute Walking Test; 30SST= 30-Second Sit to Stand Test; 3MWT= 3-Meter Walking Test; 6MWT= 6-Minute Walking Test; ABC Scale= Activities Based Confidence Scale; AP= Anteroposterior; ART= Anterior Reach Test; BBA= Brunel Balance Assessment; BBS= Berg Performance Scale; BPM= Balance Performance Monitor; BWD= Body Weight Distribution; COP= Centre of Pressure; DGI= Dynamic Gait Index; EC= Eyes closed; EO= Eyes opened; FES= Falls Efficacy Scale; FMA= Fugl-Meyer Assessment; FRT= Functional Reach Test; FTBS= Four Test Balance Scale; MAS= Motor Assessment Scale; ML= Mediolateral; mRT= Modified Reach Test; N/R= Not reported; NA= Not applicable; PASS= Postural Assessment Scale for Stroke patients; PASS-TC= Postural assessment scale for stroke patients - Trunk Control; POMA= Tinetti Performance Oriented Mobility Assessment; RMI= Rivermead Mobility Index; SA= Sway Area of the COP; SD= Sway Distance of the COP; SOT= Sensory Organization Test; SP= Sway Path of the COP; SPPB= Short Physical Performance Battery; SRT= Step Reaction Time; SST= Single Support Time; ST= Step Test; STREAM= Stroke Rehabilitation Assessment of Movement; STS= Sit-to-stand; TBT= Timed Balance Test; TCT= Trunk Control Scale; TIS= Trunk Impairment Scale; TST= Timed Stair Test; TUGT= Timed Up and Go Test
